# Membrane proteomic analysis of pancreatic cancer cells

**DOI:** 10.1186/1423-0127-17-74

**Published:** 2010-09-13

**Authors:** Xiaojun Liu, Min Zhang, Vay Liang W Go, Shen Hu

**Affiliations:** 1 UCLA School of Dentistry & Dental Research Institute, Los Angeles, CA, 90095, USA; 2 UCLA Center of Excellence in Pancreatic Diseases, Los Angeles, CA 90095, USA; 3 UCLA Jonsson Comprehensive Cancer Center, Los Angeles, CA 90095, USA

## Abstract

**Background:**

Pancreatic cancer is one of the most aggressive human tumors due to its high potential of local invasion and metastasis. The aim of this study was to characterize the membrane proteomes of pancreatic ductal adenocarcinoma (PDAC) cells of primary and metastatic origins, and to identify potential target proteins related to metastasis of pancreatic cancer.

**Methods:**

Membrane/membrane-associated proteins were isolated from AsPC-1 and BxPC-3 cells and identified with a proteomic approach based on SDS-PAGE, in-gel tryptic digestion and liquid chromatography with tandem mass spectrometry (LC-MS/MS). X! Tandem was used for database searching against the SwissProt human protein database.

**Results:**

We identified 221 & 208 proteins from AsPC-1 and BxPC-3 cells, respectively, most of which are membrane or membrane-associated proteins. A hundred and nine proteins were found in both cell lines while the others were present in either AsPC-1 or BxPC-3 cells. Differentially expressed proteins between two cell lines include modulators of cell adhesion, cell motility or tumor invasion as well as metabolic enzymes involved in glycolysis, tricarboxylic acid cycle, or nucleotide/lipid metabolism.

**Conclusion:**

Membrane proteomes of AsPC-1 (metastatic) and BxPC-3 (primary) cells are remarkably different. The differentially expressed membrane proteins may serve as potential targets for diagnostic and therapeutic interventions.

## Introduction

Pancreatic cancer is one of the most aggressive human malignancies. Despite the advances in therapeutic strategies including surgical techniques as well as local and systemic adjuvant therapies, the overall survival in patients with pancreatic cancer remains dismal and has not improved substantially over the past 30 years. Median survival from diagnosis is typically around 3 to 6 months, and the 5-year survival rate is less than 5%. As a result, in 2003, pancreatic cancer surpassed prostate cancer as the 4^th ^leading cause of cancer-related death in the US [1]. The main reason for the failure of current conventional therapy to cure pancreatic cancer and the major cause for cancer-related mortality in general, is the ability of malignant cells to detach from the primary tumor site and to develop metastasis in different regions of the same organ and in distant organs [2,3]. Pancreatic cancer usually causes no symptoms early on, leading to locally advanced or metastatic disease at time of diagnosis [4]. In this regard, it is important to identify the functional proteins that regulate/promote metastasis in pancreatic cancer. This would facilitate the development of strategies for therapeutic interventions and improved management of cancer patients.

The purpose of this study is to compare the membrane proteins expressed in pancreatic cancer cells of primary and metastatic origins using a proteomics approach. Membrane proteomics can be defined as analysis and characterization of entire complement of membrane proteins present in a cell under a specific biological condition [5,6]. In fact, membrane proteins account for more than two-thirds of currently known drug targets. Defining membrane proteomes is therefore important for finding potential drug targets. Membrane proteomics can also serve as a promising approach to human cancer biomarker discovery because membrane proteins are known to have implication in cell proliferation, cell adhesion, cell motility and tumor cell invasion [7-9].

## Materials and methods

### Cell culture

AsPC-1 and BxPC-3 cell lines were obtained from American Tissue Culture Collection (ATCC, Rockville, MD). These cell lines were initially generated from patients with pancreatic ductal adenocarcinoma (PDAC) [10-12]. The cells were maintained at 5% CO_2_-95% air, 37°C, and with RPMI 1640 (ATCC) containing 10% FBS, 100 μg/ml penicillin G and 100 mg/ml streptomycin. When the confluence reached 80-90%, the cells were harvested and washed with PBS for three times.

### Sample preparation

Membrane proteins from AsPC-1 and BxPC-3 cells were isolated with the ProteoExtract Native Membrane Protein Extraction Kit (EMD Chemicals, Gibbstown, NJ). In brief, the cell pellet was washed three times with the Washing Buffer, and then incubated with ice-cold Extract Buffer |at 4°C for 10 min under gentle agitation. After the pellet was centrifuged at 16,000 g for 15 min (4°C), the supernatant was discarded and 1 mL ice-cold Extract Buffer|| was added to the pellet. This membrane protein extraction step was allowed for 30 min at 4°C under gentle agitation. Then the supernatant was collected after centrifugation at 16,000 g for 15 min 4°C.

### SDS-PAGE and proteolytic cleavage

Total membrane protein concentration was measured with the 2-D Quant Kit (GE Healthcare, Piscataway, NJ). In total, 20 μg of membrane proteins from each cell line were loaded into a 4-12% NuPAGE Bis-Tris gel (Invitrogen, Carlsbad, CA) for SDS-PAGE separation. The gel was stained with the Simply Blue staining solution (Invitrogen) to visualize the proteins. Each gel was then cut into 15 sections evenly and proteolytic cleavage of proteins in each section was performed with enzyme-grade trypsin (Promega, Madison, WI) as previously described.

### Tandem MS and database searching

Liquid chromatography (LC) with tandem MS (LC/MS/MS) of peptides was performed using a NanoLC system (Eksigent Technologies, Dublin, CA) and a LTQ mass spectrometer (Thermo Fisher, Waltham, MA). Aliquots (5 μL) of the peptide digest derived from each gel slice were injected using an autosampler at a flow rate of 3.5 μL/min. The peptides were concentrated and desalted on a C_18 _IntegraFrit Nano-Precolumn (New Objective, Woburn, MA) for 10 min, then eluted and resolved using a C_18 _reversed-phase capillary column (New Objective). LC separation was performed at 400 nL/min with the following mobile phases: A, 5% acetonitrile/0.1%formic acid (v/v); B, 95% acetonitrile/0.1% formic acid (v/v). The chosen LC gradient was: from 5% to 15% B in 1 min, from 15% to 100% B in 40 min, and then maintained at 100%B for 15 min.

Database searches were performed using the X! Tandem search engine against the SwissProt protein sequence database. The search criteria were set with a mass accuracy of 0.4 Da and semi-style cleavage by trypsin. Proteins with two unique peptides are considered as positively identified.

### Western blot analysis

AsPC-1 and BxPC-3 cells were lysed with a lysis buffer containing 8 M urea, 2 M Thiourea and 4% CHAPS. Cell lysates with a total protein amount of 40 μg were separated with 8-12% NuPAGE gels at 100 V for about 2 hours and then transferred to polyvinylidene difluoride membrane using an iBlot system (Invitrogen, Carlsbad, CA, USA). After saturating with 2% slim milk, the blots were sequentially incubated with primary antibody (1:100 dilution) and horseradish peroxidase-conjugated antimouse IgG secondary antibody (1:1000 dilution, Applied Biological Materials Inc, Richmond, Canada). Anti-annexin A1 was obtained from Abcam (Cambridge, MA, USA) whereas anti-phosphoglycerate kinase 1 was obtained from Santa Cruz Biotechnology (Santa Cruz, CA, USA). Finally, the bands were visualized by enhanced chemiluminescence detection (Applied Biological Materials).

## Results

**Figure 1:**
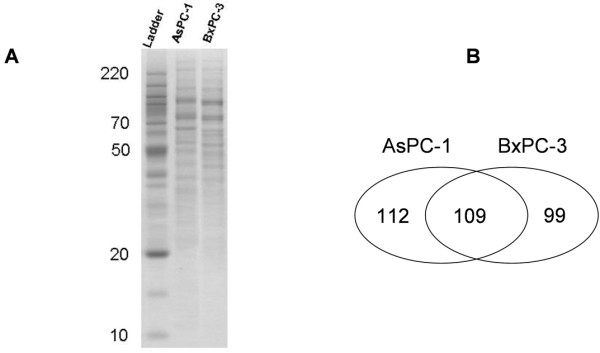
**Analysis and identification of membrane proteins in AsPC-1 and BxPC-3 cells using a proteomics approach based on SDS-PAGE, in-gel digestion and LC-MS/MS**. (A) Membrane proteins were isolated, separated with SDS-PAGE and detected with Simply Blue stain. The gel bands were then excised and digested with trypsin, and the resulting peptides were extracted for LC-MS/MS analysis. (B) 221 and 208 proteins were identified from AsPC-1 and BxPC-3 cells, respectively, with 109 proteins present in both cell lines.

**Figure 2:**
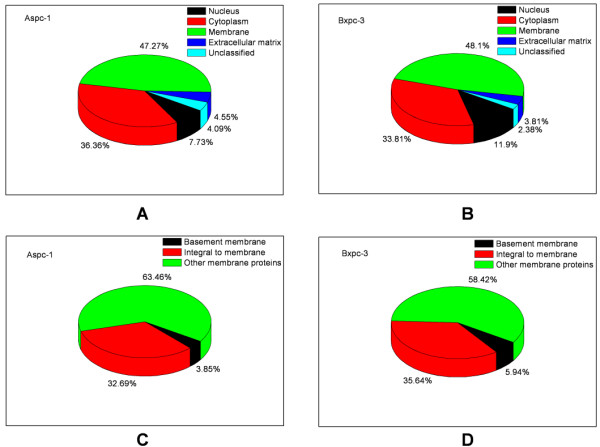
**Sorting of the identified proteins according to their subcellular localization**.

**Figure 3:**
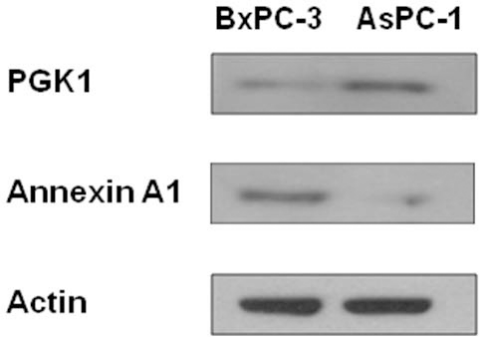
**Western blot analysis of Annexin A1 and phosphoglycerate kinase 1 (PGK1) between AsPC-1 and BxPC-3 cells**.

The purpose of this study was to demonstrate a membrane proteomic analysis of PDAC cells and to identify differentially expressed membrane proteins between primary and metastatic PDAC cells, which may have a potential role in metastasis of pancreatic cancer. Two PDAC cell lines, AsPC-1 and BxPC-3, were used in this study. AsPC-1 is a cell line of metastatic origin from a 62 year-old female Caucasian whereas BxPC-3 is a cell line of primary PDAC from a 61 year-old female Caucasian [10-12]. Membrane proteins of AsPC-1 and BxPC-3 cells were isolated and then resolved with SDS-PAGE (Figure 1A). Proteins in each gel slices were proteolytically cleaved and the resulting peptides were analyzed with LC-MS/MS. In total, we identified 221 and 208 membrane or membrane-associated proteins from AsPC-1 and BxPC-3 cells, respectively, based on at least 2 unique peptides. A hundred and nine proteins were present in both cell lines but others were only found in AsPC-1 or in BxPC-3 cells (Figure 1B). All the identified proteins and matched peptides from the two cell lines are summarized in Additional file 1, Tables S1 and S2. Proteins with single matched peptide were not tabulated although previous publications reported identification of membrane proteins based on single unique peptide [13,14]. The identified proteins were then sorted according to the Gene Ontology Annotation database (Figure 2). A hundred and four proteins were assigned as membrane proteins in AsPC-1 cells whereas 101 proteins were assigned as membrane proteins in BxPC-3 cells. Table 1 lists the "integral to membrane" proteins found in AsPC-1 and BxPC-3 cells. Besides the membrane proteins, the proteomic analysis also identified many membrane-associated proteins, e.g., extracellular matrix (ECM) proteins. To confirm the proteomic finding, we verified the differential levels of Annexin A1 and PGK1 between AsPC-1 and BxPC-3 cells using Western blotting (Figure 3). Annexin A1 was found to be over-expressed in BxPC-3 cells whereas phosphoglycerate kinase 1 was over-expressed in AsPC-1 cells, which agrees to the results obtained by the proteomic approach.

**Table 1:** Integral to membrane proteins identified in AsPC-1 & BxPC-3 cells

AsPC-1		BxPC-3	
Accession #	Protein name	Accession #	Protein name
1A25_HUMAN	HLA class I histocompatibility antigen, A-25 alpha chain	4F2_HUMAN	4F2 cell-surface antigen heavy chain
4F2_HUMAN	4F2 cell-surface antigen heavy chain	ACSL3_HUMAN	Long-chain-fatty-acid--CoA ligase 3
AAAT_HUMAN	Neutral amino acid transporter B(0)	ACSL4_HUMAN	Long-chain-fatty-acid--CoA ligase 4
ACSL5_HUMAN	Long-chain-fatty-acid--CoA ligase 5	ADT2_HUMAN	ADP/ATP translocase 2
ADT2_HUMAN	ADP/ATP translocase 2	ALK_HUMAN	ALK tyrosine kinase receptor precursor
ANPRC_HUMAN	Atrial natriuretic peptide clearance receptor	APMAP_HUMAN	Adipocyte plasma membrane-associated protein
AOFB_HUMAN	Amine oxidase [flavin-containing] B	AT1A1_HUMAN	Sodium/potassium-transporting ATPase subunit alpha-1
APMAP_HUMAN	Adipocyte plasma membrane-associated protein	CALX_HUMAN	Calnexin
AT1A1_HUMAN	Sodium/potassium-transporting ATPase subunit alpha-1 precursor	CEAM1_HUMAN	Carcinoembryonic antigen-related cell adhesion molecule 1
ATP7B_HUMAN	Copper-transporting ATPase 2	CEAM6_HUMAN	Carcinoembryonic antigen-related cell adhesion molecule 6
CALX_HUMAN	Calnexin	CKAP4_HUMAN	Cytoskeleton-associated protein 4
CEAM1_HUMAN	Carcinoembryonic antigen-related cell adhesion molecule 1	CLCN1_HUMAN	Chloride channel protein
CEAM6_HUMAN	Carcinoembryonic antigen-related cell adhesion molecule 6	CMC2_HUMAN	Calcium-binding mitochondrial carrier protein Aralar2
CMC2_HUMAN	Calcium-binding mitochondrial carrier protein Aralar2	CODA1_HUMAN	Collagen alpha-1(XIII) chain
CY1_HUMAN	Cytochrome c1, heme protein	CSMD2_HUMAN	CUB and sushi domain-containing protein 2
EGFR_HUMAN	Epidermal growth factor receptor precursor	EAA1_HUMAN	Excitatory amino acid transporter 1
FLNB_HUMAN	Filamin-B	GP124_HUMAN	Probable G-protein coupled receptor 124
FLRT1_HUMAN	Leucine-rich repeat transmembrane protein FLRT1	GRP78_HUMAN	78 kDa glucose-regulated protein
FZD8_HUMAN	Frizzled-8 precursor	HNRPM_HUMAN	Heterogeneous nuclear ribonucleoprotein M
GRP78_HUMAN	78 kDa glucose-regulated protein	ITAV_HUMAN	Integrin alpha-V
IL4RA_HUMAN	Interleukin-4 receptor alpha chain	KCNQ3_HUMAN	Potassium voltage-gated channel subfamily KQT member 3
IMMT_HUMAN	Mitochondrial inner membrane protein	L2HDH_HUMAN	L-2-hydroxyglutarate dehydrogenase
KCNK3_HUMAN	Potassium channel subfamily K member 3	M2OM_HUMAN	Mitochondrial 2-oxoglutarate/malate carrier protein
KTN1_HUMAN	Kinectin	MUC16_HUMAN	Mucin-16
LAMP1_HUMAN	Lysosome-associated membrane glycoprotein 1	MYOF_HUMAN	Myoferlin
LRC59_HUMAN	Leucine-rich repeat-containing protein 59	OST48_HUMAN	Dolichyl-diphosphooligosaccharide--protein glycosyltransferase 48 kDa subunit
MTCH2_HUMAN	Mitochondrial carrier homolog 2	PCD16_HUMAN	Protocadherin-16 precursor
MUC16_HUMAN	Mucin-16	PGRC1_HUMAN	Membrane-associated progesterone receptor component 1
MYOF_HUMAN	Myoferlin	PHB_HUMAN	Prohibitin
OST48_HUMAN	Dolichyl-diphosphooligosaccharide--protein glycosyltransferase 48 kDa subunit	PK1L1_HUMAN	Polycystic kidney disease protein 1-like 1
PHB_HUMAN	Prohibitin	PTPRZ_HUMAN	Receptor-type tyrosine-protein phosphatase zeta
S12A1_HUMAN	Solute carrier family 12 member 1	SSRD_HUMAN	Translocon-associated protein subunit delta precursor
SFXN3_HUMAN	Sideroflexin-3	TFR1_HUMAN	Transferrin receptor protein 1
VAT1_HUMAN	Synaptic vesicle membrane protein VAT-1 homolog	TMEDA_HUMAN	Transmembrane emp24 domain-containing protein 10
VDAC2_HUMAN	Voltage-dependent anion-selective channel protein 2	TOM40_HUMAN	Mitochondrial import receptor subunit TOM40 homolog
VMAT2_HUMAN	Synaptic vesicular amine transporter		

## Discussion

Metastasis is a highly organ-specific process, which requires multiple steps and interactions between tumor cells and the host. These include detachment of tumor cells from the primary tumor, intravasation into lymph and blood vessels, survival in the circulation, extravasation into target organs, and subsequent proliferation and induction of angiogenesis. Many proteins are critically involved in this process, such as cell-cell adhesion molecules (CAMs), members of the cadherins and, integrins, metalloproteinases (MMPs) and the urokinase plasminogen activator/urokinase plasminogen activator receptor (uPA/uPAR) system. As modulators of metastatic growth, these molecules can affect the local ECM, stimulate cell migration, and promote cell proliferation and tumor cell survivals [15]. Furthermore, hypoxia can drive genomic instability and lead to a more aggressive tumor phenotype [16,17], which may partially explain the highly metastatic nature of PDAC [18]. Last but not least, angiogenesis plays a critical role in invasion and metastasis in terms of tumor cell dissemination. Based on these new insights in mechanism of tumor invasion and metastasis, novel therapies are currently investigated for therapy of patients with pancreatic cancer [19-21]. Nevertheless, proteomic analysis of primary and metastatic PDAC is required to reveal additional functional proteins that regulate or promote tumor metastasis, as detailed in previous studies [22-24]. These signature molecules are predictors of metastatic risk and also provide a basis for the development of anti-metastatic therapy.

Our proteomic analysis has revealed a large number of differentially expressed membrane/surface proteins between metastatic and primary PDAC cells, and the validity of such a proteomic approach has been verified by Western blot analysis. In fact, the differential expression of membrane proteins between AsPC-1 and BxPC-3 can be observed from the SDS-PAGE patterns of membrane proteins from the two cell lines (Figure 1). The proteins showing differential levels include cadherins, catenin, integrins, galectins, annexins, collagens and many others, which are known to have roles in tumor cell adhesion or motility. Cadherins are a class of type-1 transmembrane proteins that depend on calcium ions to function. They play important roles in cell adhesion, ensuring that cells are bound together within tissues. Catenins, which are proteins found in complexes with cadherins, also mediate cell adhesion. Our study identified cadherins (protocadherin-16 and protocadherin alpha-12) and alpha-2 catenin in primary tumor cells (BxPC-3) but not in metastatic tumor cells (AsPC-1), suggesting a defect in cell-to-cell adhesion in metastatic AcPC-1 cells.

Integrins are members of a glycoprotein family that form heterodimeric receptors for ECM molecules. These proteins are involved in an adhesive function, and they provide traction for movement in cell motility [25]. In total, there are 18 α-subunits and 8 β-subunits, which are paired to form 24 different integrins through non-covalent bonding. Among these proteins, integrin-β_1_, α_2_, α_5_, and α_6 _represent major adhesion molecules for the adhesion of pancreatic cancer cells to ECM proteins [26]. In our study, integrin-β_1 _and integrin-β_4 _was found in both tumor cell lines while integrin α_2 _and α_5 _only identified in BxPC-3 cells. Collagens are major ECM proteins. Cell surface-expressed portion of collagens may serve as ligands for integrins, mediating cell-to-cell adhesion. Twelve members of collagen family were found in the BxPC-3 cells whereas only four members found in AsPC-1 cells.

Conversely, galectin-3 and galectin-4 were found in AsPC-1 but not in BxPC-3 cells. Galectins are carbohydrate-binding proteins and have an extremely high affinity for galactosides on cell surface and extracellular glycoproteins. Galectins, especially galectin-3, are modulators of cancer cell adhesion and invasiveness. Galectin-3 usually exists in cytoplasm, but can be secreted and bound on the cell surface by a variety of glycoconjugate ligands. Once localized to the cell surface, galectin-3 is capable of oligomerization, and the resultant cross-linking of surface glycoproteins into multimolecular complexes on the endothelial cell surface is reported to mediate the adhesion of tumor cells to the vascular endothelium [27]. Lysosome-associated membrane glycoprotein 1 (LAMP1) is a receptor for galectin-3, and was found on the cell surface of highly metastatic tumor cells [28]. Our study revealed LAMP1 in AsPC-1 cells but not in BxPC-3 cells. The cell surface-expressed portion of LAMP1 maybe serve as a ligand for galectin 3, mediating cell-cell adhesion and indirectly tumor spread. FKBP12-rapamycin complex-associated protein (a.k.a., mTOR) was also identified in AsPC-1 cells but not in BxPC-3 cells. mTOR is a downstream serine/threonine protein kinase of the phosphatidylinositol 3-kinase/Akt pathway that regulates cell proliferation, cell motility, cell survival, protein synthesis, and transcription. Rapamycin, a specific inhibitor of mTOR, suppresses lymphangiogenesis and lymphatic metastasis in PDAC cells [29].

The described proteomic approach is reproducible for analysis of membrane proteins in cultured pancreatic cancer cells. We observed consistent SDS-PAGE gel patterns for membrane proteins isolated from cultured AsPC-1 or BxPC-3 cells. To examine the reproducibility of LC-MS/MS for identification of membrane proteins, we repeated LC-MS/MS analysis of the peptides yielded from 3 gel bands. Compared to single LC-MS/MS, which identified 45 proteins in total, the duplicate LC-MS/MS analyses identified 47 proteins (~4% increase). This suggested that the observed difference in membrane protein profiles between the two PDAC cell lines is meaningful. Our adopted approach is valid to identify large membrane proteins, which are usually difficult to analyze with 2-D gel electrophoresis (2-DE) method. In AsPC-1 cells, 35% of the identified proteins have a molecular weight above 70 kDa, whereas 43% of the proteins are larger than 70 kDa in BxPC-3 cells. In addition to the proteins either present in AsPC-1 or in BxPC-3 cells, many other proteins were found in both cell types with a differential number of peptides matched. This may reflect the differential level of a protein between the two cell lines, although further verification is needed. Around 50% of the proteins identified in AsPC-1 and BxPC-3 cells are directly classified as membrane proteins, including a number of integral to membrane proteins and plasma membrane proteins. In addition, many mitochondrial inner membrane proteins were also identified from AsPC-1 (n = 21) and BxPC-3 (n = 13) cells. The mitochondrial inner membrane forms internal compartments known as cristae, which allow greater space for the proteins such as cytochromes to function properly and efficiently. The inner mitochondrial membrane contains mitochondria fusion and fission proteins, ATP synthases, transporter proteins regulating metabolite flux as well as proteins that perform the redox reactions of oxidative phosphorylation, many of which were identified in this study. Among the proteins that are not classified as membrane proteins, many are either membrane-associated proteins (e.g., kinases, G proteins, or enzymes) or proteins associated with other subcellular compartments such as mitochondria, endoplasmic reticulum (ER) or nucleus (e.g., histones, elongation factors, translation initiation factor and transcription factors) (Additional file 1, Table S1). It is commonly assumed that a protein is predominantly localized in a given cellular compartment where it exerts its specific function. However, a same protein may be localized at different cell compartments or travel between different organelles and therefore exert multiple cellular functions [30]. In fact, many proteins identified in mitochondria or ER are membrane or membrane-associated proteins.

In addition, many metabolic enzymes were identified from the two PDAC cell lines, reflecting the functional role of pancreas (Tables 2 and 3). These metabolic enzymes are involved in glycolysis, tricarboxylic acid cycle, gluconeogenesis, metabolism of nucleotides, lipids/fatty acids and amino acids, protein folding/unfolded protein response, and pantose phosphate shunt. Table 4 lists the small, membrane associated G proteins identified in AsPC-1 and BxPC-3 cells. Small GTPases regulate a wide variety of cellular processes, including growth, cellular differentiation, cell movement and lipid vesicle transport. RhoA, Rab-1A and Rab-10 were present in AsPC-1 cells whereas Rab-14 was found in BxPC-3 cells. As a proto-oncogene, RhoA regulates a signal transduction pathway linking plasma membrane receptors to the assembly of focal adhesions and actin stress fibers. On the other hand, Rab-1A regulates the 'ER-to-Golgi' transport, a bidirectional membrane traffic between the ER and Golgi apparatus which mediates the transfer of proteins by means of small vesicles or tubular-saccular extensions. Rab-10 is also involved in vesicular trafficking, particularly the directed movement of substances from the Golgi to early sorting endosomes. Mutated KRAS is a potent oncogene in PDAC. KRAS protein is usually tethered to cell membranes because of the presence of an isoprenyl group on its C-terminus. However, KRAS protein was not identified in this study, which might result from numerous mutations of the gene, hindering the matching of peptides based on molecular weight.

**Table 2:** Metabolic enzymes identified in AsPC-1 cells

Protein name	Accession #	Unique peptides	Total peptides	Mr (Kda)	PI	Biological process
2-oxoglutarate dehydrogenase E1 component, mitochondrial precursor	ODO1_HUMAN	8	18	115.9	6.39	Glycolysis
3,2-trans-enoyl-CoA isomerase, mitochondrial precursor	D3D2_HUMAN	3	13	32.8	8.8	Fatty acid metabolism; Lipid metabolism
3-hydroxyacyl-CoA dehydrogenase type-2	HCD2_HUMAN	6	10	26.9	7.65	Lipid metabolic process; tRNA processing
3-hydroxyisobutyrate dehydrogenase, mitochondrial precursor	3HIDH_HUMAN	7	16	35.3	8.38	Pentose-phosphate shunt; valine metabolic process
3-ketoacyl-CoA thiolase, peroxisomal precursor	THIK_HUMAN	3	4	44.3	8.76	Fatty acid metabolism; Lipid metabolism
3-mercaptopyruvate sulfurtransferase	THTM_HUMAN	3	7	33.2	6.13	Cyanate catabolic process
78 kDa glucose-regulated protein	GRP78_HUMAN	7	12	72.3	5.07	ER-associated protein catabolic process; ER unfolded protein response; ER regulation of protein folding
Acetyl-CoA acetyltransferase, mitochondrial precursor	THIL_HUMAN	2	6	45.2	8.98	Ketone body metabolism
Aconitate hydratase, mitochondrial	ACON_HUMAN	2	3	85.4	7.36	Tricarboxylic acid cycle
Acyl-protein thioesterase 1	LYPA1_HUMAN	2	2	24.7	6.29	Fatty acid metabolism; Lipid metabolism
Adenylate kinase 2, mitochondrial	KAD2_HUMAN	7	20	26.5	7.67	Nucleic acid metabolic process
ADP/ATP translocase 2	ADT2_HUMAN	5	11	32.9	9.76	Transmembrane transporter activity
Aldehyde dehydrogenase, mitochondrial	ALDH2_HUMAN	3	7	56.3	6.63	Alcohol metabolic process
Alpha-enolase	ENOA_HUMAN	2	2	47.1	7.01	Glycolysis
Amine oxidase B	AOFB_HUMAN	2	2	58.7	7.2	Oxidation reduction
Aspartate aminotransferase, mitochondrial	AATM_HUMAN	4	6	47.4	9.14	Lipid transport
ATP synthase subunit alpha, mitochondrial	ATPA_HUMAN	21	52	59.7	9.16	ATP synthesis
ATP synthase subunit d, mitochondrial	ATP5H_HUMAN	3	7	18.5	5.21	ATP synthesis; Ion transport
ATP synthase subunit b, mitochondrial	AT5F1_HUMAN	2	3	28.9	9.37	ATP synthesis
ATP synthase subunit beta, mitochondrial	ATPB_HUMAN	28	95	56.5	5.26	ATP synthesis
ATP synthase subunit f, mitochondrial	ATPK_HUMAN	2	2	10.9	9.7	ATP synthesis; Ion transport
ATP synthase subunit gamma, mitochondrial;	ATPG_HUMAN	3	6	33	9.23	ATP synthesis; proton transport
ATP synthase subunit O, mitochondrial	ATPO_HUMAN	6	11	23.3	9.97	ATP synthesis, ion transport; ATP catabolic process
Calcium-binding mitochondrial carrier protein Aralar2	CMC2_HUMAN	7	16	74.1	7.14	Mitochondrial aspartate and glutamate carrier
Citrate synthase, mitochondrial precursor	CISY_HUMAN	2	3	51.7	8.45	Tricarboxylic acid cycle
Cytochrome b5 type B	CYB5B_HUMAN	2	4	16.3	4.88	Electron transport
Cytochrome b-c1 complex subunit 1, mitochondrial	QCR1_HUMAN	6	12	52.6	5.94	Electron transport
Cytochrome b-c1 complex subunit 2, mitochondrial	QCR2_HUMAN	3	4	48.4	8.74	Aerobic respiration; electron transport chain; oxidative phosphorylation
Cytochrome c oxidase subunit 2	COX2_HUMAN	2	6	25.5	4.67	Electron transport chain
Cytochrome c1, heme protein, mitochondrial	CY1_HUMAN	5	10	35.4	9.15	Electron transport chain
Cytochrome c1, heme protein, mitochondrial	CY1_HUMAN	2	3	35.4	9.15	Electron transport chain
D-beta-hydroxybutyrate dehydrogenase, mitochondrial precursor	BDH_HUMAN	2	3	38.1	9.1	Oxidation reduction
Delta(3,5)-Delta(2,4)-dienoyl-CoA isomerase, mitochondrial	ECH1_HUMAN	4	10	35.8	8.16	Fatty acid metabolism; Lipid metabolism
Delta-1-pyrroline-5-carboxylate synthetase	P5CS_HUMAN	2	4	87.2	6.66	Amino-acid biosynthesis; Proline biosynthesis
Dihydrolipoyl dehydrogenase, mitochondrial	DLDH_HUMAN	7	16	54.1	7.95	Cell redox homeostasis
Dihydrolipoyllysine-residue acetyltransferase component of pyruvate dehydrogenase complex, mitochondrial	ODP2_HUMAN	3	5	65.7	7.96	Glycolysis
Dihydrolipoyllysine-residue succinyltransferase component of 2-oxoglutarate dehydrogenase complex, mitochondrial	ODO2_HUMAN	4	7	48.6	9.01	Tricarboxylic acid cycle
Electron transfer flavoprotein subunit alpha, mitochondrial	ETFA_HUMAN	2	5	35.1	8.62	Electron transport
Electron transfer flavoprotein subunit beta	ETFB_HUMAN	4	6	27.8	8.25	Electron transport
Endoplasmin	ENPL_HUMAN	16	28	92.4	4.76	ER-associated protein catabolic process; protein folding/transport; response to hypoxia
Enoyl-CoA hydratase, mitochondrial	ECHM_HUMAN	9	26	31.4	8.34	Fatty acid metabolism; Lipid metabolism
Glutamate dehydrogenase 1, mitochondrial;	DHE3_HUMAN	3	4	61.4	7.66	Glutamate metabolism
Glyceraldehyde-3-phosphate dehydrogenase	G3P_HUMAN	5	7	36	8.57	Glycolysis
Glycerol-3-phosphate dehydrogenase, mitochondrial precursor	GPDM_HUMAN	8	15	80.8	7.23	Glycolysis
Haloacid dehalogenase-like hydrolase domain-containing protein 3	HDHD3_HUMAN	3	4	28	6.21	Metabolic process phosphoglycolate phosphatase activity
Histidine triad nucleotide-binding protein 2	HINT2_HUMAN	2	3	17.2	9.2	Lipid synthesis; Steroid biosynthesis
Hyaluronidase-3	HYAL3_HUMAN	2	2	46.5		Carbohydrate metabolic process
Hydroxyacyl-coenzyme A dehydrogenase, mitochondrial precursor	HCDH_HUMAN	2	4	34.3	8.88	Fatty acid metabolism; Lipid metabolism
Isoleucyl-tRNA synthetase, mitochondrial precursor	SYIM_HUMAN	5	7	113.7	6.78	Protein biosynthesis
Isovaleryl-CoA dehydrogenase, mitochondrial	IVD_HUMAN|	2	2	46.3	8.45	Leucine catabolic process; Oxidation reduction
L-lactate dehydrogenase A chain	LDHA_HUMAN	3	5	36.7	8.84	Glycolysis
Lon protease homolog, mitochondrial	LONM_HUMAN	2	2	106.4	6.01	Required for intramitochondrial proteolysis
Long-chain-fatty-acid--CoA ligase 5;	ACSL5_HUMAN	2	4	75.9	6.49	Fatty acid metabolism; Lipid metabolism
Malate dehydrogenase, mitochondrial	MDHM_HUMAN	3	5	35.5	8.92	Tricarboxylic acid cycle; Glycolysis
Medium-chain specific acyl-CoA dehydrogenase, mitochondrial	ACADM_HUMAN	2	6	46.6	8.61	Fatty acid metabolism; Lipid metabolism
Mitochondrial carrier homolog 2	MTCH2_HUMAN	3	10	33.3	8.25	Transmembrane transport
Mitochondrial inner membrane protein	IMMT_HUMAN	2	2	83.6	6.08	Protein binding; Cell proliferation-inducing
NADH-cytochrome b5 reductase 3	NB5R3_HUMAN	3	3	34.2	7.18	Cholesterol biosynthesis; Lipid/steroid synthesis
Neutral alpha-glucosidase AB	GANAB_HUMAN	6	9	106.8	5.74	Carbohydrate metabolic process
Peptidyl-prolyl cis-trans isomerase A	PPIA_HUMAN	2	3	18	7.68	Protein folidng; Interspecies interation
Peroxiredoxin-5	PRDX5_HUMAN	2	5	22	8.85	Cell redox homeostasis
Phosphoenolpyruvate carboxykinase, mitochondrial	PPCKM_HUMAN	8	18	70.6	7.56	Gluconeogenesis
Phosphoglycerate kinase 1	PGK1_HUMAN	4	7	44.6	8.3	Glycolysis
Protein disulfide-isomerase	PDIA1_HUMAN	3	3	57.1	4.76	Cell redox homeostasis
Protein disulfide-isomerase A3	PDIA3_HUMAN	4	7	56.7	5.98	Cell redox homeostasis
Protein disulfide-isomerase A4	PDIA4_HUMAN	2	2	72.9	4.96	Cell redox homeostasis; Protein secretion
Protein disulfide-isomerase A6	PDIA6_HUMAN	2	3	48.1	4.95	Cell redox homeostasis; Protein folding
Protein ETHE1, mitochondrial	ETHE1_HUMAN	4	11	27.9	6.35	Metabolic homeostasis in mitochondria
Protein transport protein Sec16A	SC16A_HUMAN	2	2	233.4	5.4	ER-Golgi transport; Protein transport
Pyruvate dehydrogenase E1 component alpha subunit, somatic form	ODPA_HUMAN	2	4	43.3	8.35	Glycolysis
Pyruvate dehydrogenase E1 component subunit alpha, mitochondrial precursor	ODPAT_HUMAN	3	7	42.9	8.76	Glycolysis
Pyruvate dehydrogenase E1 component subunit beta, mitochondrial	ODPB_HUMAN	2	3	39.2	6.2	Glycolysis; Tricarboxylic acid cycle
Serine hydroxymethyltransferase, mitochondrial	GLYM_HUMAN	12	21	56	8.76	L-serine metabolic process; Glycine metabolic process; One-carbon metabolic process
Succinate dehydrogenase flavoprotein subunit, mitochondrial	DHSA_HUMAN	2	5	72.6	7.06	Electron transport; Tricarboxylic acid cycle
Succinyl-CoA ligase [GDP-forming] beta-chain, mitochondrial precursor	SUCB2_HUMAN	3	3	46.5	6.15	Succinyl-CoA metabolic process; Tricarboxylic acid cycle
Succinyl-CoA ligase [GDP-forming] subunit alpha, mitochondrial precursor	SUCA_HUMAN	2	5	35	9.01	Tricarboxylic acid cycle
Superoxide dismutase [Mn], mitochondrial	SODM_HUMAN	2	5	24.7	8.35	Elimination of radicals
Thioredoxin-dependent peroxide reductase	PRDX3_HUMAN	4	10	27.7	7.68	Cell redox homeostasis; Hydrogen peroxide catabolic process
Thiosulfate sulfurtransferase	THTR_HUMAN	2	3	33.4	6.77	Cyanate catabolic process
Trifunctional enzyme subunit alpha, mitochondrial	ECHA_HUMAN	17	46	82.9	9.16	Fatty acid metabolism; Lipid metabolism
Trifunctional enzyme subunit beta, mitochondrial	ECHB_HUMAN	6	12	51.3	9.45	Fatty acid metabolism
Trimethyllysine dioxygenase, mitochondrial	TMLH_HUMAN	2	3	49.5	7.64	Carnitine biosynthesis
Very long-chain specific acyl-CoA dehydrogenase, mitochondrial	ACADV_HUMAN	3	5	70.3	8.92	Fatty acid metabolism; Lipid metabolism

**Table 3:** Metabolic enzymes identified in BxPC-3 cells

Protein name	Accession #	Unique peptides	Total peptides	Mr (KDa)	PI	Biological process
2-oxoglutarate dehydrogenase E1 component, mitochondrial	ODO1_HUMAN	4	4	115.9	6.39	Glycolysis
3-ketoacyl-CoA thiolase, mitochondrial	THIM_HUMAN	2	4	41.9	8.32	Fatty acid metabolism Lipid metabolism
78 kDa glucose-regulated protein	GRP78_HUMAN	31	91	72.3	5.07	ER-associated protein catabolic process ER unfolded protein response ER regulation of protein folding
Adenylate kinase 2, mitochondrial	KAD2_HUMAN	4	7	26.5	7.67	Nucleotide/nucleic acid metabolic process
ADP/ATP translocase 2	ADT2_HUMAN	2	5	32.9	9.76	Transmembrane transporter activity
Alpha-aminoadipic semialdehyde dehydrogenase	AL7A1_HUMAN	2	2	55.3	6.44	Cellular aldehyde metabolic process; oxidation reduction
Alpha-enolase	ENOA_HUMAN	3	5	47.1	7.01	Glycolysis
Annexin A1	ANXA1_HUMAN	4	5	38.7	6.57	Anti-apoptosis; Exocytosis; Lipid metabolic process
Aspartate aminotransferase, mitochondrial precursor	AATM_HUMAN	2	7	47.4	9.14	Lipid transport
ATP synthase subunit alpha, mitochondrial	ATPA_HUMAN	3	6	59.7	9.16	ATP synthesis
ATP synthase subunit beta, mitochondrial	ATPB_HUMAN	4	13	56.5	5.26	ATP synthesis
ATP synthase subunit d, mitochondrial	ATP5H_HUMAN	2	4	18.5	5.21	ATP synthesis; Ion transport
ATP synthase subunit gamma, mitochondrial	ATPG_HUMAN	2	3	33	9.23	ATP synthesis; Proton transport
ATP synthase subunit O, mitochondrial	ATPO_HUMAN	2	3	23.3	9.97	ATP synthesis; Ion transport ATP catabolic process
Calcium-binding mitochondrial carrier protein Aralar2	CMC2_HUMAN	2	4	74.1	7.14	Mitochondrial aspartate and glutamate carrier
Citrate synthase, mitochondrial;	CISY_HUMAN	3	5	51.7	8.45	Tricarboxylic acid cycle
Cytochrome b-c1 complex subunit 1, mitochondrial	QCR1_HUMAN	3	5	52.6	5.94	Electron transport
Cytochrome b-c1 complex subunit 2, mitochondrial	QCR2_HUMAN	2	2	48.4	8.74	Aerobic respiration; Electron transport chain; Oxidative phosphorylation
Cytochrome c oxidase subunit 2	COX2_HUMAN	2	4	25.5	4.67	Electron transport chain
Cytochrome c oxidase subunit 5B, mitochondrial precursor	COX5B_HUMAN	2	2	13.7	9.07	Respiratory gaseous exchange
Delta(3,5)-Delta(2,4)-dienoyl-CoA isomerase, mitochondrial precursor	ECH1_HUMAN	2	6	35.8	8.16	Fatty acid metabolism; Lipid metabolism
Delta-1-pyrroline-5-carboxylate synthetase	P5CS_HUMAN	2	3	87.2	6.66	Amino-acid biosynthesis; Proline biosynthesis
Dihydrolipoyl dehydrogenase, mitochondrial	DLDH_HUMAN	5	13	54.1	7.95	Cell redox homeostasis
Dihydrolipoyllysine-residue succinyltransferase component of 2-oxoglutarate dehydrogenase complex, mitochondrial	ODO2_HUMAN	3	6	48.6	9.01	Tricarboxylic acid cycle
Electron transfer flavoprotein subunit alpha, mitochondrial	ETFA_HUMAN	3	7	35.1	8.62	Electron transport
Electron transfer flavoprotein subunit beta	ETFB_HUMAN	2	3	27.8	8.25	Electron transport
Endoplasmin	ENPL_HUMAN	16	31	92.4	4.76	ER-associated protein catabolic process; protein folding/transport; response to hypoxia
Enoyl-CoA hydratase, mitochondrial	ECHM_HUMAN	3	12	31.4	8.34	Fatty acid metabolism; Lipid metabolism
ERO1-like protein alpha precursor	ERO1A_HUMAN	2	3	54.4	5.48	Electron transport
Glucosidase 2 subunit beta	GLU2B_HUMAN	2	5	59.4	4.33	ER protein kinase cascade
Glutamate dehydrogenase 1, mitochondrial	DHE3_HUMAN	2	2	61.4	7.66	Glutamate metabolism
Glyceraldehyde-3-phosphate dehydrogenase	G3P_HUMAN	2	2	36	8.57	Glycolysis
Glycerol-3-phosphate dehydrogenase, mitochondrial	GPDM_HUMAN	2	4	80.8	7.23	Glycolysis
Heme oxygenase 2	HMOX2_HUMAN	2	4	36	5.31	Heme oxidation; Oxidation reduction; Response to hypoxia
Hexokinase-1	HXK1_HUMAN	2	3	102.4	6.36	Glycolysis
L-2-hydroxyglutarate dehydrogenase, mitochondrial	L2HDH_HUMAN	2	2	50.3	8.57	Cellular protein metabolic process; Oxidation reduction
Lon protease homolog, mitochondrial	LONM_HUMAN	2	2	106.4	6.01	Required for intramitochondrial proteolysis
Long-chain-fatty-acid--CoA ligase 3	ACSL3_HUMAN	2	3	80.4	8.65	Fatty acid metabolism; Lipid metabolism
Long-chain-fatty-acid--CoA ligase 4	ACSL4_HUMAN	2	3	79.1	8.66	Fatty acid metabolism; Lipid metabolism
Malate dehydrogenase, mitochondrial	MDHM_HUMAN	3	4	35.5	8.92	TCA glycolysis
Medium-chain specific acyl-CoA dehydrogenase, mitochondrial	ACADM_HUMAN|	2	3	46.6	8.61	Fatty acid metabolism; Lipid metabolism
Methylenetetrahydrofolate reductase	MTHR_HUMAN	2	2	74.5	5.22	Methionine metabolic process; Oxidation reduction
Mitochondrial 2-oxoglutarate/malate carrier protein	M2OM_HUMAN	2	2	34	9.92	Transport
Mitochondrial import receptor subunit TOM40 homolog	TOM40_HUMAN	3	3	37.9	6.79	Ion transport; Protein transport
Neutral alpha-glucosidase AB	GANAB_HUMAN	7	10	106.8	5.74	Carbohydrate metabolic process
Neutral cholesterol ester hydrolase 1	ADCL1_HUMAN	2	4	45.8	6.76	Lipid degradation
Ornithine aminotransferase, mitochondrial precursor	OAT_HUMAN	4	6	48.5	6.57	Mitochondrial matrix protein binding
Phosphoenolpyruvate carboxykinase, mitochondrial	PPCKM_HUMAN	2	3	70.6	7.56	Gluconeogenesis
Protein disulfide-isomerase	PDIA1_HUMAN	8	14	57.1	4.76	Cell redox homeostasis
Protein disulfide-isomerase A3	PDIA3_HUMAN	16	25	56.7	5.98	Cell redox homeostasis
Protein disulfide-isomerase A4	PDIA4_HUMAN	7	11	72.9	4.96	Cell redox homeostasis; Protein secretion
Protein disulfide-isomerase A6	PDIA6_HUMAN	2	4	48.1	4.95	Cell redox homeostasis; Protein folding
Pyruvate kinase isozymes M1/M2	KPYM_HUMAN	5	7	57.9	7.96	Glycolysis; Programmed cell death
Serine hydroxymethyltransferase, mitochondrial precursor	GLYM_HUMAN	2	4	56	8.76	L-serine metabolic process; Glycine metabolic process; One-carbon metabolic process
Sterol regulatory element-binding protein 2	SRBP2_HUMAN	2	2	123.6	8.72	Cholesterol metabolism; Lipid metabolism; Steroid metabolism;
Succinate dehydrogenase flavoprotein subunit, mitochondrial	DHSA_HUMAN	3	10	72.6	7.06	Electron transport; Tricarboxylic acid cycle
Succinyl-CoA:3-ketoacid-coenzyme A transferase 1	SCOT_HUMAN	2	5	56.1	7.13	Ketone body catabolic process
Sulfide:quinone oxidoreductase, mitochondrial	SQRD_HUMAN	6	9	49.9	9.18	Oxidation reduction
Superoxide dismutase [Mn], mitochondrial	SODM_HUMAN	2	5	24.7	8.35	Elimination of radicals
Transmembrane emp24 domain-containing protein 10	TMEDA_HUMAN	2	3	25	6.98	ER-Golgi protein transport
Trifunctional enzyme subunit alpha, mitochondrial	ECHA_HUMAN	4	7	82.9	9.16	Fatty acid metabolism; Lipid metabolism
Trifunctional enzyme subunit beta, mitochondrial	ECHB_HUMAN	2	4	51.3	9.45	Fatty acid metabolism

**Table 4:** A list of small G proteins identified in AsPC-1 and BxPC-3 cells

AsPC-1						
Ras-related protein Rab-1B	3	7	22.2		RAB1B_HUMAN	VVDNTTAKEF ADSLGIPFLE TSAK
						VVDNTTAKEF ADSLGIPFLE TSAK
						EFADSLGIPF LETSAK
						EFADSLGIPF LETSAK
						EFADSLGIPF LETSAK
						EFADSLGIPF LETSAK
						NATNVEQAFM TMAAEIK
Ras-related protein Rab-7a	3	5	23.5		RAB7A_HUMAN	DPENFPFVVL GNKIDLENR
						DPENFPFVVL GNKIDLENR
						DPENFPFVVL GNK
						EAINVEQAFQ TIAR
						EAINVEQAFQ TIAR
Ras-related protein Rab-1A	3	7	22.7		RAB1A_HUMAN	VVDYTTAKEF ADSLGIPFLE TSAK
						VVDYTTAKEF ADSLGIPFLE TSAK
						EFADSLGIPF LETSAK
						EFADSLGIPF LETSAK
						EFADSLGIPF LETSAK
						EFADSLGIPF LETSAK
						NATNVEQSFM TMAAEIK
Ras-related protein Rab-10;	2	6	22.5	8.58	RAB10_HUMAN	LLLIGDSGVG K
						LLLIGDSGVG K
						AFLTLAEDIL R
						AFLTLAEDIL R
						AFLTLAEDIL R
						AFLTLAEDIL R
Ras-related protein Rab-2A	3	3	23.5	6.08	RAB2A_HUMAN	YIIIGDTGVG K
						TASNVEEAFI NTAK
						IGPQHAATNA THAGNQGGQQ AGGGCC
Ras GTPase-activating-like protein IQGAP1	2	2	189.1		IQGA1_HUMAN	ILAIGLINEA LDEGDAQK
						FQPGETLTEI LETPATSEQE AEHQR
Transforming protein RhoA	2	3	21.8		RHOA_HUMAN	QVELALWDTA GQEDYDR
						QVELALWDTA GQEDYDR
						HFCPNVPIIL VGNKK
						
**BxPC-3**						

Ras-related protein Rab-2A	2	3	23.5	6.08	RAB2A_HUMAN	GAAGALLVYD ITR
						TASNVEEAFI NTAK
						TASNVEEAFI NTAK
Ras-related protein Rab-1B	3	8	22.2	5.55	RAB1B_HUMAN	VVDNTTAKEF ADSLGIPFLE TSAK
						VVDNTTAKEF ADSLGIPFLE TSAK
						VVDNTTAKEF ADSLGIPFLE TSAK
						EFADSLGIPF LETSAK
						EFADSLGIPF LETSAK
						EFADSLGIPF LETSAK
						EFADSLGIPF LETSAK
						NATNVEQAFM TMAAEIK
Ras-related protein Rab-7a	2	3	23.5	6.39	RAB7A_HUMAN	DPENFPFVVL GNK
						EAINVEQAFQ TIAR
						EAINVEQAFQ TIAR
Ras-related protein Rab-14	2	2	23.9	5.85	RAB14_HUMAN	TGENVEDAFL EAAKK
						TGENVEDAFL EAAK
Cell division control protein 42 homolog	2	3	21.3	5.76	CDC42_HUMAN	TPFLLVGTQI DLRDDPSTIE K
						TPFLLVGTQI DLRDDPSTIE K
						TPFLLVGTQI DLR
Guanine nucleotide-binding protein subunit beta-2	2	4	37.3	5.6	GBB2_HUMAN	SELEQLRQEA EQLR
						SELEQLRQEA EQLR
						KACGDSTLTQ ITAGLDPVGR
						KACGDSTLTQ ITAGLDPVGR

Some of the proteins identified from the current study may be further verified in clinical specimens as biomarkers for diagnostic/prognostic applications. Particularly, protein biomarkers may be used to classify pancreatic cancer patients for a better treatment decision. Cancer biomarker discovery is an intensive research area. Despite the fact that a large number of researchers are searching for cancer biomarkers, only a handful of protein biomarkers have been approved by the US Food and Drug Administration (FDA) for clinical use [31]. Interestingly, most of the FDA-approved protein biomarkers for human cancers are membrane proteins, including cancer antigen CA125 (ovarian), carcinoembryonic antigen (colon), epidermal growth factor receptor (colon), tyrosine-protein kinase KIT (gastrointestinal), HER2/NEU, CA15-3, CA27-29, Oestrogen receptor and progesterone receptor (breast) and bladder tumour-associated antigen (bladder) [31]. Similarly, most of the reported protein biomarkers in PDAC are of membrane origin or membrane-associated, including CA 19-9, CEA, CA 242, CA 72-4, KRAS, KAI1, CEA-related cell adhesion molecule 1 (CEACAM1), MUC1, MUC4, among many others [32-39]. For instance, CA 19-9 is a membrane carbohydrate antigen and the most commonly used biomarker in pancreatic cancers. As a cell adhesion molecule, CEA actually mediates the collagen binding of epithelial cells [40]. KAI1, a metastasis suppressor protein, belongs to the transmembrane 4 superfamily. It is up-regulated in early PDAC and down-regulated in metastatic PDAC [34]. The present study also identified CEA-related cell adhesion molecule 1, CEA-related cell adhesion molecule 6, 4F2 cell-surface antigen heavy chain (a.k.a., CD98), epidermal growth factor receptor (EGFR), hypoxia up-regulated protein 1, MUC16 and mTOR, which may be further verified in clinical specimens as biomarkers for PDAC.

In summary, we have demonstrated a proteomic approach for analysis and identification of membrane proteins in primary and metastatic PDAC cells. Many of the identified proteins are known to be modulators of cell-to-cell adhesion and tumor cell invasion. With the potential targets derived from the present study, we will next focus on promising candidates and explore their functional role in cell proliferation, apoptosis or metabolism in PDAC. Similar membrane proteomics approach can be applied to tissue specimens from patients with primary and metastatic tumors to reveal membrane protein targets for prognostic application or therapeutic intervention.

## Competing interests

The authors declare that they have no competing interests.

## Authors' contributions

SH conceived of the study, participated in its design and coordination and drafted the manuscript. XJL and MZ participated in the study design and collected the data. VLWG participated in the study design and critically reviewed the manuscript. All authors read and approved the final manuscript.

## Supplementary Material

Additional file 1 **Membrane and membrane-associated proteins identified in AsPC-1 cells (Table S1) and BxPC-3 cells (Table S2)**. Highlighted proteins were only found in AsPC-1 cells (Table S1) and BxPC-3 cells (Table S2). Click here for file
