# Supplementary material for: Membrane proteomic analysis of pancreatic cancer cells
Source: J Biomed Sci. 2010 Sep 13;17(1):74. doi: 10.1186/1423-0127-17-74 (PMC2949717; doi:10.1186/1423-0127-17-74)
Supplement: Additional file 1 — Membrane and membrane-associated proteins identified in AsPC-1 cells (Table S1) and BxPC-3 cells (Table S2). Highlighted proteins were only found in AsPC-1 cells (Table S1) and BxPC-3 cells (Table S2). [file 1423-0127-17-74-S1.PDF]

Supplemental Table 1. Membrane and membrane-associated proteins identified in AsPC-1 cells. Highlighted proteins were only found in AsPC-1 cells.

Accession #	Protein name	Unique peptides	Total peptides	Mr (KDa)	PI	Peptides identified	Subcellular localization
CH10_HUMAN	10 kDa heat shock protein	2	2	10.9	8.89	VLQATVVAVG SGSK	Mitochondrion
						VVLDDKDYFL FR	
ANXA2_HUMAN	Annexin A2	10	24	38.6	7.57	STVHEILCK	Plasma membrane
						STVHEILCK	
						AYTNFDAERD ALNIETAIKT K	
						AYTNFDAERD ALNIETAIK	
						AYTNFDAERD ALNIETAIK	
						GVDEVTIVNI LTNR	
						GVDEVTIVNI LTNR	
						GVDEVTIVNI LTNR	
						GVDEVTIVNI LTNR	
						GVDEVTIVNI LTNR	
						GVDEVTIVNI LTNR	
						TPAQYDASEL KASMK	
						TPAQYDASEL KASMK	
						TPAQYDASEL K	
						GLGTDEDSLI EIICSR	
						GLGTDEDSLI EIICSR	
						GLGTDEDSLI EIICSR	
						GLGTDEDSLI EIICSR	
						GLGTDEDSLI EIICSR	
						GLGTDEDSLI EIICSR	
						TDLEKDIISD TSGDFRK	
						TDLEKDIISD TSGDFRK	
						RAEDGSVIDY ELIDQDAR	
						SYSPYDMLES IR	
G3P_HUMAN	Glyceraldehyde-3-phosphate dehydrogenase	5	7	36	8.57	LVINGNPITI FQER	Membrane
						IISNASCTTN CLAPLAK	
						VIHDNFGIVE GLMTTVHAIT ATQK	
						VIHDNFGIVE GLMTTVHAIT ATQK	

						GALQNIIPAS TGAAK	
						LISWYDNEFG YSNR	
						LISWYDNEFG YSNR	
NPM_HUMAN	Nucleophosmin	2	6	32.6	4.64	MSVQPTVSLG GFEITPPVVL R	Cytosol
						MSVQPTVSLG GFEITPPVVL R	
						MSVQPTVSLG GFEITPPVVL R	
						MSVQPTVSLG GFEITPPVVL R	
						MSVQPTVSLG GFEITPPVVL R	
						MTDQEAIQDL WQWR	
ODPB_HUMAN	Pyruvate dehydrogenase E1 component subunit beta	2	3	39.2	6.2	IMEGPAFNFL DAPAVR	Mitochondrion
						IMEGPAFNFL DAPAVR	
						ILEDNSIPQV K	
ROA2_HUMAN	Heterogeneous nuclear ribonucleoproteins A2/B1	2	3	37.4	8.97	LFIGGLSFET TEESLR	Nucleus
						LFIGGLSFET TEESLR	
						GFGFVTFDDH DPVDKIVLQK	
ETFA_HUMAN	Electron transfer flavoprotein subunit alpha	2	5	35.1	8.62	QSTLVIAEHA NDSLAPITLN TITAATR	Mitochondrion
						QSTLVIAEHA NDSLAPITLN TITAATR	
						QSTLVIAEHA NDSLAPITLN TITAATR	
						GLLPEELTPL ILATQK	
						GLLPEELTPL ILATQK	
						GARTPKVPKQ GGMNWADLLP PPPAHPPPHS	
ROBO1_HUMAN	Roundabout homolog 1	2	2	180.8	5.7	NSEEYNISVD ESYDQEMP	Membrane
						GARTPKVPKQ GGMNWADLLP PPPAHPPPHS	
						NSEEYNISVD ESYDQEMP	
						VPKQGGMNWA DLLPPPPAHP PPHSNSEEYN	
						ISVDESYDQE MPCPVPP	
PRDX5_HUMAN	Peroxiredoxin-5	2	5	22	8.85	GVLFGVPGAF TPGCSK	Mitochondrion
						GVLFGVPGAF TPGCSK	
						ALNVEPDGTG LTCSLAPNII SQL	
						ALNVEPDGTG LTCSLAPNII SQL	
						ALNVEPDGTG LTCSLAPNII SQL	

SSRD_HUMAN	Translocon-associated protein subunit delta	2	3	19	5.76	FFDEESYSLL R	Endoplasmic reticulum membrane
						NNEDISIIPP LFTVSVDHR<br>NNEDISIIPP LFTVSVDHR	
HINT2_HUMAN	Histidine triad nucleotide-binding protein 2	2	3	17.2	9.2	ILDKSLPADI LYEDQQCLVF R	Mitochondrion
						ISQAEEEDQQ LLGHLLLVAK<br>ISQAEEEDQQ LLGHLLLVAK	
FIS1_HUMAN	Mitochondrial fission 1 protein	2	4	16.9	8.84	STQFEYAWCL VR	Mitochondrion outer membrane
						GIVLLEELLP K<br>GIVLLEELLP K<br>GIVLLEELLP K	
PRDX3_HUMAN	Thioredoxin-dependent peroxide reductase	4	10	27.7	7.68	PAVTQHAPYF K	Mitochondrion
						QISRDYGVLL EGSGLALR<br>QISRDYGVLL EGSGLALR<br>QISRDYGVLL EGSGLALR<br>DYGVLLEGSG LALR<br>DYGVLLEGSG LALR<br>DYGVLLEGSG LALR<br>DYGVLLEGSG LALR<br>GLFIIDPNGV IK<br>GLFIIDPNGV IK	
CYB5B_HUMAN	Cytochrome b5 type B	2	4	16.3	4.88	FLNEHPGGEE VLLEQAGVDA SESFEDVGHS SDAR<br>FLNEHPGGEE VLLEQAGVDA SESFEDVGHS SDAR<br>QYYIGDIHPS DLKPESGSK<br>QYYIGDIHPS DLKPESGSK	Mitochondrion outer membrane
PPIA_HUMAN	Peptidyl-prolyl cis-trans isomerase A	2	3	18	7.68	VNPTVFFDIA VDGEPLGR	Cytoplasm.
						VSFELFADKV PK	

						VSFELFADKV PK	
DOT1L_HUMAN	Histone-lysine N-methyltransferase	2	2	184.7	9.39	LEKLSGLAAP DY	Nucleus
						SLPISIPLST VQPNKLPVSI PLASVVLPSR	
ATPO_HUMAN	ATP synthase subunit O	6	11	23.3	9.97	LVRPPVQVYG IEGR	Mitochondrion inner membrane
						QNKLEQVEKE LLR	
						VAQILKEPK	
						VAASVLNPYV K	
						FSPLTTNLIN LLAENGR	
						FSPLTTNLIN LLAENGR	
						FSPLTTNLIN LLAENGR	
						FSPLTTNLIN LLAENGR	
						FSPLTTNLIN LLAENGR	
						FSPLTTNLIN LLAENGR	
						GEVPCTVTSA SPLEEATLSE LKTVLK	
ATP5H_HUMAN	ATP synthase subunit d, mitochondrial	3	7	18.5	5.21	YGLIPEEFFQ FLYPK	Mitochondrion inner membrane
						YGLIPEEFFQ FLYPK	
						HVVQSISTQQ EKETIAK	
RAB1B_HUMAN	Ras-related protein Rab-1B	3	7	22.2	5.55	VVDNTTAKEF ADSLGIPFLE TSAK	Membrane
						VVDNTTAKEF ADSLGIPFLE TSAK	
						EFADSLGIPF LETSAK	
						EFADSLGIPF LETSAK	
						EFADSLGIPF LETSAK	
						EFADSLGIPF LETSAK	
						NATNVEQAFM TMAAEIK	
RAB7A_HUMAN	Ras-related protein Rab-7a	3	5	23.5	6.39	DPENFPFVVL GNKIDLENR	Intracelluar membrane-bound organelle
						DPENFPFVVL GNKIDLENR	
						DPENFPFVVL GNK	
						EAINVEQAFQ TIAR	
						EAINVEQAFQ TIAR	

AT5F1_HUMAN	ATP synthase subunit b, mitochondrial	2	3	28.9	9.37	YGLIPEEFFQ FLYPK	Membrane
						YGLIPEEFFQ FLYPK	
						HVVQSISTQQ EKETIAK	
COX2_HUMAN	Cytochrome c oxidase subunit 2	2	6	25.5	4.67	LLDVDNRVVL PIEAPIR	Membrane
						MMITSQDVLH SWAVPTLGLK	
						MMITSQDVLH SWAVPTLGLK	
						MMITSQDVLH SWAVPTLGLK	
						MMITSQDVLH SWAVPTLGLK	
						MMITSQDVLH SWAVPTLGLK	
ECHM_HUMAN	Enoyl-CoA hydratase	9	26	31.4	8.34	ASGANFEYII AEKR	Mitochondrion
						ASGANFEYII AEK	
						ASGANFEYII AEK	
						GKNNTVGLIQ LNRPK	
						NNTVGLIQLN RPK	
						ALNALCDGLI DELNQALK	
						ALNALCDGLI DELNQALK	
						AQFAQPEILI GTIPGAGGTQ R	
						AQFAQPEILI GTIPGAGGTQ R	
						SLAMEMVLTG DRISAQDAK	
						SLAMEMVLTG DRISAQDAK	
						SLAMEMVLTG DRISAQDAK	
						SLAMEMVLTG DRISAQDAK	
						SLAMEMVLTG DRISAQDAK	
						SLAMEMVLTG DR	
						ICPVETLVEE AIQCAEK	
						ICPVETLVEE AIQCAEK	
						ICPVETLVEE AIQCAEK	
						ICPVETLVEE AIQCAEK	
						ICPVETLVEE AIQCAEK	
						ICPVETLVEE AIQCAEK	
						ICPVETLVEE AIQCAEK	
						ICPVETLVEE AIQCAEK	

						ICPVETLVEE AIQCAEK	
						ICPVETLVEE AIQCAEK	
						ICPVETLVEE AIQCAEK	
KAD2_HUMAN	Adenylate kinase 2	7	20	26.5	7.67	PSVPAAEPEY PK	Mitochondrial inner membrane
						LAENFCVCHL ATGDMLR	
						LVSDEMVVEL IEK	
						LVSDEMVVEL IEK	
						LVSDEMVVEL IEK	
						LVSDEMVVEL IEK	
						LVSDEMVVEL IEK	
						NGFLLDGFPR	
						NGFLLDGFPR	
						NGFLLDGFPR	
						NGFLLDGFPR	
						NGFLLDGFPR	
						NGFLLDGFPR	
						LDSVIEFSIP DSLLIR	
						LDSVIEFSIP DSLLIR	
						LQAYHTQTTP LIEYYR	
						LQAYHTQTTP LIEYYR	
						GIHSAIDASQ TPDVVFASIL AAFSK	
						GIHSAIDASQ TPDVVFASIL AAFSK	
						GIHSAIDASQ TPDVVFASIL AAFSK	
HCD2_HUMAN	3-hydroxyacyl-CoA dehydrogenase type-2	6	10	26.9	7.65	GLVAVITGGA SGLGLATAER	Plasma membrane
						GLVAVITGGA SGLGLATAER	
						LVGQGASAVL LDLPNSGGEA QAK	
						LVGQGASAVL LDLPNSGGEA QAK	
						LVGQGASAVL LDLPNSGGEA QAK	
						LGNNCVFAPA DVTSEKDVQT ALALAK	
						VLDVNLMGTF NVIR	
						VLDVNLMGTF NVIR	
						GVIINTASVA AFEGQVGQAA YSASK	

						GGIVGMTLPI AR	
CY1_HUMAN	Cytochrome c1, heme protein	5	10	35.4	9.15	SDLELHPPSY PWSHR	Mitochondrion inner membrane
						SDLELHPPSY PWSHR	
						GLLSSLDHTS IR	
						LFDYFPKPYP NSEAAR	
						AANNGALPPD LSYIVR	
						AANNGALPPD LSYIVR	
						AANNGALPPD LSYIVR	
						AANNGALPPD LSYIVR	
						AANNGALPPD LSYIVR	
						HGGEDYVFSL LTGYCEPPTG VSLR	
						HGGEDYVFSL LTGYCEPPTG VSLR	
D3D2_HUMAN	3,2-trans-enoyl-CoA isomerase	3	13	32.8	8.8	VLVEPDAGAG VAVMK	Mitochondrion inner membrane
						VLVEPDAGAG VAVMK	
						VLVEPDAGAG VAVMK	
						VLVEPDAGAG VAVMK	
						GVILTSDRPG VFSAGLDLTE MCGR	
						GVILTSDRPG VFSAGLDLTE MCGR	
						GVILTSDRPG VFSAGLDLTE MCGR	
						GVILTSDRPG VFSAGLDLTE MCGR	
						DADVQNFVSF ISK	
						DADVQNFVSF ISK	
						DADVQNFVSF ISK	
						DADVQNFVSF ISK	
						DADVQNFVSF ISK	
C1QBP_HUMAN	Complement component 1 Q subcomponent-binding protein	3	8	31.3	4.74	AFVDFLSDEI K	Plasma membrane
						AFVDFLSDEI K	
						VEEQEPELTS TPNFVVEVIK	
						VEEQEPELTS TPNFVVEVIK	
						ALVLDCHYPE DEVGQEDEAE SDIFSIR	

						ALVLDCHYPE DEVGQEDEAE SDIFSIR	
						ALVLDCHYPE DEVGQEDEAE SDIFSIR	
						ALVLDCHYPE DEVGQEDEAE SDIFSIR	
ETHE1_HUMAN	Protein ETHE1	4	11	27.9	6.35	EAVLIDPVLE TAPR	Mitochondrion
						EAVLIDPVLE TAPR	
						EAVLIDPVLE TAPR	
						EAVLIDPVLE TAPR	
						EAVLIDPVLE TAPR	
						LSGAQADLHI EDGDSIR	
						LTLSCEEFVK	
						LTLSCEEFVK	
						IMGNLNLPKP QQIDFAVPAN MR	
						IMGNLNLPKP QQIDFAVPAN MR	
						IMGNLNLPKP QQIDFAVPAN MR	
AAAT_HUMAN	Neutral amino acid transporter B(0)	3	5	56.6	5.34	SAAINASVGA AGSAENAPSK	Integral to plasma membrane
						STEPELIQVK SELPLDPLPV PTEEGNPLLK	
						STEPELIQVK SELPLDPLPV PTEEGNPLLK	
						SELPLDPLPV PTEEGNPLLK	
						SELPLDPLPV PTEEGNPLLK	
MDHM_HUMAN	Malate dehydrogenase	3	5	35.5	8.92	VAVLGASGGI GQPLSLLLK	Mitochondrion inner membrane
						VAVLGASGGI GQPLSLLLK	
						LTLYDIAHTP GVAADLSHIE TK	
						VDFPQDQLTA LTGR	
						VDFPQDQLTA LTGR	
ETFB_HUMAN	Electron transfer flavoprotein subunit beta	4	6	27.8	8.25	LGPLQVAR	Mitochondrion
						LAEKEKVDLV LLGK	
						LAEKEKVDLV LLGK	
						LPAVVTADLR	
						LPAVVTADLR	
						KIEVIKPGDL GVDLTSK	
SPRE_HUMAN	Sepiapterin reductase	3	6	28	8.25	TLAPLLASLL SPGSVLVLSA R	Cytoplasm

						TLAPLLASLL SPGSVLVLSA R	
						VPADLGAEAG LQQLLGALR	
						VPADLGAEAG LQQLLGALR	
						LLLINNAGSL GDVSK	
						LLLINNAGSL GDVSK	
CPNS1_HUMAN	Calpain small subunit 1	3	4	28.3	5.05	AGGGGGGGGG GGGGGGGGGG GTAMR	Plasma membrane
						GGGGGGGGGG GGGGGGGGGT AMRILGGVIS	
						AISEAAAQYN PEPPPPR	
						ILGGVISAIS EAAAQYNPEP PPPR	
						ILGGVISAIS EAAAQYNPEP PPPR	
HDHD3_HUMAN	Haloacid dehalogenase-like hydrolase domain-containing protein 3	3	4	28	6.21	LLTWDVKDTL LR	Mitochondrion
						AHGLEVEPSA LEQGFR	
						AHGLEVEPSA LEQGFR	
						AVGMHSFLVV GPQALDPVVR	
ERP29_HUMAN	Endoplasmic reticulum protein ERp29	2	3	29	6.77	GALPLDTVTF YK	Endoplasmic reticulum lumen
						SLNILTAFQK	
						SLNILTAFQK	
						MTTQLGPALV LGVALCLGCG QPLPQVPERP	
HYAL3_HUMAN	Hyaluronidase-3	2	2	46.5	8.55	FSVLWNVPSA HCE	Lysosome
						HLALAAYQIH HSLRPGFAGP AVLDWEEWCP	
						LWA	
1433G_HUMAN	14-3-3 protein gamma	2	2	28.3	4.8	TAFDDAIAEL DTLNEDSYK	Cytoplasm
						DSTLIMQLLR	
						ESHSPALADS ETPKATTQMV ITTTVGDPAP	
MUC16_HUMAN	Mucin-16	2	2	2351.9		STSMPVHGSS ETTNIK	Integral to membrane
						DTAHTEAMHA SMHTNTAVAN VGTSISGHES	
						QSSVPADSHT SKATSPMG	
ACTB_HUMAN	Actin, cytoplasmic 1	13	22	41.7	5.29	DDDIAALVVD NGSGMCK	Cytoplasm
						AGFAGDDAPR	
						AGFAGDDAPR	
						AVFPSIVGRP R	

						GIVTNWDDME K	
						GIVTNWDDME K	
						IWHHTFYNEL R	
						IWHHTFYNEL R	
						VAPEEHPVLL TEAPLNPK	
						VAPEEHPVLL TEAPLNPK	
						TTGIVMDSGD GVTHTVPIYE GYALPHAILR	
						GYSFTTTAER	
						LCYVALDFEQ EMATAASSSS LEK	
						LCYVALDFEQ EMATAASSSS LEK	
						LCYVALDFEQ EMATAASSSS LEK	
						FEQEMATAAS SSSLEK	
						SYELPDGQVI TIGNER	
						SYELPDGQVI TIGNER	
						DLYANTVLSG GTTMYPGIAD R	
						DLYANTVLSG GTTMYPGIAD R	
						DLYANTVLSG GTTMYPGIAD R	
						QEYDESGPSI VHR	
ACTC_HUMAN	Actin, alpha cardiac muscle 1	2	2	42	5.23	AGFAGDDAPR	Cytoplasm
						AGFAGDDAPR	
						AVFPSIVGRP R	
						YPIEHGIITN WDDMEK	
						IWHHTFYNEL R	
						IWHHTFYNEL R	
						SYELPDGQVI TIGNER	
						SYELPDGQVI TIGNER	
						FRCPETLFQP SFIGMESAGI HETTYNSIMK	
						CDIDIRKDL	
STML2_HUMAN	Stomatin-like protein 2	4	11	38.5	6.87	ILEPGLNILI PVLDR	Membrane
						ILEPGLNILI PVLDR	
						ILEPGLNILI PVLDR	
						ILEPGLNILI PVLDR	
						QAQILASEAE KAEQINQAAG EASAVLAK	

						ILAAALTQHN GDAAASLTVA EQYVSAFSK	
						ILAAALTQHN GDAAASLTVA EQYVSAFSK	
						ILAAALTQHN GDAAASLTVA EQYVSAFSK	
						ILAAALTQHN GDAAASLTVA EQYVSAFSK	
						ILAAALTQHN GDAAASLTVA EQYVSAFSK	
						ILAAALTQHN GDAAASLTVA EQYVSAFSK	
						DSNTILLPSN PGDVTSMVAQ AMGVYGALTK	
						DSNTILLPSN PGDVTSMVAQ AMGVYGALTK	
VAT1_HUMAN	Synaptic vesicle membrane protein VAT-1 homolog	4	6	41.9	5.88	CLVLTGFGGY DK	Integral to membrane
						CLVLTGFGGY DK	
						LPPLPVTPGM EGAGVVIAVG EGVSDR	
						LPPLPVTPGM EGAGVVIAVG EGVSDR	
						VVTYGMANLL TGPK	
						AVCGFHLGYL DGEVELVSGV VAR	
SUCB2_HUMAN	Succinyl-CoA ligase [GDP-forming] beta-chain	3	3	46.5	6.15	SCNGPVLVGS PQGGVDIEEV AASNPELIFK	Mitochondrion
						IDATQVEVNP FGETPEGQVV CFDAK	
						ILNNSGLPIT SAIDLEDAAK	
QCR2_HUMAN	Cytochrome b-c1 complex subunit 2	3	4	48.4	8.74	LPNGLVIASL ENYSPVSR	Mitochondrion inner membrane
						LPNGLVIASL ENYSPVSR	
						YEDFSNLGTT HLLR	
						AVAFQNPQTH VIENLHAAAY R	
ACADM_HUMAN	Medium-chain specific acyl-CoA dehydrogenase	2	6	46.6	8.61	AFTGFIVEAD TPGIQIGR	Mitochondrion
						AFTGFIVEAD TPGIQIGR	
						AFAGDIANQL ATDAVQILGG NGFNTEYPVE	
						AFAGDIANQL ATDAVQILGG NGFNTEYPVE	
						AFAGDIANQL ATDAVQILGG NGFNTEYPVE	
						AFAGDIANQL ATDAVQILGG NGFNTEYPVE	
HNRPF_HUMAN	Heterogeneous nuclear ribonucleoprotein F	2	5	45.6	5.38	ITGEAFVQFA SQELAEK	Nucleus
						ITGEAFVQFA SQELAEK	

						ATENDIYNFF SPLNPVR	
						ATENDIYNFF SPLNPVR	
						ATENDIYNFF SPLNPVR	
THIL_HUMAN	Acetyl-CoA acetyltransferase	2	6	45.2	8.98	TPIGSFLGSL SLLPATK	Mitochondrion inner membrane
						TPIGSFLGSL SLLPATK	
						IVAFADAAVE PIDFPIAPVY AASMVLK	
						IVAFADAAVE PIDFPIAPVY AASMVLK	
						IVAFADAAVE PIDFPIAPVY AASMVLK	
						IVAFADAAVE PIDFPIAPVY AASMVLK	
PDIA6_HUMAN	Protein disulfide-isomerase A6	2	3	48.1	4.95	TGEAIVDAAL SALR	Endoplasmic reticulum lumen
						TGEAIVDAAL SALR	
						ALDLFSDNAP PPELLEIINE DIAK	
EFTU_HUMAN	Elongation factor Tu	2	5	49.5	7.26	LLDAVDTYIP VPAR	Mitochondrion
						LLDAVDTYIP VPAR	
						LLDAVDTYIP VPAR	
						LLDAVDTYIP VPAR	
						DLEKPFLLPV EAVYSVPGR	
CISY_HUMAN	Citrate synthase	2	3	51.7	8.45	ASASSTNLKD ILADLIPK	Mitochondrion
						ALGVLAQLIW SR	
						ALGVLAQLIW SR	
1A25_HUMAN	HLA class I histocompatibility antigen, A-25 alpha chain	2	3	41.2	6.09	FIAVGYVDDT QFVR	Integral to membrane
						AEQWRAYLEG RCVEWLR	
						AEQWRAYLEG RCVEWLR	
IVD_HUMAN|	Isovaleryl-CoA dehydrogenase	2	2	46.3	8.45	FLQEHLAPK	Mitochondrion
						LAGGPLGLMQ AVLDHTIPYL HVREAFGQKI GHFQLMQGK	
APMAP_HUMAN	Adipocyte plasma membrane-associated protein	2	4	46.5	5.82	LFENQLVGPE SIAHIGDVMF TGTADGR	Integral to membrane

						LFENQLVGPE SIAHIGDVMF TGTADGR	
						LFENQLVGPE SIAHIGDVMF TGTADGR	
						FPNGVQLSPA EDFVLVAETT MAR	
						FPNGVQLSPA EDFVLVAETT MAR	
VDAC1_HUMAN	Voltage-dependent anion-selective channel protein 1	12	15	30.8	8.62	WNTDNTLGTE ITVEDQLAR	Plasma membrane
						GLKLTFDSSF SPNTGKK	
						LTFDSSFSPN TGKK	
						GALVLGYEGW LAGYQMNFET AK	
						GALVLGYEGW LAGYQMNFET AK	
						VTQSNFAVGY K	
						TDEFQLHTNV NDGTEFGGSI YQK	
						KLETAVNLAW TAGNSNTR	
						YQIDPDACFS AK	
						VNNSSLIGLG YTQTLKPGIK	
						LTLSALLDGK NVNAGGHK	
						LTLSALLDGK	
						LTLSALLDGK	
						LGLGLEFQA	
						LGLGLEFQA	
3HIDH_HUMAN	3-hydroxyisobutyrate dehydrogenase	7	16	35.3	8.38	EFQDAGEQVV SSPADVAEKA DRIITMLPTS INAIEAYSGA NGILKKV	Mitochondrion
						EFQDAGEQVV SSPADVAEKA DRIITMLPTS INAIEAYSGA NGILKKV	
						IITMLPTSIN AIEAYSGANG ILKK	
						IITMLPTSIN AIEAYSGANG ILKK	
						IITMLPTSIN AIEAYSGANG ILK	
						IITMLPTSIN AIEAYSGANG ILK	
						IITMLPTSIN AIEAYSGANG ILK	
						IITMLPTSIN AIEAYSGANG ILK	
						ICNNMLLAIS MIGTAEAMNL GIR	
						ICNNMLLAIS MIGTAEAMNL GIR	
						ICNNMLLAIS MIGTAEAMNL GIR	
						ICNNMLLAIS MIGTAEAMNL GIR	

						ICNNMLLAIS MIGTAEAMNL GIR	
						SPILLGSLAH QIYR	
						SPILLGSLAH QIYR	
						GSLAHQIYR	
						DFSSVFQFLR EEETF	
VDAC2_HUMAN	Voltage-dependent anion-selective channel protein 2	5	7	31.5	7.5	SCSGVEFSTS GSSNTDTGKV TGTLETK	Integral to membrane
						TGDFQLHTNV NDGTEFGGSI YQK	
						TGDFQLHTNV NDGTEFGGSI YQK	
						YQLDPTASIS AK	
						VNNSSLIGVG YTQTLRPGVK	
						LTLSALVDGK	
						LTLSALVDGK	
PHB2_HUMAN	Prohibitin-2	4	9	33.3	9.83	IGGVQQDTIL AEGLHFR	Membrane
						IPWFQYPIIY DIR	
						IPWFQYPIIY DIR	
						IVQAEGEAEA AK	
						IYLTADNLVL NLQDESFTR	
						IYLTADNLVL NLQDESFTR	
						IYLTADNLVL NLQDESFTR	
						IYLTADNLVL NLQDESFTR	
						IYLTADNLVL NLQDESFTR	
ANXA4_HUMAN	Annexin A4	3	6	35.9	5.84	GLGTDEDAII SVLAYR	Cytoplasm
						GLGTDEDAII SVLAYR	
						GAGTDEGCLI EILASR	
						GAGTDEGCLI EILASR	
						GAGTDEGCLI EILASR	
						SETSGSFEDA LLAIVK	
ADT2_HUMAN	ADP/ATP translocase 2	5	11	32.9	9.76	TDAAVSFAKD FLAGGVAAAI SKTAVAPIER	Integral to membrane
						TDAAVSFAKD FLAGGVAAAI SK	
						TDAAVSFAKD FLAGGVAAAI SK	
						TDAAVSFAKD FLAGGVAAAI SK	
						TDAAVSFAKD FLAGGVAAAI SK	

						DFLAGGVAAA ISK	
						DFLAGGVAAA ISK	
						EQGVLSFWRG NLANVIRYFP TQALNF	
						YFPTQALNFA FK	
						YFPTQALNFA FK	
						YFPTQALNFA FK	
SFXN3_HUMAN	Sideroflexin-3	4	8	35.5	9.26	GELPLDINIQ EPR	Integral to membrane
						QLGTAYVSAT TGAVATALGL KSLTKHLPPL	
						QLGTAYVSAT TGAVATALGL K	
						QLGTAYVSAT TGAVATALGL K	
						QLGTAYVSAT TGAVATALGL K	
						FVPFAAVAAA NCINIPLMR	
						FVPFAAVAAA NCINIPLMR	
						FVPFAAVAAA NCINIPLMR	
ECH1_HUMAN	Delta(3,5)-Delta(2,4)-dienoyl-CoA isomerase	4	10	35.8	8.16	GSSAQEEASG VALGEAPDHS YESLR	Mitochondrion
						CPKPVIAAVH GGCIGGGVDL VTACDIR	
						VIGNQSLVNE LAFTAR	
						VIGNQSLVNE LAFTAR	
						VIGNQSLVNE LAFTAR	
						VFPDKEVMLD AALALAAEIS SK	
						VFPDKEVMLD AALALAAEIS SK	
						VFPDKEVMLD AALALAAEIS SK	
						VFPDKEVMLD AALALAAEIS SK	
						VFPDKEVMLD AALALAAEIS SK	
THTM_HUMAN	3-mercaptopyruvate sulfurtransferase	3	7	33.2	6.13	ALVSAQWVAE ALR	Cytoplasm
						ALVSAQWVAE ALR	
						AGQPLQLLDA SWYLPK	
						AGQPLQLLDA SWYLPK	
						AGQPLQLLDA SWYLPK	
						DGIEPGHIPG TVNIPFTDFL SQEGLEK	
						DGIEPGHIPG TVNIPFTDFL SQEGLEK	

ATPG_HUMAN	ATP synthase subunit gamma	3	6	33	9.23	IYGLGSLALY EK	Mitochondrial inner membrane
						IYGLGSLALY EK<br>GLCGAIHSSI AK<br>ELIEIISGAA ALD<br>ELIEIISGAA ALD<br>ELIEIISGAA ALD	
PHB_HUMAN	Prohibitin	3	5	29.8	5.57	FGLALAVAGG VVNSALYNVD AGHR	integral to plasma membrane
						AAELIANSLA TAGDGLIELR<br>AAELIANSLA TAGDGLIELR<br>AAELIANSLA TAGDGLIELR<br>NITYLPAGQS VLLQLPQ	
NB5R3_HUMAN	NADH-cytochrome b5 reductase 3	3	3	34.2	7.18	LIDREIISHD TR	Membrane
						FALPSPQHIL GLPVGQHIYL SAR<br>SVGMIAGGTG ITPMLQVIR	
BDH_HUMAN	D-beta-hydroxybutyrate dehydrogenase	2	3	38.1	9.1	VSVVEPGNFI AATSLYSPES IQAIAK	Mitochodrial inner membrane
						VSVVEPGNFI AATSLYSPES IQAIAK<br>METYCSSGST DTSPVIDAVT HALTATTPYT	
HCDH_HUMAN	Hydroxyacyl-coenzyme A dehydrogenase	2	4	34.3	8.88	TLSTIATSTD AASVVHSTDL VVEAIVENLK	Mitochondrion
						TLSTIATSTD AASVVHSTDL VVEAIVENLK<br>LLVPYLMEAI R<br>LLVPYLMEAI R	
SUCA_HUMAN	Succinyl-CoA ligase [GDP-forming] subunit alpha	2	5	35	9.01	ISALQSAGVV VSMSPAQLGT TIYKEFEK	Mitochondrial inner membrane
						ISALQSAGVV VSMSPAQLGT TIYKEFEK<br>ISALQSAGVV VSMSPAQLGT TIYKEFEK<br>ISALQSAGVV VSMSPAQLGT TIYKEFEK<br>ISALQSAGVV VSMSPAQLGT TIYK	
LDHA_HUMAN	L-lactate dehydrogenase A chain	3	5	36.7	8.84	ATLKDQLIYN LLKEEQTPQN K	Cytoplasm

						ATLKDQLIYN LLK	
						LLIVSNPVDI LTYVAWK	
						LLIVSNPVDI LTYVAWK	
						LLIVSNPVDI LTYVAWK	
MTCH2_HUMAN	Mitochondrial carrier homolog 2	3	10	33.3	8.25	ADAASQVLLG SGLTILSQPL MYVK	Integral to membrane
						ADAASQVLLG SGLTILSQPL MYVK	
						ADAASQVLLG SGLTILSQPL MYVK	
						ADAASQVLLG SGLTILSQPL MYVK	
						ADAASQVLLG SGLTILSQPL MYVK	
						ADAASQVLLG SGLTILSQPL MYVK	
						ADAASQVLLG SGLTILSQPL MYVK	
						ADAASQVLLG SGLTILSQPL MYVK	
						QVCQLPGLFS YAQHIASIDG RR	
						VLQHYQESDK GEELGPGNVQ K	
THTR_HUMAN	Thiosulfate sulfurtransferase	2	3	33.4	6.77	EGHPVTSEPS RPEPAVFK	Plasma membrane
						GAVNMPFMDF LTEDGFEKGP EELR	
						GAVNMPFMDF LTEDGFEKGP EELR	
CO7A1_HUMAN	Collagen alpha-1(VII) chain	2	2	295	5.95	PGVGVPGSPG PPGPPGVK	Basement membrane
						DSAVILGPPG PRGAK	
ABHDB_HUMAN	Abhydrolase domain-containing protein 11	2	2	34.7	8.85	GGAEPRPLPL SYR	Unknown
						LIAVDISPVE STGVSHFATY VAAMR	
SSB_HUMAN	Single-stranded DNA-binding protein	3	5	17.2	9.59	ESETTTSLVL ER	Mitochondion
						QVEGKNPVTI FSLATNEMWR	
						QVEGKNPVTI FSLATNEMWR	
						QATTIIADNI IFLSDQTK	
						QATTIIADNI IFLSDQTK	
ATPB_HUMAN	ATP synthase subunit beta	28	95	56.5	5.26	ETRLVLEVAQ HLGESTVR	Mitochondrial inner membrane
						ETRLVLEVAQ HLGESTVR	

LVLEVAQHLG ESTVR
LVLEVAQHLG ESTVR
LVLEVAQHLG ESTVR
LVLEVAQHLG ESTVR
LVLEVAQHLG ESTVR
LVLEVAQHLG ESTVR
LVLEVAQHLG ESTVR
LVLEVAQHLG ESTVR
TIAMDGTEGL VR
TIAMDGTEGL VR
TIAMDGTEGL VR
GQKVLDSGAP IKIPVGPETL GR
GQKVLDSGAP IKIPVGPETL GR
VLDSGAPIKI PVGPETLGR
VLDSGAPIKI PVGPETLGR
VLDSGAPIKI PVGPETLGR
VLDSGAPIKI PVGPETLGR
VLDSGAPIKI PVGPETLGR
VLDSGAPIKI PVGPETLGR
IMNVIGEPID ERGPIKTK
IMNVIGEPID ERGPIKTK
IMNVIGEPID ERGPIK
IMNVIGEPID ERGPIK
IMNVIGEPID ERGPIK
QFAPIHAEAP EFMEMSVEQE ILVTGIK
QFAPIHAEAP EFMEMSVEQE ILVTGIK
QFAPIHAEAP EFMEMSVEQE ILVTGIK
QFAPIHAEAP EFMEMSVEQE ILVTGIK
QFAPIHAEAP EFMEMSVEQE ILVTGIK
VVDLLAPYAK
VVDLLAPYAK
GGKIGLFGGA GVGK
IGLFGGAGVG K
IGLFGGAGVG K

TVLIMELINN VAK
TVLIMELINN VAK
TVLIMELINN VAK
TVLIMELINN VAK
TVLIMELINN VAK
TVLIMELINN VAK
TVLIMELINN VAK
TVLIMELINN VAK
TVLIMELINN VAK
TVLIMELINN VAK
TVLIMELINN VAK
TVLIMELINN VAK
TVLIMELINN VAK
TVLIMELINN VAK
TVLIMELINN VAK
TVLIMELINN VAK
AHGGYSVFAG VGER
AHGGYSVFAG VGER
AHGGYSVFAG VGER
EGNDLYHEMI ESGVINLK
EGNDLYHEMI ESGVINLK
VALVYGQMNE PPGAR
VALVYGQMNE PPGAR
VALTGLTVAE YFR
VALTGLTVAE YFR
VALTGLTVAE YFR
DQEGQDVLLF IDNIFR
DQEGQDVLLF IDNIFR
FTQAGSEVSA LLGR
FTQAGSEVSA LLGR
FTQAGSEVSA LLGR
FTQAGSEVSA LLGR
IPSAVGYQPT LATDMGTMQE R

KGSITSVQAI YVPADDLTDP APATTFAHLD
ATTVLSR
KGSITSVQAI YVPADDLTDP APATTFAHLD
ATTVLSR
GSITSVQAIY VPADDLTDPA PATTFAHLDA
TTVLSR
GSITSVQAIY VPADDLTDPA PATTFAHLDA
TTVLSR
PAPATTFAHL DATTVLSR
PAPATTFAHL DATTVLSR
AIAELGIYPA VDPLDSTSR
AIAELGIYPA VDPLDSTSR
AIAELGIYPA VDPLDSTSR
AIAELGIYPA VDPLDSTSR
AIAELGIYPA VDPLDSTSR
AIAELGIYPA VDPLDSTSR
AIAELGIYPA VDPLDSTSR
AIAELGIYPA VDPLDSTSR
IMDPNIVGSE HYDVAR
IMDPNIVGSE HYDVAR
PNIVGSEHYD VAR
SLQDIIAILG MDELSEEDKL TVSR
SLQDIIAILG MDELSEEDKL TVSR
SLQDIIAILG MDELSEEDKL TVSR
SLQDIIAILG MDELSEEDKL TVSR
FLSQPFQVAE VFTGHMGK
FLSQPFQVAE VFTGHMGK
FLSQPFQVAE VFTGHMGK
GFQQILAGEY DHLPEQAFYM VGPIEEAVAK
GFQQILAGEY DHLPEQAFYM VGPIEEAVAK
GFQQILAGEY DHLPEQAFYM VGPIEEAVAK
GFQQILAGEY DHLPEQAFYM VGPIEEAVAK
GFQQILAGEY DHLPEQAFYM VGPIEEAVAK

ATPA_HUMAN	ATP synthase subunit alpha	21	52	59.7	9.16	QKTGTAEMSS ILEER	Mitochondrial inner membrane
						QKTGTAEMSS ILEER	
						QKTGTAEMSS ILEER	
						ILGADTSVDL EETGRVLSIG DGIAR	
						ILGADTSVDL EETGRVLSIG DGIAR	
						ILGADTSVDL EETGRVLSIG DGIAR	
						ILGADTSVDL EETGRVLSIG DGIAR	
						ILGADTSVDL EETGR	
						ILGADTSVDL EETGR	
						VLSIGDGIAR	
						VLSIGDGIAR	
						NVQAEEMVEF SSGLK	
						NVQAEEMVEF SSGLK	
						GMSLNLEPDN VGVVVFGNDK LIK	
						RTGAIVDVPV GEELLGR	
						TGAIVDVPVG EELLGR	
						TGAIVDVPVG EELLGR	
						TGAIVDVPVG EELLGR	
						TGAIVDVPVG EELLGR	
						TGAIVDVPVG EELLGR	
						TGAIVDVPVG EELLGR	
						VVDALGNAID GK	
						RVGLKAPGII PR	
						VGLKAPGIIP R	
						VGLKAPGIIP R	
						AVDSLVPIGR	
						AVDSLVPIGR	
						QTGKTSIAID TIINQKR	
						QTGKTSIAID TIINQKR	
						TSIAIDTIIN QKR	
						TSIAIDTIIN QKR	
						TSIAIDTIIN QK	
						TSIAIDTIIN QK	

						GIRPAINVGL SVSR	
						GIRPAINVGL SVSR	
						GIRPAINVGL SVSR	
						EVAAFAQFGS DLDAATQQLL SR	
						EVAAFAQFGS DLDAATQQLL SR	
						EVAAFAQFGS DLDAATQQLL SR	
						EVAAFAQFGS DLDAATQQLL SR	
						EVAAFAQFGS DLDAATQQLL SR	
						EVAAFAQFGS DLDAATQQLL SR	
						EVAAFAQFGS DLDAATQQLL SR	
						FENAFLSHVV SQHQALLGTI R	
						FENAFLSHVV SQHQALLGTI R	
						ISEQSDAKLK EIVTNFLAGF EA	
						ISEQSDAKLK EIVTNFLAGF EA	
						LKEIVTNFLA GFEA	
						LKEIVTNFLA GFEA	
						LKEIVTNFLA GFEA	
						LKEIVTNFLA GFEA	
						EIVTNFLAGF EA	
GLYM_HUMAN	Serine hydroxymethyltransferase	12	21	56	8.76	GLELIASENF CSR	Mitochondrion
						AALEALGSCL NNKYSEGYPG KR	
						YYGGAEVVDE IELLCQR	
						YYGGAEVVDE IELLCQR	
						ISATSIFFES MPYKLNPK	
						ISATSIFFES MPYKLNPK	
						ISATSIFFES MPYKLNPK	
						ISATSIFFES MPYK	
						TGLIDYNQLA LTAR	
						TGLIDYNQLA LTAR	
						TGLIDYNQLA LTAR	
						TGLIDYNQLA LTAR	
						LIIAGTSAYA R 230lidy	
						AHLLADMAHI SGLVAAK	

						GYSLVSGGTD NHLVLVDLRP K	
						GYSLVSGGTD NHLVLVDLRP K	
						VLELVSITAN KNTCPGDR	
						VLELVSITAN KNTCPGDR	
						VVDFIDEGVN IGLEVK	
						VVDFIDEGVN IGLEVK	
						LQDFKSFLLK DSETSQR	
DLDH_HUMAN	Dihydrolipoyl dehydrogenase	7	16	54.1	7.95	ADQPIDADVT VIGSGPGGYV AAIK	Mitochondrion
						ADQPIDADVT VIGSGPGGYV AAIK	
						ADQPIDADVT VIGSGPGGYV AAIK	
						TVCIEKNETL GGTCLNVGCI PSK	
						TVCIEKNETL GGTCLNVGCI PSK	
						NQVTATKADG GTQVIDTK	
						NLGLEELGIE LDPR	
						NLGLEELGIE LDPR	
						NLGLEELGIE LDPR	
						IPNIYAIGDV VAGPMLAHK	
						IPNIYAIGDV VAGPMLAHK	
						IPNIYAIGDV VAGPMLAHK	
						IPNIYAIGDV VAGPMLAHK	
						IPNIYAIGDV VAGPMLAHK	
						SEEQLKEEGI EYK	
						VCHAHPTLSE AFR	
CH60_HUMAN	60 kDa heat shock protein	5	10	61	5.7	ALMLQGVDLL ADAVAVTMGP K	Mitochondrial inner membrane
						ALMLQGVDLL ADAVAVTMGP K	
						ALMLQGVDLL ADAVAVTMGP K	
						AAVEEGIVLG GGCALLR	
						AAVEEGIVLG GGCALLR	
						CIPALDSLTP ANEDQKIGIE IIKR	
						TALLDAAGVA SLLTTAEVVV TEIPKEEKD	
						TALLDAAGVA SLLTTAEVVV TEIPKEEKD	
						TALLDAAGVA SLLTTAEVVV TEIPKEEK	

						TALLDAAGVA SLLTTAEVVV TEIPKEEK	
QCR1_HUMAN	Cytochrome b-c1 complex subunit 1	6	12	52.6	5.94	TATFAQALQF VPETQVSLLD NGLR	Mitochondrial inner membrane
						TATFAQALQF VPETQVSLLD NGLR	
						AVELLGDIVQ NCSLEDSQIE KER	
						AVELLGDIVQ NCSLEDSQIE KER	
						DVVFNYLHAT AFQGTPLAQA VEGPSENVR	
						DVVFNYLHAT AFQGTPLAQA VEGPSENVR	
						DVVFNYLHAT AFQGTPLAQA VEGPSENVR	
						ADLTEYLSTH YKAPR	
						ADLTEYLSTH YKAPR	
						MVLAAAGGVE HQQLLDLAQK	
						MVLAAAGGVE HQQLLDLAQK	
						LCTSATESEV AR	
ECHB_HUMAN	Trifunctional enzyme subunit beta	6	12	51.3	9.45	TPFLLSGTSY KDLMPHDLAR	Mitochondrion envelope
						TPFLLSGTSY KDLMPHDLAR	
						AALTGLLHR	
						AALTGLLHR	
						LAAAFAVSRL EQDEYALR	
						LAAAFAVSRL EQDEYALR	
						AQDEGLLSDV VPFK	
						DFMYVSQDPK DQLLLGPTYA TPK	
						DFMYVSQDPK DQLLLGPTYA TPK	
						EGGQYGLVAA CAAGGQGHAM IVEAYPK	
						EGGQYGLVAA CAAGGQGHAM IVEAYPK	
						EGGQYGLVAA CAAGGQGHAM IVEAYPK	
ODO2_HUMAN	Dihydrolipoyllysine-residue succinyltransferase component of 2-oxoglutarate dehydrogenase complex	4	7	48.6	9.01	TPAFAESVTE GDVRWEK	Plasma membrane
						TPAFAESVTE GDVRWEK	
						LGFMSAFVK	
						ASAFALQEQP VVNAVIDDTT K	

						ASAFALQEQP VVNAVIDDTT K	
						ASAFALQEQP VVNAVIDDTT K	
						AAVEDPRVLL LDL	
TBA1B_HUMAN	Tubulin alpha-1B chain	4	6	50.1	4.94	TIGGGDDSFN TFFSETGAGK	Microtubule
						AVFVDLEPTV IDEVR	
						AVFVDLEPTV IDEVR	
						QLFHPEQLIT GKEDAANNYA R	
						QLFHPEQLIT GKEDAANNYA R	
						LISQIVSSIT ASLR	
ALDH2_HUMAN	Aldehyde dehydrogenase	3	7	56.3	6.63	VAEQTPLTAL YVANLIK	Mitochondrion
						VAEQTPLTAL YVANLIK	
						VAEQTPLTAL YVANLIK	
						TFVQEDIYDE FVER	
						TFVQEDIYDE FVER	
						ANNSTYGLAA AVFTK	
						ANNSTYGLAA AVFTK	
CALR_HUMAN	Calreticulin	4	7	48.1	4.29	EPAVYFKEQF LDGDGWTSR	Cytoplasm
						EPAVYFKEQF LDGDGWTSR	
						CKDDEFTHLY TLIVRPDNTY EVK	
						CKDDEFTHLY TLIVRPDNTY EVK	
						IDNSQVESGS LEDDWDFLPP KK	
						SGTIFDNFLI TNDEAYAEEF GNETWGVTK	
						SGTIFDNFLI TNDEAYAEEF GNETWGVTK	
HNRPK_HUMAN	Heterogeneous nuclear ribonucleoprotein K	3	6	50.9	5.39	IILDLISESP IK	Nucleus
						IILDLISESP IK	
						GSYGDLGGPI ITTQVTIPK	
						GSYGDLGGPI ITTQVTIPK	
						IITITGTQDQ IQNAQYLLQN SVK	
						IITITGTQDQ IQNAQYLLQN SVK	
DHE3_HUMAN	Glutamate dehydrogenase 1	3	4	61.4	7.66	AKPYEGSILE ADCDILIPAA SEK	Mitochondrion
						IIAEGANGPT TPEADKIFLE R	
						IIAEGANGPT TPEADKIFLE R	
						ISGASEKDIV HSGLAYTMER	

TBB2C_HUMAN	Tubulin beta-2C chain	3	5	49.8	4.79	SGPFGQIFRP DNFVFGQSGA GNNWAK	Microtubule
						SGPFGQIFRP DNFVFGQSGA GNNWAK	
						FGQSGAGNNW AK	
						ALTVPELTQQ MFDAK	
						ALTVPELTQQ MFDAK	
ENOA_HUMAN	Alpha-enolase	2	2	47.1	7.01	DATNVGDEGG FAPNILENKE GLELLK 2	Plasma membrane
						YISPDQLADL YK	
KCNK3_HUMAN	Potassium channel subfamily K member 3	2	2	43.5	9.26	NGQAGGGGGG GSAHTT	Integral to membrane
						NGQAGGGGGG GSAH	
PDIA3_HUMAN	Protein disulfide-isomerase A3	4	7	56.7	5.98	SDVLELTDDN FESR	Endoplasmic reticulum lumen
						TFSHELSDFG LESTAGEIPV VAIR	
						LSKDPNIVIA K	
						ELSDFISYLQ R	
						ELSDFISYLQ R	
						ELSDFISYLQ R	
						ELSDFISYLQ R	
PDIA1_HUMAN	Protein disulfide-isomerase	3	3	57.1	4.76	QFLQAAEAID DIPFGITSNS DVFSK	Plasma membrane
						HNQLPLVIEF TEQTAPK	
						YKPESEELTA ER	
GRP75_HUMAN	Stress-70 protein	26	87	73.6	5.87	ASEAIKGAVV GIDLGTTNSC VAVMEGK	Cell surface mitochondrion
						ASEAIKGAVV GIDLGTTNSC VAVMEGK	
						TTPSVVAFTA DGERLVGMPA KR	
						TTPSVVAFTA DGERLVGMPA KR	
						TTPSVVAFTA DGERLVGMPA KR	
						TTPSVVAFTA DGER	
						TTPSVVAFTA DGER	
						QAVTNPNNTF YATKR	
						QAVTNPNNTF YATKR	
						QAVTNPNNTF YATKR	
						QAVTNPNNTF YATKR	
						LYSPSQIGAF VLMK	
						LYSPSQIGAF VLMK	

LYSPSQIGAF VLMK
LYSPSQIGAF VLMK
LYSPSQIGAF VLMK
LYSPSQIGAF VLMK
LYSPSQIGAF VLMK
LYSPSQIGAF VLMK
LYSPSQIGAF VLMK
LYSPSQIGAF VLMK
MKETAENYLG HTAK
NAVITVPAYF NDSQR
NAVITVPAYF NDSQR
NAVITVPAYF NDSQR
NAVITVPAYF NDSQR
QATKDAGQIS GLNVLR
QATKDAGQIS GLNVLR
QATKDAGQIS GLNVLR
DAGQISGLNV LR
DAGQISGLNV LR
DAGQISGLNV LR
VINEPTAAAL AYGLDK
VINEPTAAAL AYGLDK
GVFEVKSTNG DTFLGGEDFD QALLR
GVFEVKSTNG DTFLGGEDFD QALLR
STNGDTFLGG EDFDQALLR
STNGDTFLGG EDFDQALLR
STNGDTFLGG EDFDQALLR
STNGDTFLGG EDFDQALLR
STNGDTFLGG EDFDQALLR
STNGDTFLGG EDFDQALLR
ETGVDLTKDN MALQR
ETGVDLTKDN MALQR
ETGVDLTKDN MALQR
ETGVDLTKDN MALQR
AKCELSSSVQ TDINLPYLTM DSSGPK

AKCELSSSVQ TDINLPYLTM DSSGPK
AKCELSSSVQ TDINLPYLTM DSSGPK
AQFEGIVTDL IRR
AQFEGIVTDL IRR
AQFEGIVTDL IR
AQFEGIVTDL IR
AQFEGIVTDL IR
AQFEGIVTDL IR
AMQDAEVSKS DIGEVILVGG MTR
AMQDAEVSKS DIGEVILVGG MTR
AMQDAEVSKS DIGEVILVGG MTR
AMQDAEVSKS DIGEVILVGG MTR
AMQDAEVSKS DIGEVILVGG MTR
SDIGEVILVG GMTR
VQQTVQDLFG R
VQQTVQDLFG R
SQVFSTAADG QTQVEIK
SQVFSTAADG QTQVEIK
EMAGDNKLLG QFTLIGIPPA PR
EMAGDNKLLG QFTLIGIPPA PR
EMAGDNKLLG QFTLIGIPPA PR
EMAGDNKLLG QFTLIGIPPA PR
EMAGDNKLLG QFTLIGIPPA PR
EMAGDNKLLG QFTLIGIPPA PR
EMAGDNKLLG QFTLIGIPPA PR
LLGQFTLIGI PPAPR
LLGQFTLIGI PPAPR
LLGQFTLIGI PPAPR
LLGQFTLIGI PPAPR
LLGQFTLIGI PPAPR
LLGQFTLIGI PPAPR
LLGQFTLIGI PPAPR
LLGQFTLIGI PPAPR
LLGQFTLIGI PPAPR

						VEAVNMAEGI IHDTETK	
						VEAVNMAEGI IHDTETK	
						KDSETGENIR QAASSLQQAS LK	
						QAASSLQQAS LKLFEMAYKK	
						QAASSLQQAS LKLFEMAYKK	
						QAASSLQQAS LKLFEMAYKK	
						QAASSLQQAS LK	
						QAASSLQQAS LK	
PPCKM_HUMAN	Phosphoenolpyruvate carboxykinase [GTP]	8	18	70.6	7.56	VLSGDLGQLP TGIRDFVEHS AR	Mitochondrion
						VLSGDLGQLP TGIRDFVEHS AR	
						LCQPEGIHIC DGTEAENTAT LTLLEQQGLI R	
						LCQPEGIHIC DGTEAENTAT LTLLEQQGLI R	
						GQLGNWMSPA DFQR	
						TMYVLPFSMG PVGSPLSR	
						IGVQLTDSAY VVASMR	
						LGTPVLQALG DGDFVK	
						LGTPVLQALG DGDFVK	
						LGTPVLQALG DGDFVK	
						LGTPVLQALG DGDFVK	
						LGTPVLQALG DGDFVK	
						GVPLVYEAFN WR	
						GVPLVYEAFN WR	
						GVPLVYEAFN WR	
						GVPLVYEAFN WR	
						AIDTTQLFSL PK	
						AIDTTQLFSL PK	
CMC2_HUMAN	Calcium-binding mitochondrial carrier protein Aralar2	7	16	74.1	7.14	YLNIFGESQP NPK	Integral to plasam membrane
						YLNIFGESQP NPK	
						IAPLEEGTLP FNLAEAQR	
						IAPLEEGTLP FNLAEAQR	
						IAPLEEGTLP FNLAEAQR	

						IAPLEEGTLP FNLAEAQR	
						QKASGDSARP VLLQVAESAY R	
						QKASGDSARP VLLQVAESAY R	
						ASGDSARPVL LQVAESAYR	
						ASGDSARPVL LQVAESAYR	
						ASGDSARPVL LQVAESAYR	
						ASGDSARPVL LQVAESAYR	
						FGLGSVAGAV GATAVYPIDL VK	
						FGLGSVAGAV GATAVYPIDL VK	
						GLLPQLLGVA PEK	
						LQVAGEITTG PR	
HSP71_HUMAN	Heat shock 70 kDa protein 1	6	13	70	5.48	TTPSYVAFTD TER	Cytoplasam
						AFYPEEISSM VLTK	
						AFYPEEISSM VLTK	
						NAVITVPAYF NDSQR	
						NAVITVPAYF NDSQR	
						NAVITVPAYF NDSQR	
						NAVITVPAYF NDSQR	
						QATKDAGVIA GLNVLR	
						QATKDAGVIA GLNVLR	
						QATKDAGVIA GLNVLR	
						DAGVIAGLNV LR	
						IINEPTAAAI AYGLDR	
						IINEPTAAAI AYGLDR	
						ELEQVCNPII SGLYQGAGGP GPGGFGAQGP K	
						ELEQVCNPII SGLYQGAGGP GPGGFGAQGP K	
						ELEQVCNPII SGLYQGAGGP GPGGFGAQGP K	
						ELEQVCNPII SGLYQGAGGP GPGGFGAQGP K	
GPDM_HUMAN	Glycerol-3-phosphate dehydrogenase	8	15	80.8	7.23	MNLAIALTAA R	Mitochondrial inner membrane
						YGAATANYME VVSLLK	
						YGAATANYME VVSLLK	
						CKDVLTGQEF DVR	
						SMAEDTINAA VK	

						TVGLFLQGGK DWSPTLYIR	
						LVQDYGLESE VAQHLAATYG DK	
						LVQDYGLESE VAQHLAATYG DK	
						TRLAFLNVQA AEEALPR	
						TRLAFLNVQA AEEALPR	
						TRLAFLNVQA AEEALPR	
						TRLAFLNVQA AEEALPR	
						LAFLNVQAAE EALPR	
						LAFLNVQAAE EALPR	
						LAFLNVQAAE EALPR	
						LAFLNVQAAE EALPR	
						LAFLNVQAAE EALPR	
GRP78_HUMAN	78 kDa glucose-regulated protein	7	12	72.3	5.07	TKPYIQVDIG GGQTK	Integral to endoplasm reticulum
						TFAPEEISAM VLTK	
						TFAPEEISAM VLTK	
						TFAPEEISAM VLTK	
						TFAPEEISAM VLTK	
						VTHAVVTVPA YFNDAQR	
						IEIESFYEGE DFSETLTR	
						IEIESFYEGE DFSETLTR	
						AKFEELNMDL FR	
						AKFEELNMDL FR	
						SQIFSTASDN QPTVTIK	
						DNHLLGTFDL TGIPPAPR	
RIB1_HUMAN	Dolichyl-diphosphooligosaccharide--protein glycosyltransferase subunit 1	5	11	68.5	5.96	VTAEVVLAHL GGGSTSR	Membrane
						VTAEVVLAHL GGGSTSR	
						VTAEVVLAHL GGGSTSR	
						VTAEVVLAHL GGGSTSR	
						ATSFLLALEP ELEAR	

						ATSFLLALEP ELEAR	
						ATSFLLALEP ELEAR	
						ATSFLLALEP ELEAR	
						SEDLLDYGPF R	
						YDYQRQPDSG ISSIR	
						ALTSEIALLQ SR	
ACADV_HUMAN	Very long-chain specific acyl-CoA dehydrogenase	3	5	70.3	8.92	ASNTAEVFFD GVRVPSENVL GEVGSGFK	Mitochondrial inner membrane
						ASNTAEVFFD GVRVPSENVL GEVGSGFK	
						AGLGSGLSLS GLVHPELSR	
						AGLGSGLSLS GLVHPELSR	
						ALVERGGVVT SNPLGF	
ACSL5_HUMAN	Long-chain-fatty-acid--CoA ligase 5	2	4	75.9	6.49	GAMITHQNIV SNAAAFLK	Integral to membrane
						GAMITHQNIV SNAAAFLK	
						SSLVGVVVPD TDVLPSFAAK	
						SSLVGVVVPD TDVLPSFAAK	
ODP2_HUMAN	Dihydrolipoyllysine-residue acetyltransferase component of pyruvate dehydrogenase complex	3	5	65.7	7.96	ILVPEGTRDV PLGTPLCIIV EK	Mitochondrion
						DVPLGTPLCI IVEK	
						GVETIANDVV SLATK	
						GVETIANDVV SLATK	
						GVETIANDVV SLATK	
TRAP1_HUMAN	Heat shock protein 75 kDa	3	6	80.1	8.3	GTITIQDTGI GMTQEELVSN LGTIAR	Mitochondrion
						GTITIQDTGI GMTQEELVSN LGTIAR	
						GTITIQDTGI GMTQEELVSN LGTIAR	
						ATDILPKW	
						AQLLQPTLEI NPR	
						AQLLQPTLEI NPR	
DHSA_HUMAN	Succinate dehydrogenase [ubiquinone] flavoprotein subunit	2	5	72.6	7.06	VSDSISAQYP VVDHEFDAVV VGAGGAGLR	Membrane

						VSDSISAQYP VVDHEFDAVV VGAGGAGLR	
						LGANSLLDLV VFGR	
						LGANSLLDLV VFGR	
						LGANSLLDLV VFGR	
AIFM1_HUMAN	Apoptosis-inducing factor 1	2	3	66.9	9.04	VLIVSEDPEL PYMRPPLSK	Mitochondrion
						ALGTEVIQLF PEKGNMGK	
						ALGTEVIQLF PEKGNMGK	
ECHA_HUMAN	Trifunctional enzyme subunit alpha	17	46	82.9	9.16	ELHSEFSEVM NEIWASDQIR	Mitochondrial inner membrane.
						TVLGTPEVLL GALPGAGGTQ R	
						TVLGTPEVLL GALPGAGGTQ R	
						TVLGTPEVLL GALPGAGGTQ R	
						TVLGTPEVLL GALPGAGGTQ R	
						TVLGTPEVLL GALPGAGGTQ R	
						TVLGTPEVLL GALPGAGGTQ R	
						MVGVPAALDM MLTGR	
						MVGVPAALDM MLTGR	
						MVGVPAALDM MLTGR	
						MVGVPAALDM MLTGR	
						MVGVPAALDM MLTGR	
						MVGVPAALDM MLTGR	
						MVGVPAALDM MLTGR	
						MVGVPAALDM MLTGR	
						MGLVDQLVEP LGPGLKPPEE R	
						MGLVDQLVEP LGPGLKPPEE R	
						MGLVDQLVEP LGPGLKPPEE R	
						TIEYLEEVAI TFAK	
						TIEYLEEVAI TFAK	
						TIEYLEEVAI TFAK	
						TIEYLEEVAI TFAK	
						LTAYAMTIPF VR	
						LTAYAMTIPF VR	
						LTAYAMTIPF VR	
						DSIFSNLTGQ LDYQGFEK	

						DSIFSNLTGQ LDYQGFEK	
						ADMVIEAVFE DLSLK	
						ADMVIEAVFE DLSLK	
						ADMVIEAVFE DLSLK	
						VLKEVEAVIP DHCIFASNTS ALPISEIAAV SK	
						VLKEVEAVIP DHCIFASNTS ALPISEIAAV SK	
						EVEAVIPDHC IFASNTSALP ISEIAAVSK	
						EVEAVIPDHC IFASNTSALP ISEIAAVSK	
						MQLLEIITTE K	
						MQLLEIITTE K	
						CLAPMMSEVI R	
						KLDSLTTSFG FPVGAATLVD EVGVDVAK	
						KLDSLTTSFG FPVGAATLVD EVGVDVAK	
						KLDSLTTSFG FPVGAATLVD EVGVDVAK	
						HVAEDLGKVF GER	
						FGGGNPELLT QMVSK	
						FGGGNPELLT QMVSK	
						DLNSDMDSIL ASLK	
						DLNSDMDSIL ASLK	
						DLNSDMDSIL ASLK	
						DLNSDMDSIL ASLK	
						QFTPCQLLAD HANSPNKK	
NUCL_HUMAN	Nucleolin	6	13	76.6	4.6	QKVEGTEPTT AFNLFVGNLN FNK	Cell cortex
						QKVEGTEPTT AFNLFVGNLN FNK	
						VEGTEPTTAF NLFVGNLNFN K	
						VEGTEPTTAF NLFVGNLNFN K	
						VEGTEPTTAF NLFVGNLNFN K	
						VEGTEPTTAF NLFVGNLNFN K	
						TGISDVFAKN DLAVVDVR	
						NLPYKVTQDE LKEVFEDAAE IR	
						NLPYKVTQDE LKEVFEDAAE IR	
						TLVLSNLSYS ATEETLQEVF EK	
						TLVLSNLSYS ATEETLQEVF EK	
						TLVLSNLSYS ATEETLQEVF EK	

						GLSEDTTEET LKESFDGSVR	
CALX_HUMAN	Calnexin	5	9	67.5	4.47	APVPTGEVYF ADSFDR	Integral to membrane
						APVPTGEVYF ADSFDR	
						GTLSGWILSK	
						GTLSGWILSK	
						CESAPGCGVW QRPVIDNPNY KGK	
						KIPNPDFFED LEPFR	
						KIPNPDFFED LEPFR	
						IPNPDFFEDL EPFR	
						IPNPDFFEDL EPFR	
ACON_HUMAN	Aconitate hydratase	2	3	85.4	7.36	AKDINQEVYN FLATAGAK	Mitochondrion
						NDANPETHAF VTSPEIVTAL AIAGTLK	
						NDANPETHAF VTSPEIVTAL AIAGTLK	
IMMT_HUMAN	Mitochondrial inner membrane protein	2	2	83.6	6.08	LAQQEKQEQV KIESLAK	Integral to membrane
						QTASVTLQAI AAQNAAVQAV NAHSNILK	
						QTASVTLQAI AAQNAAVQAV NAHSNILK	
PDIA4_HUMAN	Protein disulfide-isomerase A4	2	2	72.9	4.96	EVSQPDWTPP PEVTLVLTK	Endoplasmic reticulum (lumen)
						YGIVDYMIEQ SGPPSKEILT LK	
ENPL_HUMAN	Endoplasmin	16	28	92.4	4.76	EGSRTDDEVV QREEEAIQLD GLNASQIR	Endoplasm reticulum membrane
						EGSRTDDEVV QREEEAIQLD GLNASQIR	
						TDDEVVQREE EAIQLDGLNA SQIR	
						TDDEVVQREE EAIQLDGLNA SQIR	
						ELISNASDAL DKIR	
						ELISNASDAL DKIR	
						LISLTDENAL SGNEELTVK	
						LISLTDENAL SGNEELTVK	
						LISLTDENAL SGNEELTVK	
						LISLTDENAL SGNEELTVK	
						NLLHVTDTGV GMTREELVK	

						SILFVPTSAP R	
						GLFDEYGSK	
						RVFITDDFHD MMPK	
						GVVDSDDLPL NVSR	
						GVVDSDDLPL NVSR	
						FQSSHHPTDI TSLDQYVER	
						FQSSHHPTDI TSLDQYVER	
						GYEVIYLTEP VDEYCIQALP EFDGKR	
						GYEVIYLTEP VDEYCIQALP EFDGKR	
						EAVEKEFEPL LNWMKDK 625alkd	
						EAVEKEFEPL LNWMKDK	
						EAVEKEFEPL LNWMK	
						LTESPCALVA SQYGWSGNME R	
						LTESPCALVA SQYGWSGNME R	
						AQAYQTGKDI STNYYASQK	
						SGYLLPDTK	
						SGYLLPDTK	
ACTN4_HUMAN	Alpha-actinin-4	12	27	104.8	5.27	KDDPVTNLNN AFEVAEK	Extracellular region
						KDDPVTNLNN AFEVAEK	
						KDDPVTNLNN AFEVAEK	
						LASDLLEWIR	
						LASDLLEWIR	
						CQLEINFNTL QTK	
						DYETATLSDI KALIR	
						QLEAIDQLHL EYAK	
						QLEAIDQLHL EYAK	
						QLEAIDQLHL EYAK	
						LSGSNPYTTV TPQIINSKWE K	
						LSGSNPYTTV TPQIINSKWE K	
						QFASQANVVG PWIQTK	
						QFASQANVVG PWIQTK	
						QFASQANVVG PWIQTK	
						QFASQANVVG PWIQTK	

						QFASQANVVG PWIQTK	
						VGWEQLLTTI AR	
						VGWEQLLTTI AR	
						VGWEQLLTTI AR	
						VGWEQLLTTI AR	
						TINEVENQIL TR 7	
						TINEVENQIL TR	
						GISQEQMQEF R	
						ETTDTDTADQ VIASFK	
						MAPYQGPDAV PGALDYKSFS TALYGESDL	
						MAPYQGPDAV PGALDYKSFS TALYGESDL	
ODO1_HUMAN	2-oxoglutarate dehydrogenase E1 component	8	18	115.9	6.39	FGLEGCEVLI PALK	Mitochondrial membrane
						FGLEGCEVLI PALK	
						FGLEGCEVLI PALK	
						FGLEGCEVLI PALK	
						VVNAPIFHVN SDDPEAVMYV CK	
						VVNAPIFHVN SDDPEAVMYV CK	
						STFHKDVVVD LVCYR	
						YAELLVSQGV VNQPEYEEEI SKYDK	
						QILLPFRKPL IIFTPK	
						QILLPFRKPL IIFTPK	
						IEQLSPFPFD LLLK	
						IEQLSPFPFD LLLK	
						IEQLSPFPFD LLLK	
						IEQLSPFPFD LLLK	
						LLDTAFDLDV FKNFS	
						LLDTAFDLDV FK	
						LLDTAFDLDV FK	
						LLDTAFDLDV FK	
GANAB_HUMAN	Neutral alpha-glucosidase AB	6	9	106.8	5.74	SLLLSVNAR	Endoplasmic reticulum
						QYASLTGTQA LPPLFSLGYH QSR	

SYIM_HUMAN	Isoleucyl-tRNA synthetase	5	7	113.7	6.78	HHGPQTLYLP VTLSSIPVFQ R<br>FSFSGNTLVS SSADPEGHFE TPIWIER<br>FSFSGNTLVS SSADPEGHFE TPIWIER<br>VVIIGAGKPA AVVLQTK<br>VVIIGAGKPA AVVLQTK<br>LSFQHDPETS VLVLR<br>LSFQHDPETS VLVLR<br>TALAEAELEY NPEHVSR<br>TALAEAELEY NPEHVSR<br>AVLEEGTDVV IK<br>TKDEYLINSQ TTEHIVK<br>TVIVHGFTLG EKGEKMSKSL<br>SCQTALVEIL DVIVR<br>SCQTALVEIL DVIVR	Mitochondrion
TERA_HUMAN	Transitional endoplasmic reticulum ATPase	4	6	89.3	5.14	GILLYGPPGT GK	Endoplasmic reticulum
LONM_HUMAN	Lon protease homolog	4	7	106.4	6.01	QAAPCVLFFD ELDSIAK<br>QAAPCVLFFD ELDSIAK<br>QAAPCVLFFD ELDSIAK<br>LDQLIYIPLP DEK<br>KSPVAKDVDL EFLAK<br>ILEFIAVSQL R<br>ILEFIAVSQL R<br>VLFICTANVT DTIPEPLRDR<br>LSSDVLTLLI K<br>LSSDVLTLLI K<br>IVSGEAESVE VTPENLQDFV GKPVFTVER<br>IVSGEAESVE VTPENLQDFV GKPVFTVER<br>IVSGEAESVE VTPENLQDFV GKPVFTVER<br>IVSGEAESVE VTPENLQDFV GKPVFTVER	Mitochondrion
SND1_HUMAN	Staphylococcal nuclease domain-containing protein 1	2	2	101.9	6.74	NLPGLVQEGE PFSEEATLFT K<br>VLPAQATEYA FAFIQVPQDD DARTDAVDSV VR	Cytoplasm

AT1A1_HUMAN	Sodium/potassium-transporting ATPase subunit alpha-1 precursor	2	2	112.8	5.13	GVGIISEGNE TVEDIAAR	Integral to membrane
						LNIPVSQVNP R	
ITB1_HUMAN	Integrin beta-1; Fibronectin receptor subunit beta	3	4	88.4	5.3	IGFGSFVEKT VMPYISTTPA K	Plasma membrane
						IGFGSFVEK	
						IGFGSFVEK	
						SAVTTVVNPK YEGK	
						SAVTTVVNPK YEGK	
HNRL2_HUMAN	Heterogeneous nuclear ribonucleoprotein U-like protein 2	2	3	85.1	4.85	YGGQPLFSEK FPTLWSGAR	Nucleus
						YNVLGAETVL NQMR	
						YNVLGAETVL NQMR	
IMB1_HUMAN	Importin subunit beta-1	3	5	97.1	4.68	MELITILEK	Nucleus envelope
						MELITILEK	
						NEILTAIIQG MRKEEPSNNV KLAATNALLN	
						SLEFTKANFD KESER	
						LAATNALLNS LEFTK	
						LAATNALLNS LEFTK	
HNRPU_HUMAN	Heterogeneous nuclear ribonucleoprotein U	2	8	90.5	5.76	SSGPTSLFAV TVAPPGAR	Cell surface
						SSGPTSLFAV TVAPPGAR	
						SSGPTSLFAV TVAPPGAR	
						SSGPTSLFAV TVAPPGAR	
						SSGPTSLFAV TVAPPGAR	
						SSGPTSLFAV TVAPPGAR	
						EKPYFPIPEE YTFIQNVPLE DR	
						EKPYFPIPEE YTFIQNVPLE DR	
S12A1_HUMAN	Solute carrier family 12 member 1	2	2	121.3	7.18	YLGVAICVGA CVVRDATGNM NDTIISGMNC NGSAACGLGY DFSR	Integral to membrane
						ARKGSISDLL YMAWLEILTK NLPPVLLVRG NHKNVLTFYS	

IL4RA_HUMAN	Interleukin-4 receptor alpha chain	2	2	89.6	4.99	MGWLCSGLLF PVSCLVLLQV ASSGNMKVLQ EPTCVSDYMS ISTCEW<br>MGWLCSGLLF PVSCLVLLQV ASSGNMKVLQ EPTCVSDYMS ISTCEW<br>MGWLCSGLLF PVSCLVLLQV ASSGNMKVLQ EPTCVSDYMS ISTCEW<br>MGWLCSGLLF PVSCLVLLQV ASSGNMKVLQ EPTCVSDYMS ISTCEW<br>MGWLCSGLLF PVSCLVLLQV ASSGNMKVLQ EPTCVSDYMS ISTCEW<br>MGWLCSGLLF PVSCLVLLQV ASSGNMKVLQ EPTCVSDYMS ISTCEW<br>MGWLCSGLLF PVSCLVLLQV ASSGNMKVLQ EPTCVSDYMS ISTCEW<br>MGWLCSGLLF PVSCLVLLQV ASSGNMKVLQ EPTCVSDYMS ISTCEW<br>APDPSPGGVP LEAS	Integral to plasma membrane
FRAP_HUMAN	FKBP12-rapamycin complex-associated protein	2	2	288.7	6.73	DASAVSLSES KSSQDSSDYS TSE<br>SGQGDALASG PVETGPMKK	Golgi membrane
LPPRC_HUMAN	Leucine-rich PPR motif-containing protein	15	26	157.8	5.81	AIAAKEKDIQ EESTFSSR<br>LIASYCNVGD IEGASKILGF MK<br>LIASYCNVGD IEGASK<br>LIASYCNVGD IEGASK<br>TKDLPVTEAV FSALVTGHAR<br>TKDLPVTEAV FSALVTGHAR<br>DLPVTEAVFS ALVTGHAR<br>DLPVTEAVFS ALVTGHAR<br>DLPVTEAVFS ALVTGHAR<br>DLPVTEAVFS ALVTGHAR<br>FSALVTGHAR<br>AGDMENAENI LTVMR<br>AGDMENAENI LTVMR	Membrane

						SNTLPISLQS IR	
						TVQLTSSELE STLETLK	
						TVQLTSSELE STLETLK	
						QLILVLCSEE NMQK	
						CVANNQVETL EKLVELTQK	
						CVANNQVETL EKLVELTQK	
						AFAETHIKGF TLNDAANSR	
						RDYLKEAVTT LK	
						MVFINNIALA QIK	
						SVLELIPELN EKEEAYNSLM K	
						SVLELIPELN EKEEAYNSLM K	
						SVLELIPELN EKEEAYNSLM K	
						SVLELIPELN EKEEAYNSLM K	
						SVLELIPELN EKEEAYNSLM K	
FA62A_HUMAN	Extended synaptotagmin-1	3	5	122.8	5.57	ALTLGALTLP LAR	Membrane
						ALTLGALTLP LAR	
						LTPRPTAAEL EEVLQVNSLI QTQK	
						LTPRPTAAEL EEVLQVNSLI QTQK	
						HLSPYATLTV GDSSHK	
HYOU1_HUMAN	Hypoxia up-regulated protein 1	3	5	111.3	5.16	DINTTAQNIM FYDMGSGSTV CTIVTYQMVK TKEAGMQPQL	Endoplasmic reticulum
						DAVVYPILVE FTR	
						DAVVYPILVE FTR	
						DAVVYPILVE FTR	
						LQDLTLRDLE KQER	
KTN1_HUMAN	Kinectin	2	2	156.2	5.52	SVEELLEAEL LK	Integral to plasma membrane
						ALKEEIGNVQ LEK	
EDC4_HUMAN	Enhancer of mRNA-decapping protein 4	2	2	151.6	5.55	ASIDIEDATQ HLR	Cytoplasm
						ALQDVQIRFQ PQLNPDVVAP LPTHTAHEDF TFGESRPELG	
						ALQDVQIRFQ PQLNPDVVAP LPTHTAHEDF TFGESRPELG	

CLH1_HUMAN	Clathrin heavy chain 1	7	10	191.5	5.48	ISGETIFVTA PHEATAGIIG VNR<br>GQCDLELINV CNENSLFK<br>NLQNLLILTA IK<br>NLQNLLILTA IK<br>ESYVETELIF ALAK<br>ESYVETELIF ALAK<br>LLYNNVSNFG R<br>LASTLVHLGE YQAAVDGAR<br>LASTLVHLGE YQAAVDGAR<br>QLPLVKPYLR	Plasma membrane
MYOF_HUMAN	Myoferlin	4	5	234.6	5.84	ILVELATFLE K<br>ILVELATFLE K<br>IPANQLAELW LK<br>YPQEKNNGPK VPVELRVNIW LGLSAVEKKF NSFAEG<br>ACGDVLVTAE LILR	Integral to membrane
ITB4_HUMAN	Integrin beta-4	2	3	202	5.74	CSFNGDFVCG QCVCSEGWSG QTCNCSTGSL SDIQPCLREG ED<br>CSFNGDFVCG QCVCSEGWSG QTCNCSTGSL SDIQPCLREG ED<br>LLELQEVDSL LR<br>LLELQEVDSL LR	Plasma membrane
IQGA1_HUMAN	Ras GTPase-activating-like protein IQGAP1	2	2	189.1	6.08	ILAIGLINEA LDEGDAQK<br>FQPGETLTEI LETPATSEQE AEHQR	Plasma membrane
SC16A_HUMAN	Protein transport protein Sec16A	2	2	233.4	5.4	CESPATTLWA QSELPDFGGN VLLAPAAPAL YVCAKPQPPV VQPPEEAM<br>AGSALPGFAN SPAGSTSVVL VPPAHGTLVP DGNKANHSSH QEDT	Membrane
FLNB_HUMAN	Filamin-B	2	3	278	5.49	SPFTVGVAAP LDLSK<br>VTASGPGLSS YGVPASLPVD FAIDAR<br>VTASGPGLSS YGVPASLPVD FAIDAR	Integral to membrane

PLEC1_HUMAN	Plectin-1	3	4	531.4	5.73	QVKLVNIRND DIADGNPKLT LGLIWTIIL<br>QVKLVNIRND DIADGNPKLT LGLIWTIIL<br>ALQALEELRL QAEEAER<br>APVPASELLA SGVLSR<br>APVPASELLA SGVLSR	Plasam membrane
ZFHX4_HUMAN	Zinc finger homeobox protein 4	2	2	393.5	5.94	LENPADPQLM INPFQLDPAT AAALAPGLGE LSPY<br>CSICNVAYSQ SSTLEIHMRS VLHQTKARAA KLEPSGHV	Nucleus
PRKDC_HUMAN	DNA-dependent protein kinase catalytic subunit	2	2	468	6.75	CGAALAGHQL IR<br>CELLHSMVMF MLGKATQMPE	Nucleus
4F2_HUMAN	4F2 cell-surface antigen heavy chain	2	2	57.9	5.2	LLTSFLPAQL LR<br>GQSEDPGSLL SLFR	Integral to membrane
SRP68_HUMAN	Signal recognition particle 68 kDa protein	2	2	70.7	8.75	MAAEKQVPGG GGGGGSGGGG G<br>QVPGGGGGGG SGGGGGSGGG GS	Endoplasmic reticulum
OST48_HUMAN	Dolichyl-diphosphooligosaccharide--protein glycosyltransferase 48 kDa subunit	2	2	48.8	5.43	TLVLLDNLNV R	Integral to membrane
ZZEF1_HUMAN	Zinc finger ZZ-type and EF-hand domain-containing protein 1	2	2	330.8	5.62	VGETAPPNAY TVTDLVEYSI VIQQLSNGK<br>CSDMDLCKTC FLGGVKPEGH GDDHEMVNME FTCDHCQGLI IGR	Anaphase-promoting complex
ARI1A_HUMAN	AT-rich interactive domain-containing protein 1A	2	2	241.9	6.24	QPSKSLRLEE QSAKAVDTDM IILPCLSRPA RCDQATAESN PVTQK<br>TPQPSSPMDQ MGKMRPQPYG GTNPYSQQQG PPSGPQQGHG YPGQPYG	Nucleus
IF4G1_HUMAN	Eukaryotic translation initiation factor 4 gamma 1	2	2	175.4	5.27	MRPQPYGGTN PYSQQQGPPS GPQQGHGYPG<br>VQSAAPARPG PAAHVYPAGS QVMMIPSQIS YPASQGAYYI PGQGR	Cytosol

ANPRC_HUMAN	Atrial natriuretic peptide clearance receptor	2	2	59.8	5.93	EFLLGFQFIF ASMQKPEGLP HISDVVLDKA NKTPLRPLDP TRLQGI<br>LAGGTGGGGV GGGGGGAGIG GGR<br>GGTGGGGVGG GGGGAGIGGG RQER	Integral to membrane
VP13D_HUMAN	Vacuolar protein sorting-associated protein 13D	2	2	491.5	6.15	SIPEQANAAV PDSVALESDS VGTYLPGASR VGE	
LAMP1_HUMAN	Lysosome-associated membrane glycoprotein 1	2	3	44.7	9	IGGLTSVITS TVEGVKTEGG VSGFISGLGK<br>TVESITDIRA DIDKK<br>FFLQGIQLNT ILPDARDPAF K	Integral to plasma membrane
HS90B_HUMAN	Heat shock protein HSP 90-beta	3	3	83.2	4.97	FFLQGIQLNT ILPDARDPAF K<br>ADLINNLGTI AK<br>GVVDSEDLPL NISR	Cytoplasm
CEAM6_HUMAN	Carcinoembryonic antigen-related cell adhesion molecule 6	3	4	37.2	5.56	CLELFSELAE DKENYKK<br>HLEINPDHPI VETLR<br>EVLLLAHNLP QNR<br>IGYSWYKGER<br>VDGNSLIVGY VIGTQQATPG PAYSGR	Integral to plasma membrane
P5CS_HUMAN	Delta-1-pyrroline-5-carboxylate synthetase	2	4	87.2	6.66	VDGNSLIVGY VIGTQQATPG PAYSGR<br>VDGNSLIVGY VIGTQQATPG PAYSGR<br>SNIPFITVPL SR	Mitochondrial inner membrane
VMAT2_HUMAN	Synaptic vesicular amine transporter	2	2	55.7	5.69	GPVGLEGLLT TK<br>GPVGLEGLLT TK<br>GTPLTTLLKD PYILIAAGSI CFANMGIAML EPALPIWMME TM<br>GTPLTTLLKD PYILIAAGSI CFANMGIAML EPALPIWMME TM<br>GTPLTTLLKD PYILIAAGSI CFANMGIAML EPALPIWMME TM	Integral to plasma membrane

						IPFAKNIYGL IAPNFGVGFA IGMVDSSMMP IMGYLVDLR	
						IPFAKNIYGL IAPNFGVGFA IGMVDSSMMP IMGYLVDLR	
AOFB_HUMAN	Amine oxidase [flavin-containing] B	2	2	58.7	7.2	CIVYYKEPFW R	Integral to membrane
						IPEDEIWQSE PESVDVPAQP ITTTFLER	
HNRH1_HUMAN	Heterogeneous nuclear ribonucleoprotein H	2	4	49.2	5.89	STGEAFVQFA SQEIAEK	Nucleus
						STGEAFVQFA SQEIAEK	
						ATENDIYNFF SPLNPVR	
						ATENDIYNFF SPLNPVR	
TBA1A_HUMAN	Tubulin alpha-1A chain	2	3	50.1	4.94	AVFVDLEPTV IDEVR	Microtubule
						AVFVDLEPTV IDEVR	
						QLFHPEQLIT GKEDAANNYA R	
VIME_HUMAN	Vimentin	2	4	53.6	5.06	LLQDSVDFSL ADAINTEFK	Plasma membrane
						ISLPLPNFSS LNLR	
						ISLPLPNFSS LNLR	
						ISLPLPNFSS LNLR	
ACTG_HUMAN	Actin, cytoplasmic 2	14	28	41.8	5.31	EEEIAALVID NGSGMCK	Cytoplasm
						EEEIAALVID NGSGMCK	
						EEEIAALVID NGSGMCK	
						AGFAGDDAPR	
						AGFAGDDAPR	
						AGFAGDDAPR	
						AVFPSIVGRP R	
						AVFPSIVGRP R	
						IWHHTFYNEL R	
						IWHHTFYNEL R	
						VAPEEHPVLL TEAPLNPK	
						VAPEEHPVLL TEAPLNPK	
						VAPEEHPVLL TEAPLNPK	
						TTGIVMDSGD GVTHTVPIYE GYALPHAILR	
						TTGIVMDSGD GVTHTVPIYE GYALPHAILR	

						DLTDYLMK	
						GYSFTTTAER	
						EKLCYVALDF EQEMATAASS SSLEK	
						LCYVALDFEQ EMATAASSSS LEK	
						LCYVALDFEQ EMATAASSSS LEK	
						LCYVALDFEQ EMATAASSSS LEK	
						LCYVALDFEQ EMATAASSSS LEK	
						SYELPDGQVI TIGNER	
						SYELPDGQVI TIGNER	
						CPEALFQPSF LGMESCGIHE TTFNSIMK	
						DLYANTVLSG GTTMYPGIAD R	
						DLYANTVLSG GTTMYPGIAD R	
						EITALAPSTM K	
AATM_HUMAN	Aspartate aminotransferase	4	6	47.4	9.14	SSWWTHVEMG PPDPILGVTE AFK	Plasma membrane
						NLDKEYLPIG GLAEFCK	
						NLDKEYLPIG GLAEFCK	
						FVTVQTISGT GALR	
						TCGFDFTGAV EDISK	
						TCGFDFTGAV EDISK	
THIK_HUMAN	3-ketoacyl-CoA thiolase	3	4	44.3	8.76	GGFKDTTPDE LLSAVMTAVL K	Intracecellular membrane-bounded organelle
						GAGAIMARIA QFLSDIPETV PLSTVNR	
						AEELGLPILG VLR	
						AEELGLPILG VLR	
ODPA_HUMAN	Pyruvate dehydrogenase E1 component alpha subunit, somatic form	2	4	43.3	8.35	LEEGPPVTTV LTR	Intracecellular membrane-bounded organelle
						EIEDAAQFAT ADPEPPLEEL GYHIYSSDPP FEVR	
						EIEDAAQFAT ADPEPPLEEL GYHIYSSDPP FEVR	
						EIEDAAQFAT ADPEPPLEEL GYHIYSSDPP FEVR	

TMLH_HUMAN	Trimethyllysine dioxygenase	2	3	49.5	7.64	TLLVDGFYAA EQVLQK	Mitochondrion matrix
						TLLVDGFYAA EQVLQK	
						EDVGECHNHM IGIGPVLNIY PWNK	
DYH9_HUMAN	Dynein heavy chain 9, axonemal	2	2	511.6	5.65	NLNTVFLMTD AQVADERFLV LINDLLASGE	Dynein complex
						IPDLYSDDEV ENII	
						VTQIGQKGYL QIIEQALEAG AVVLIENLEE	
						SIDPVLGPLL GREVIKKG	
SFRS1_HUMAN	Splicing factor, arginine/serine-rich 1	2	6	27.7	10.37	GGPPFAFVEF EDPRDAEDAV YGR	Cytosome
						GGGGGGGGGA PR	
						GGGGGGGGGA PR	
						GGGGGGGGGA PR	
						GGGGGGGGGA PR	
						GGGGGGGGGA PR	
1433Z_HUMAN	14-3-3 protein zeta/delta	2	3	27.7	4.73	SVTEQGAELS NEERNLLSVA YK	Cytoplasm
						SVTEQGAELS NEERNLLSVA YK	
						DICNDVLSLL EK	
						DICNDVLSLL EK	
CY1_HUMAN	Cytochrome c1, heme protein	2	3	35.4	9.15	SDLELHPPSY PWSHR	Integral to membrane
						AANNGALPPD LSYIVR	
						AANNGALPPD LSYIVR	
RAB1A_HUMAN	Ras-related protein Rab-1A	3	7	22.7	5.93	VVDYTTAKEF ADSLGIPFLE TSAK	Endoplasmic reticulum.
						VVDYTTAKEF ADSLGIPFLE TSAK	
						EFADSLGIPF LETSAK	
						EFADSLGIPF LETSAK	
						EFADSLGIPF LETSAK	
						EFADSLGIPF LETSAK	
						NATNVEQSFM TMAAEIK	
SODM_HUMAN	Superoxide dismutase [Mn]	2	5	24.7	8.35	DFGSFDKFK	Mitochondrion
						AIWNVINWEN VTER	
						AIWNVINWEN VTER	

						AIWNVINWEN VTER	
						AIWNVINWEN VTER	
RHOA_HUMAN	Transforming protein RhoA precursor	2	3	21.8	5.83	QVELALWDTA GQEDYDR	Plasma membrane
						QVELALWDTA GQEDYDR	
						HFCPNVPIIL VGNKK	
PPRC1_HUMAN	Peroxisome proliferator-activated receptor gamma coactivator-related protein 1	2	2	177.4	6.11	SSSSSSSSSS SSSSSSSSR	Nucleus
						SSSSSSSSSS SSSRSR	
LYPA1_HUMAN	Acyl-protein thioesterase 1	2	2	24.7	6.29	IILGGFSQGG ALSLYTALTT QQK	Mitochondrion
						LAGVTALSCW LPLR	
TEP1_HUMAN	Telomerase protein component 1	2	2	290.2	8.26	ETLEQSQGAH VLALGPLEAS ARAR	Nucleus
						DVKLWERPSM QLLGLFRCEG SVSC	
TITIN_HUMAN	Titin	16	16	3813.8	6.01	SIATVEMVID GAAGQQ	Cytosol
						LYVEPAAPLG APTY	
						MAHEGALTGV TTDQK	
						LTVKDVTKED QGEY	
						NDGGMEENMA TLM	
						NDGGMEENMA TLM	
						SNCTVSVHVS DRIVPPSFIR K	
						EIRPGGNYKM	
						GGDPIPNVK	
						FMSPLEDQTV K	
						CGPGEPAYVD EPVNMSTPAT	
						AENAAGISEP SRAT	
						EGAFYKFRVS AVNIAGIGEP GEV	
						MKVQNLLPDH EYQFRVKAE	
						VAAVNEKGRS DPRQLGVPV	
						VLACNAGGPG EPAEVPGTVK	
						YGVSGSDQTL TIKQA	
EGFR_HUMAN	Epidermal growth factor receptor precursor	2	3	134.2	6.26	MRPSGTAGAA LLALLAA	Integral to membrane

DPP4_HUMAN	Dipeptidyl peptidase 4	2	3	88.2	5.67	IPLENLQIIR<br>IPLENLQIIR<br>NTDSLSSVTN ATSIQITAP<br>FRPSEPHFTL DGNSFYK<br>FRPSEPHFTL DGNSFYK	Plasma membrane
NSUN2_HUMAN	tRNA (cytosine-5-)-methyltransferase NSUN2	2	2	86.4	6.33	ELPGLKWMPG ITQWK	Cytoplasm
EF2_HUMAN	Elongation factor 2	2	2	95.3	6.41	ILLTQENPFF R<br>TFCQLILDPI FK<br>GGGQIIPTAR R	Cytoplasm
DHX9_HUMAN	ATP-dependent RNA helicase A	2	2	140.9	6.41	YPSPFFVFGE K	Cytoplasm.
SYG_HUMAN	Glycyl-tRNA synthetase	2	2	83.1	6.61	RAGYGAGVGG GYR<br>LPFAAAQIGN SFR<br>TFFSFPAVVA PFK	Mitochondrial matrix
DDX17_HUMAN	Probable ATP-dependent RNA helicase DDX17	3	3	72.3	3.94	RGGFGARGGG GLPPKK<br>PKLMQLVDHR GGGGGGGGRS R<br>GGGGGGGGRS R	Nucleus
KU70_HUMAN	ATP-dependent DNA helicase 2 subunit 1	3	4	69.8	6.23	IISSDRDLLA VVFYGTEK<br>NIYVLQELDN PGAK<br>DTGIFLDLMH LK<br>DTGIFLDLMH LK	Membrane fraction
HS90A_HUMAN	Heat shock protein HSP 90-alpha	2	3	84.6	4.94	ADLINNLGTI AK<br>GVVDSEDLPL NISR<br>GVVDSEDLPL NISR<br>HLEINPDHSI IETLR<br>HLEINPDHSI IETLR	Cytoplasm
AN32B_HUMAN	Acidic leucine-rich nuclear phosphoprotein 32 family member B	2	2	28.8	3.94	LEFLSLINVG LISVSNL	Nucleus

						LPNLTHLNLS GNK	
APOE_HUMAN	Apolipoprotein E	2	3	36.1	5.65	EGAERGLSAI R	Secreted
						EGAERGLSAI R	
						MEEMGSRTRD RL	
HNRPG_HUMAN	Heterogeneous nuclear ribonucleoprotein G	2	2	42.3	10.06	DMNGKSLDGK AIKVEQATKP	Nucleus
						GGRGGSGGTR	
LEG4_HUMAN	Galectin-4	2	2	35.9	9.21	VGSSGDIALH INPR	Plasma membrane
						ITHNPFGPGQ FFDLSIR	
LRC59_HUMAN	Leucine-rich repeat-containing protein 59	2	4	34.9	9.61	LQQLPADF	Integral to membrane
						LVTLPVSFAQ LK	
						LVTLPVSFAQ LK	
						LVTLPVSFAQ LK	
LEG3_HUMAN	Galectin-3	2	2	26.2	8.58	QSVFPFESGK PFK	Plasma membrane
						IQVLVEPDHF K	
FAM3C_HUMAN	Protein FAM3C precursor	2	3	24.7	8.52	ICLEDNVLMS GVK	Intracecellular region
						LIADLGSTSI TNLGFR	
						LIADLGSTSI TNLGFR	
ROA1_HUMAN	Heterogeneous nuclear ribonucleoprotein A1	2	3	38.8	9.26	LFIGGLSFET TDESLR	Cytoplasm.
						LFIGGLSFET TDESLR	
						IEVIEIMTDR	
HNRPC_HUMAN	Heterogeneous nuclear ribonucleoproteins C1/C2	4	4	33.6	4.95	VFIGNLNTLV VK	Ribonucleosomes.
						GFAFVQYVNE R	
						QKVDSLLENL EK	
						MESEGGADDS AEE	
HNRPD_HUMAN	Heterogeneous nuclear ribonucleoprotein D0	3	9	38.4	7.61	FGEVVDCTLK	Nucleus
						FGEVVDCTLK	
						GFGFVLFK	
						GFGFVLFK	

						GFGFVLFK	
						GFGFVLFK	
						GFGFVLFK	
						IFVGGLSPDT PEEK	
						IFVGGLSPDT PEEK	
H2A1A_HUMAN	Histone H2A type 1-A	2	3	14.2	10.86	AGLQFPVGR	Nucleus
						AGLQFPVGR	
						LLGGVTIAQG GVLPNIQAV	
						LLGGVTIAQG GVLPNIQAV	
						LLGGVTIAQG GVLPNIQAV	
SOCS7_HUMAN	Suppressor of cytokine signaling 7	2	3	62.9	8.35	DVEAAPEPGP SELLCPR	Plasma membrane
						TKSCNGGSGG GDGTGK	
						TKSCNGGSGG GDGTGK	
ATPK_HUMAN	ATP synthase subunit F	2	2	10.9	9.7	DFSPSGIFGA FQR	Membrane
						GSISGITMVL	
H4_HUMAN	Histone H4	2	2	11.4	11.36	LARRGGVKRI SGL	Nucleus
						TVTAMDVVYA LK	
ILF2_HUMAN	Interleukin enhancer-binding factor 2	2	3	43	5.19	ILPTLEAVAA LGNK	Cytoplasm.
						ILPTLEAVAA LGNK	
						GLFLPGSVGI TDPCESGNFR	
RAB10_HUMAN	Ras-related protein Rab-10	2	6	22.5	8.58	LLLIGDSGVG K	plasma membrane
						LLLIGDSGVG K	
						AFLTLAEDIL R	
						AFLTLAEDIL R	
						AFLTLAEDIL R	
						AFLTLAEDIL R	
RAB2A_HUMAN	Ras-related protein Rab-2A	3	3	23.5	6.08	YIIIGDTGVG K	Membrane
						TASNVEEAFI NTAK	
						IGPQHAATNA THAGNQGGQQ AGGGCC	
ROAA_HUMAN	Heterogeneous nuclear ribonucleoprotein A/B	2	3	36.2	8.21	GWTGAAAGAG GATAAP	Nucleus
						GWTGAAAGAG GATAAP	

						FGEVVDCTIK	
						GNRGSGGGGG GGGQSQSW	
ODPAT_HUMAN	Pyruvate dehydrogenase E1 component subunit alpha, testis-specific form	3	7	42.9	8.76	LEEGPPVTTV LTR	Intracecellular membrane-bounded organelle
						LEEGPPVTTV LTR	
						LEEGPPVTTV LTR	
						LEEGPPVTTV LTR	
						QGPLGAGIAL ACK	
						GPILMELQTY R	
						GPILMELQTY R	
PGK1_HUMAN	Phosphoglycerate kinase 1	4	7	44.6	8.3	YSLEPVAVEL K	Cytoplasm
						YSLEPVAVEL K	
						ACANPAAGSV ILLENLR	
						ALESPERPFL AILGGAK	
						ALESPERPFL AILGGAK	
						ALESPERPFL AILGGAK	
						TGQATVASGI PAGW 311mgld	
ABHD8_HUMAN	Abhydrolase domain-containing protein 8	2	3	47.3	6.54	GSGSGSGSGG R	
						GSGSGSGSGG R	
						SGSGSGSGGR	
CEAM1_HUMAN	Carcinoembryonic antigen-related cell adhesion molecule 1	2	4	57.5	5.65	ELPKPSISSN NSNPVEDK	Integral to plasma membrane
						TLTLLSVTR	
						TLTLLSVTR	
						TLTLLSVTR	
ZN503_HUMAN	Zinc finger protein 503	2	4	62.5	8.94	KEPGGGGGGG GGGGGGGGGV SS	Nucleus
						KEPGGGGGGG GGGGGGGGGV SS	
						KEPGGGGGGG GGGGGGGGGV SS	
						PGGGGGGGGG GGG	
HXD11_HUMAN	Homeobox protein Hox-D11	2	2	35.5	9.43	SPMGAADKGD PR	Nucleus
						RTGAGGGGGS PCTK	

FZD8_HUMAN	Frizzled-8 precursor	2	2	73.3	8.58	AGGAGGAAAG AGAAGAGAGG PGGR	Integral to membrane
						GGAGGAAAGA GAAGAGAGGP GGR	
ZIC2_HUMAN	Zinc finger protein ZIC 2	2	3	55	8.68	SYTHPSSLRK HMK 413vhes	Nucleus
						GGGSGSGGAG GGSGGGSGSG G	
						GGGSGSGGAG GGSGGGSGSG G	
HNRPL_HUMAN	Heterogeneous nuclear ribonucleoprotein L	3	3	60.1	6.65	MAAAGGGGGG GRYYGGGSEG GR	Nucleus
						TDNAGDQHGG GGGGGGGAGA A	
						HGGGGGGGGG AGAAGGGGGG ENY	
FLRT1_HUMAN	Leucine-rich repeat transmembrane protein FLRT1	2	2	71.3	5.95	IPADIPDDAT TLYLQNNQIN NAGIPQDLK	Integral to membrane
						LQNLTELSLV RNSLAAPPLN LPSAHLQKLY LQDNAISHIP	
						LQNLTELSLV RNSLAAPPLN LPSAHLQKLY LQDNAISHIP	
ACTK_HUMAN	Beta-actin-like protein 3	2	4	42	5.91	QAVFPSIVGR PR	Cytoplasm
						QAVFPSIVGR PR	
						AVFPSIVGRP R	
						AVFPSIVGRP R	
DSPP_HUMAN	Dentin sialophosphoprotein precursor	2	2	126.4	3.6	SDSSNSSDSS DSSDSSDSSD SDSSNR	Extracellular region
						NSSDSSDSSD SSDSSDSDSS NR	
CO4A5_HUMAN	Collagen alpha-5(IV) chain	2	2	160.9	7.71	GQAGATGPKG LPGIPGAPGA	Basement membrane
						GLNGMKGDPG LPGVPGFPGM K	
AIM1_HUMAN	Absent in melanoma 1 protein	2	2	188.6	5.61	QPPPASSPTK RK	Unknown
						GAPGASDADG LKPR	
ATP7B_HUMAN	Copper-transporting ATPase 2	2	2	157.2	6.29	MVGTGVAAQN GILIKGGKPL EMAHK	Integral to plasma membrane
						LQPWMGSAAM AASSVSVVLS SLQLK	

PRDM8_HUMAN	PR domain zinc finger protein 8	2	2	71.7	8.21	SGSGSGGGGG HQEAELSPDG IATGGGK	Nucleus
						KLQGAADLNG GCGSLPSGGG GLPK	
HXB3_HUMAN	Homeobox protein Hox-B3	2	2	44.3	9.27	GGGSGGSGGG GGGGGGGDK	Nucleus
						GGSGGSGGGG GGGGGGDK	
OTUD5_HUMAN	OTU domain-containing protein 5	2	2	60.6	6.1	VGVGGGGTGV GGGDR	
						ASATCSSATA AASS	
CO2A1_HUMAN	Collagen alpha-1(II) chain	3	3	141.7	6.58	TDGPKGASGP AGPPGAQGPP GLQGMPGER	Basement membrane
						GDSGPPGRAG EPGLQGPAGP PG	
						QGDRGEAGAQ GPMGPSGPAG ARGIQGPQ	
						QGDRGEAGAQ GPMGPSGPAG ARGIQGPQ	
CO4A3_HUMAN	Collagen alpha-3(IV) chain	2	2	161.7	9.28	QGAAGLKGSP GSPGNTGL	Basement membrane
						GEPGIPGIGF PGPPGPK	

Supplemental Table 2. Membrane and membrane-associated proteins identified in BxPC-3 cells. Highlighted proteins were only found in BxPC-3 cells.

Accession #	Protein name	Unique peptides	Total peptides	Mr (KDa)	PI	Peptides identified	Subcellular localization
MYOF_HUMAN	Myoferlin	25	43	234.6	5.84	MLRVIVESAS NIPKTK	Integral to membrane
						MLRVIVESAS NIPKTK	
						MLRVIVESAS NIPK	
						VIVESASNIP K	
						GPVGTVSEAQ LAR	
						QLSGNNIRPV VK	
						QLSGNNIRPV VK	
						VSMFVLGTGD EPPPERR	
						NLVDPFVEVS FAGK	
						NLVDPFVEVS FAGK	
						NLVDPFVEVS FAGK	
						ILVELATFLE K	
						IPANQLAELW LK	
						IPANQLAELW LK	
						SRSLSQIHEA AVR	
						IPAHQVLYST SGENASGK	
						SLLTEADAGH TEFTDEVYQN ESR	
						SLLTEADAGH TEFTDEVYQN ESR	
						VIMELFDNDQ VGKDEFLGR	
						VIMELFDNDQ VGKDEFLGR	
						VIMELFDNDQ VGKDEFLGR	
						TPNFPSSVLF MK	
						TPNFPSSVLF MK	
						VFLPKEELYM PPLVIK	

						VFLPKEELYM PPLVIK	
						IYPLPDDPSV PAPPR	
						IYPLPDDPSV PAPPR	
						VIEDRDHYIP NTLNPVFGR	
						VIEDRDHYIP NTLNPVFGR	
						MYELSCYLPQ EKDLK	
						VGETIIDLEN RFLSR	
						VGETIIDLEN RFLSR	
						DQLRPTQLLQ NVAR	
						DQLRPTQLLQ NVAR	
						DQLRPTQLLQ NVAR	
						FKGFPQPILS EDGSR	
						ILHQHLGAPE ER	
						TQGLVPEHVE TR	
						TQGLVPEHVE TR	
						LQMWVDVFPK	
						LQMWVDVFPK	
						SLGPPGPPFN ITPR	
						SLGPPGPPFN ITPR	
PEPL_HUMAN	Periplakin	3	4	204.5	5.44	ETPLKPIPVE ALCDFEGEQG LISR	Plasma membrane
						QLLAGLDKVA SDLDRQEK	
						QLLAGLDKVA SDLDRQEK	
						SLLGEVEQNL QAAK	
CLH1_HUMAN	Clathrin heavy chain 1	4	6	191.5	5.48	ISGETIFVTA PHEATAGIIG VNR	Plasma membrane
						IHEGCEEPAT HNALAK	
						ESYVETELIF ALAK	
						ESYVETELIF ALAK	

						LASTLVHLGE YQAAVDGAR	
						LASTLVHLGE YQAAVDGAR	
PLEC1_HUMAN	Plectin-1	2	4	531.4	5.73	CRPDQLTGLS LLPLSEK	Plasma membrane
						CRPDQLTGLS LLPLSEK	
						APVPASELLA SGVLSR	
						APVPASELLA SGVLSR	
LPPRC_HUMAN	Leucine-rich PPR motif-containing protein	3	3	157.8	5.81	DLPVTEAVFS ALVTGHAR	Membrane
						SYVSEKDVTS AK	
						YAGEPVPFIE PPESFEFYAQ QLRK	
FA62A_HUMAN	Extended synaptotagmin-1	2	3	122.8	5.57	ALTLGALTLP LAR	Membrane
						ALTLGALTLP LAR	
						LTPRPTAAEL EEVLQVNSLI QTQK	
ACTN4_HUMAN	Alpha-actinin-4	23	58	104.8	5.27	KTFTAWCNSH LR	Extracellular region
						FAIQDISVEE TSAKEGLLLW CQR	
						FAIQDISVEE TSAKEGLLLW CQR	
						EGLLLWCQR	
						DGLAFNALIH R	
						DGLAFNALIH R	
						AIMTYVSSFY HAFSGAQKAE TAANR	
						AIMTYVSSFY HAFSGAQKAE TAANR	
						CQLEINFNTL QTK	
						CQLEINFNTL QTK	
						LSNRPAFMPS EGK	
						LSNRPAFMPS EGK	
						VEQIAAIAQE LNELDYYDSH NVNTR	
						VEQIAAIAQE LNELDYYDSH NVNTR	

CQKICDQWDA LGSLTHSR
CQKICDQWDA LGSLTHSR
ICDQWDALGS LTHSR
ICDQWDALGS LTHSR
QLEAIDQLHL EYAKR
QLEAIDQLHL EYAKR
QLEAIDQLHL EYAK
QLEAIDQLHL EYAK
QLEAIDQLHL EYAK
QLEAIDQLHL EYAK
LSGSNPYTTV TPQIINSKWE K
LSGSNPYTTV TPQIINSKWE K
LSGSNPYTTV TPQIINSKWE K
LSGSNPYTTV TPQIINSK
LSGSNPYTTV TPQIINSK
VQQLVPKRDH ALLEEQSK
QFASQANVVG PWIQTK
QFASQANVVG PWIQTK
QFASQANVVG PWIQTK
QFASQANVVG PWIQTK
QFASQANVVG PWIQTK
QFASQANVVG PWIQTK
QFASQANVVG PWIQTK
QFASQANVVG PWIQTK
VGWEQLLTTI AR
VGWEQLLTTI AR
VGWEQLLTTI AR
VGWEQLLTTI AR
VGWEQLLTTI AR

						VGWEQLLTTI AR	
						TINEVENQIL TR	
						TINEVENQIL TR	
						GISQEQMQEF R	
						GISQEQMQEF R	
						ACLISLGYDV ENDRQGEAEF NR	
						ACLISLGYDV ENDRQGEAEF NR	
						ETTDTDTADQ VIASFK	
						ETTDTDTADQ VIASFK	
						ETTDTDTADQ VIASFK	
						ETTDTDTADQ VIASFK	
						MAPYQGPDAV PGALDYKSFS TALYGESDL	
						MAPYQGPDAV PGALDYKSFS TALYGESDL	
						MAPYQGPDAV PGALDYKSFS TALYGESDL	
						MAPYQGPDAV PGALDYKSFS TALYGESDL	
						MAPYQGPDAV PGALDYK	
						MAPYQGPDAV PGALDYK	
ENPL_HUMAN	Endoplasmin	16	31	92.4	4.76	EGSRTDDEVV QREEEAIQLD GLNASQIR	Endoplasm reticulum membrane
						EGSRTDDEVV QREEEAIQLD GLNASQIR	
						EGSRTDDEVV QREEEAIQLD GLNASQIR	

EGSRTDDEVV QREEEAIQLD GLNASQIR

EGSRTDDEVV QREEEAIQLD GLNASQIR

EGSRTDDEVV QREEEAIQLD GLNASQIR

TDDEVVQREE EAIQLDGLNA SQIR
TDDEVVQREE EAIQLDGLNA SQIR
ELISNASDAL DKIR
ELISNASDAL DKIR
LISLTDENAL SGNEELTVK
LISLTDENAL SGNEELTVK
SILFVPTSAP R
SILFVPTSAP R
SILFVPTSAP R
GLFDEYGSK
GVVDSDDLPL NVSR
GVVDSDDLPL NVSR
IADDKYNDTF WKEFGTNIK
IADDKYNDTF WKEFGTNIK
FQSSHHPTDI TSLDQYVER
FQSSHHPTDI TSLDQYVER
KGYEVIYLTE PVDEYCIQAL PEFDGKR
GYEVIYLTEP VDEYCIQALP EFDGKR
GYEVIYLTEP VDEYCIQALP EFDGKR
GYEVIYLTEP VDEYCIQALP EFDGKR
GYEVIYLTEP VDEYCIQALP EFDGKR
EAVEKEFEPL LNWMKDK
ALKDKIEKAV VSQR

						LTESPCALVA SQYGWSGNME R	
						LTESPCALVA SQYGWSGNME R	
						AQAYQTGKDI STNYYASQKK	
						SGYLLPDTK	
ACTN1_HUMAN	Alpha-actinin-1	4	6	103	5.25	KTFTAWCNSH LR	Cytoplasm
						FAIQDISVEE TSAKEGLLLW CQR	
						FAIQDISVEE TSAKEGLLLW CQR	
						EGLLLWCQR	
						LRKDDPLTNL NTAFDVAEK	
						LRKDDPLTNL NTAFDVAEK	
						AIMTYVSSFY HAFSGAQKAE TAANR	
						AIMTYVSSFY HAFSGAQKAE TAANR	
						CQLEINFNTL QTK	
						CQLEINFNTL QTK	
						LAILGIHNEV SK	
						QFGAQANVIG PWIQTK	
						IDQLEGDHQL IQEALIFDNK	
						IDQLEGDHQL IQEALIFDNK	
						VGWEQLLTTI AR	
						VGWEQLLTTI AR	
						VGWEQLLTTI AR	
						VGWEQLLTTI AR	
						VGWEQLLTTI AR	
						VGWEQLLTTI AR	
						TINEVENQIL TR	
						TINEVENQIL TR	
GANAB_HUMAN	Neutral alpha-glucosidase AB	7	10	106.8	5.74	FRIDELEPR	Endoplasmic reticulum
						QYASLTGTQA LPPLFSLGYH QSR	

						QYASLTGTQA LPPLFSLGYH QSR	
						DVHNIYGLYV HMATADGLR	
						HHGPQTLYLP VTLSSIPVFQ R	
						YLPVTLSSIP VFQR	
						VVIIGAGKPA AVVLQTK	
						LSFQHDPETS VLVLR	
						LSFQHDPETS VLVLR	
						LSFQHDPETS VLVLR	
AT1A1_HUMAN	Sodium/potassium-transporting ATPase subunit alpha-1	3	4	112.8	5.13	MSINAEEVVV GDLVEVK	Integral to membrane
						GVGIISEGNE TVEDIAAR	
						GVGIISEGNE TVEDIAAR	
						LNIPVSQVNP R	
TERA_HUMAN	Transitional endoplasmic reticulum ATPase	3	6	89.3	5.14	IVSQLLTLMD GLKQR	Endoplasmic reticulum
						QAAPCVLFFD ELDSIAK	
						QAAPCVLFFD ELDSIAK	
						QAAPCVLFFD ELDSIAK	
						QAAPCVLFFD ELDSIAK	
						LDQLIYIPLP DEK	
IMB1_HUMAN	Importin subunit beta-1	3	4	97.1	4.68	MELITILEK	Nucleus envelope
						MELITILEK	
						LAATNALLNS LEFTK	
						AFGCILEGPE PSQLKPLVIQ AMPTLIELMK DPSVVVRDTA AWTVGR	
HNRL2_HUMAN	Heterogeneous nuclear ribonucleoprotein U-like protein 2	2	2	85.1	4.85	YGGQPLFSEK FPTLWSGAR	Nucleus
						YNVLGAETVL NQMR	

HXK1_HUMAN	Hexokinase-1	2	3	102.4	6.36	LSDETLIDIM TR<br>LSDETLIDIM TR<br>NHEKNQNVHM ESEVYDTPEN IVHGSGSQLF DHVAECLGDF MEKR<br>NHEKNQNVHM ESEVYDTPEN IVHGSGSQLF DHVAECLGDF MEKR<br>NHEKNQNVHM ESEVYDTPEN IVHGSGSQLF DHVAECLGDF MEKR<br>NHEKNQNVHM ESEVYDTPEN IVHGSGSQLF DHVAECLGDF MEKR	Membrane
LONM_HUMAN	Lon protease homolog	2	2	106.4	6.01	ILEFIAVSQL R<br>IVSGEAESVE VTPENLQDFV GKPVFTVER	Mitochondrion
WDR89_HUMAN	WD repeat-containing protein 89	2	2	43.2	5.72	FHPSNPNMVV SGSSDGLVNV FDINIDNEED ALVTTCNSIS SV<br>IHLMNCSMSG LTHVTSLQGG HAATVRSFCW NVQDDSLLTG	
ZN367_HUMAN	Zinc finger protein 367	2	2	38.4	8.07	GRGAPPPAAS ASAAASGGED EE<br>GAPPPAASAS AAASGGE	Nucleus
CALX_HUMAN	Calnexin	6	10	67.5	4.47	APVPTGEVYF ADSFDRGTLS GWILSK<br>APVPTGEVYF ADSFDR<br>APVPTGEVYF ADSFDR<br>GTLSGWILSK<br>GTLSGWILSK<br>LYTLILNPDN SFEILVDQSV VNSGNLL<br>KIPNPDFFED LEPFR<br>KIPNPDFFED LEPFR<br>IPNPDFFEDL EPFR	Integral to membrane

						IPNPDFFEDL EPFR	
PDIA4_HUMAN	Protein disulfide-isomerase A4	7	11	72.9	4.96	IANILKDKDP PIPVAK	Endoplasmic reticulum (lumen)
						IDATSASVLA SRFDVSGYPT IK	
						IDATSASVLA SRFDVSGYPT IK	
						FDVSGYPTIK	
						KGQAVDYEGS RTQEEIVAK	
						EVSQPDWTPP PEVTLVLTK	
						EVSQPDWTPP PEVTLVLTK	
						FDVSGYPTLK	
						YGIVDYMIEQ SGPPSKEILT LK	
						YGIVDYMIEQ SGPPSKEILT LK	
						YGIVDYMIEQ SGPPSKEILT LK	
						FAMEPEEFDS DTLREFVTAF K	
NUCL_HUMAN	Nucleolin	5	10	76.6	4.6	QKVEGTEPTT AFNLFVGNLN FNK	Cell cortex
						VEGTEPTTAF NLFVGNLNFN K	
						VEGTEPTTAF NLFVGNLNFN K	
						VEGTEPTTAF NLFVGNLNFN K	
						VEGTEPTTAF NLFVGNLNFN K	
						TGISDVFAKN DLAVVDVR	
						TGISDVFAKN DLAVVDVR	
						FGYVDFESAE DLEK	
						NLPYKVTQDE LKEVFEDAAE IR	
						NLPYKVTQDE LKEVFEDAAE IR	
ECHA_HUMAN	Trifunctional enzyme subunit alpha	4	7	82.9	9.16	TVLGTPEVLL GALPGAGGTQ R	Mitochondrial inner membrane.
						TVLGTPEVLL GALPGAGGTQ R	
						MGLVDQLVEP LGPGLKPPEE R	
						MGLVDQLVEP LGPGLKPPEE R	

						TIEYLEEVAI TFAK	
						TIEYLEEVAI TFAK	
						EVEAVIPDHC IFASNTSALP ISEIAAVSK	
CEAM6_HUMAN	Carcinoembryonic antigen-related cell adhesion molecule 6	3	5	37.2	5.56	VDGNSLIVGY VIGTQQATPG PAYSGR	Integral to plasma membrane
						SDPVTLNVLY GPDVPTISPS K	
						SDPVTLNVLY GPDVPTISPS K	
						PVTLNVLYGP DVPTISPSK	
						PVTLNVLYGP DVPTISPSK	
4F2_HUMAN	4F2 cell-surface antigen heavy chain	2	4	57.9	5.2	IKVAEDEAEA AAAAK	Integral to membrane
						GQSEDPGSLL SLFR	
						GQSEDPGSLL SLFR	
						GQSEDPGSLL SLFR	
P5CS_HUMAN	Delta-1-pyrroline-5-carboxylate synthetase	2	3	87.2	6.66	SNIPFITVPL SR	Mitochondrial inner membrane.
						GPVGLEGLLT TK	
						GPVGLEGLLT TK	
WDR26_HUMAN	WD repeat-containing protein 26	2	2	72.1	5.74	MQANGAGGGG GGGGGGGGGG GGGGGQGQ	Cytoplasm
						MQANGAGGGG GGGGGGGGGG GGGGG	
GRP78_HUMAN	78 kDa glucose-regulated protein	31	91	72.3	5.07	ITPSYVAFTP EGERLIGDAA K	Integral to endoplasm reticulum
						ITPSYVAFTP EGERLIGDAA K	
						ITPSYVAFTP EGER	
						ITPSYVAFTP EGER	
						LIGDAAKNQL TSNPENTVFD AKR	
						LIGDAAKNQL TSNPENTVFD AKR	

NQLTSNPENT VFDAK
TWNDPSVQQD IKFLPFK
TWNDPSVQQD IK
KTKPYIQVDI GGGQTK
TKPYIQVDIG GGQTK
TKPYIQVDIG GGQTK
TKPYIQVDIG GGQTK
TFAPEEISAM VLTK
TFAPEEISAM VLTK
TFAPEEISAM VLTK
TFAPEEISAM VLTK
TFAPEEISAM VLTK
TFAPEEISAM VLTK
TFAPEEISAM VLTK
TFAPEEISAM VLTK
TFAPEEISAM VLTK
TFAPEEISAM VLTK
TFAPEEISAM VLTK
TFAPEEISAM VLTK
TFAPEEISAM VLTK
TFAPEEISAM VLTK
TFAPEEISAM VLTK
MKETAEAYLG KK
KVTHAVVTVP AYFNDAQR
VTHAVVTVPA YFNDAQR
VTHAVVTVPA YFNDAQR
QATKDAGTIA GLNVMR
QATKDAGTIA GLNVMR
DAGTIAGLNV MR

IINEPTAAAI AYGLDKR
IINEPTAAAI AYGLDKR
IINEPTAAAI AYGLDK
IINEPTAAAI AYGLDK
IEIESFYEGE DFSETLTR
IEIESFYEGE DFSETLTR
IEIESFYEGE DFSETLTR
IEIESFYEGE DFSETLTR
IEIESFYEGE DFSETLTR
AKFEELNMDL FR
AKFEELNMDL FR
AKFEELNMDL FR
AKFEELNMDL FR
AKFEELNMDL FR
AKFEELNMDL FR
AKFEELNMDL FR
AKFEELNMDL FR
AKFEELNMDL FR
AKFEELNMDL FR
FEELNMDLFR
FEELNMDLFR
FEELNMDLFR
VLEDSDLKKS DIDEIVLVGG STR
VLEDSDLKKS DIDEIVLVGG STR
KSDIDEIVLV GGSTRIPK
IQQLVKEFFN GKEPSR
IQQLVKEFFN GKEPSR
IQQLVKEFFN GKEPSR
IQQLVKEFFN GKEPSR

						LIPRNTVVPT KKSQIFSTAS DNQPTVTIKV YEG 468erpl	
						KSQIFSTASD NQPTVTIK	
						SQIFSTASDN QPTVTIK	
						SQIFSTASDN QPTVTIK	
						SQIFSTASDN QPTVTIK	
						SQIFSTASDN QPTVTIK	
						SQIFSTASDN QPTVTIK	
						DNHLLGTFDL TGIPPAPR	
						DNHLLGTFDL TGIPPAPR	
						DNHLLGTFDL TGIPPAPR	
						DNHLLGTFDL TGIPPAPR	
						DNHLLGTFDL TGIPPAPR	
						DNHLLGTFDL TGIPPAPR	
						DNHLLGTFDL TGIPPAPR	
						DNHLLGTFDL TGIPPAPR	
						DNHLLGTFDL TGIPPAPR	
						DNHLLGTFDL TGIPPAPR	
						ITITNDQNRL TPEEIER	
						IDTRNELESY AYSLK	
						IDTRNELESY AYSLK	
						KKELEEIVQP IISK	
						KKELEEIVQP IISK	
						KKELEEIVQP IISK	
						ELEEIVQPII SK	
						ELEEIVQPII SK	
						LYGSAGPPPT GEEDTAEKDE L	
						LYGSAGPPPT GEEDTAEKDE L	
GRP75_HUMAN	Stress-70 protein	17	55	73.6	5.87	TTPSVVAFTA DGERLVGMPA KR	Cell surface

TTPSVVAFTA DGERLVGMPA KR
TTPSVVAFTA DGERLVGMPA KR
TTPSVVAFTA DGERLVGMPA KR
TTPSVVAFTA DGER
TTPSVVAFTA DGER
QAVTNPNNTF YATKR
LYSPSQIGAF VLMK
LYSPSQIGAF VLMK
LYSPSQIGAF VLMK
LYSPSQIGAF VLMK
LYSPSQIGAF VLMK
LYSPSQIGAF VLMK
LYSPSQIGAF VLMK
LYSPSQIGAF VLMK
MKETAENYLG HTAK
NAVITVPAYF NDSQR
NAVITVPAYF NDSQR
NAVITVPAYF NDSQR
NAVITVPAYF NDSQR
QATKDAGQIS GLNVLR
QATKDAGQIS GLNVLR
DAGQISGLNV LR
DAGQISGLNV LR
GVFEVKSTNG DTFLGGEDFD QALLR
GVFEVKSTNG DTFLGGEDFD QALLR
STNGDTFLGG EDFDQALLR
STNGDTFLGG EDFDQALLR
STNGDTFLGG EDFDQALLR
STNGDTFLGG EDFDQALLR

						STNGDTFLGG EDFDQALLR	
						STNGDTFLGG EDFDQALLR	
						STNGDTFLGG EDFDQALLR	
						AQFEGIVTDL IRR	
						AQFEGIVTDL IRR	
						AQFEGIVTDL IR	
						AQFEGIVTDL IR	
						AQFEGIVTDL IR	
						AQFEGIVTDL IR	
						AQFEGIVTDL IR	
						AMQDAEVSKS DIGEVILVGG MTR	
						AMQDAEVSKS DIGEVILVGG MTR	
						VQQTVQDLFG R	
						VQQTVQDLFG R	
						EMAGDNKLLG QFTLIGIPPA PR	
						EMAGDNKLLG QFTLIGIPPA PR	
						EMAGDNKLLG QFTLIGIPPA PR	
						EMAGDNKLLG QFTLIGIPPA PR	
						EMAGDNKLLG QFTLIGIPPA PR	
						LLGQFTLIGI PPAPR	
						LLGQFTLIGI PPAPR	
						LLGQFTLIGI PPAPR	
						LLGQFTLIGI PPAPR	
						LLGQFTLIGI PPAPR	
						QAASSLQQAS LKLFEMAYKK	
						QAASSLQQAS LKLFEMAYKK	
HSP71_HUMAN	Heat shock 70 kDa protein 1	7	15	70	5.48	TTPSYVAFTD TER	Cytoplasam
						AFYPEEISSM VLTK	
						AFYPEEISSM VLTK	

						MKEIAEAYLG YPVTNAVITV PAYFNDSQR	
						NAVITVPAYF NDSQR	
						NAVITVPAYF NDSQR	
						NAVITVPAYF NDSQR	
						NAVITVPAYF NDSQR	
						QATKDAGVIA GLNVLR	
						QATKDAGVIA GLNVLR	
						QATKDAGVIA GLNVLR	
						QATKDAGVIA GLNVLR	
						DAGVIAGLNV LR	
						IINEPTAAAI AYGLDR	
						IINEPTAAAI AYGLDR	
						IINEPTAAAI AYGLDR	
						IINEPTAAAI AYGLDR	
						ELEQVCNPII SGLYQGAGGP GPGGFGAQGP K	
						ELEQVCNPII SGLYQGAGGP GPGGFGAQGP K	
HSP7C_HUMAN	Heat shock cognate 71 kDa protein	6	11	70.9	5.37	TTPSYVAFTD TER	Cytoplasm
						SFYPEEVSSM VLTK	
						SFYPEEVSSM VLTK	
						SFYPEEVSSM VLTK	
						SFYPEEVSSM VLTK	
						QATKDAGTIA GLNVLR	
						QATKDAGTIA GLNVLR	
						DAGTIAGLNV LR	
						IINEPTAAAI AYGLDK	

						IINEPTAAAI AYGLDK	
						ARFEELNADL FRGTLDPVEK	
						ARFEELNADL FR	
						NTTIPTKQTQ TFTTYSDNQP GVLIQVYEGE R	
						NTTIPTKQTQ TFTTYSDNQP GVLIQVYEGE R	
TRAP1_HUMAN	Heat shock protein 75 kDa	3	8	80.1	8.3	GTITIQDTGI GMTQEELVSN LGTIAR	Mitochondrion
						GTITIQDTGI GMTQEELVSN LGTIAR	
						GTITIQDTGI GMTQEELVSN LGTIAR	
						GTITIQDTGI GMTQEELVSN LGTIAR	
						YESSALPSGQ LTSLSEYASR	
						YESSALPSGQ LTSLSEYASR	
						AQLLQPTLEI NPR	
						AQLLQPTLEI NPR	
RIB1_HUMAN	Dolichyl-diphosphooligosaccharide--protein glycosyltransferase subunit 1	6	12	68.5	5.96	SSEAPPLINE DVKR	Membrane
						VTAEVVLAHL GGGSTSR	
						VTAEVVLAHL GGGSTSR	
						VTAEVVLAHL GGGSTSR	
						VTAEVVLAHL GGGSTSR	
						ATSFLLALEP ELEAR	
						ATSFLLALEP ELEAR	
						ATSFLLALEP ELEAR	
						ATSFLLALEP ELEAR	
						SEDLLDYGPF R	
						YDYQRQPDSG ISSIR	

						SLETEHKALT SEIALLQSR	
DHSA_HUMAN	Succinate dehydrogenase [ubiquinone] flavoprotein subunit	3	10	72.6	7.06	VSDSISAQYP VVDHEFDAVV VGAGGAGLR	Membrane
						VSDSISAQYP VVDHEFDAVV VGAGGAGLR	
						VSDSISAQYP VVDHEFDAVV VGAGGAGLR	
						VSDSISAQYP VVDHEFDAVV VGAGGAGLR	
						AAFGLSEAGF NTACVTK	
						AAFGLSEAGF NTACVTK	
						LGANSLLDLV VFGR	
						LGANSLLDLV VFGR	
						LGANSLLDLV VFGR	
						LGANSLLDLV VFGR	
ACSL3_HUMAN	Long-chain-fatty-acid--CoA ligase 3	2	3	80.4	8.65	VGAPLVCCEI K	Integral to plasma membrane
						LSPEPWTPET GLVTDAFK	
						LSPEPWTPET GLVTDAFK	
GPDM_HUMAN	Glycerol-3-phosphate dehydrogenase	2	4	80.8	7.23	LVQDYGLESE VAQHLAATYG DK	Mitochondrial inner membrane.
						LVQDYGLESE VAQHLAATYG DK	
						LAFLNVQAAE EALPR	
						LAFLNVQAAE EALPR	
CSMD2_HUMAN	CUB and sushi domain-containing protein 2	2	2	379.8	5.69	PKCEVPCGGN ITSSNGTVYS PGFPSPYSSS QDCVWLITVP IGHGVR	Integral to membrane
						LVINCGDPGI PANGLR	

GCN1L_HUMAN	GCN1-like protein 1	2	2	292.6	7.19	ENPGLVEQYL SAILSLEPNQ<br>NYAGMLGLLV QFCTSHKEM<br>LIGNESKGEH VPGF	Cytoplasm
KPYM_HUMAN	Pyruvate kinase isozymes M1/M2	5	7	57.9	7.96	TATESFASDP ILYRPVAVAL DTK<br>TGLIKGSGTA EVELKK<br>GVNLPGAAVD LPAVSEKDIQ DLK<br>LAPITSDPTE ATAVGAVEAS FK<br>LAPITSDPTE ATAVGAVEAS FK<br>LAPITSDPTE ATAVGAVEAS FK<br>GIFPVLCKDP VQEAWAEDVD LR	Cytoplasm
PDIA1_HUMAN	Protein disulfide-isomerase	8	14	57.1	4.76	LAKVDATEES DLAQQYGVR<br>EADDIVNWLK<br>EADDIVNWLK<br>TGPAATTLPD GAAAESLVES SEVAVIGFFK<br>TGPAATTLPD GAAAESLVES SEVAVIGFFK<br>QFLQAAEAID DIPFGITSNS DVFSK<br>QFLQAAEAID DIPFGITSNS DVFSK<br>QFLQAAEAID DIPFGITSNS DVFSK<br>QFLQAAEAID DIPFGITSNS DVFSK<br>HNQLPLVIEF TEQTAPK<br>HNQLPLVIEF TEQTAPK<br>ILEFFGLKKE ECPAVR<br>MDSTANEVEA VKVHSFPTLK<br>FFPASADRTV IDYNGER	Plasma membrane

PDIA3_HUMAN	Protein disulfide-isomerase A3	16	25	56.7	5.98	SDVLELTDDN FESR	Endoplasmic reticulum lumen
						SDVLELTDDN FESR	
						YGVSGYPTLK	
						IFRDGEEAGA YDGPR	
						QAGPASVPLR TEEEFKK	
						FISDKDASIV GFFDDSFSEA HSEFLK	
						FISDKDASIV GFFDDSFSEA HSEFLK	
						FISDKDASIV GFFDDSFSEA HSEFLK	
						VGFFDDSFSE AHSEFLK	
						DLLIAYYDVD YEK	
						DLLIAYYDVD YEK	
						KFLDAGHKLN FAVASR	
						FLDAGHKLNF AVASR	
						KTFSHELSDF GLESTAGEIP VVAIR	
						KTFSHELSDF GLESTAGEIP VVAIR	
						TFSHELSDFG LESTAGEIPV VAIR	
						TFSHELSDFG LESTAGEIPV VAIR	
						FLQDYFDGNL KR	
						LSKDPNIVIA K	
						GFPTIYFSPA NK	
						GFPTIYFSPA NK	
						KYEGGRELSD FISYLQR	
						ELSDFISYLQ R	
						ELSDFISYLQ R	
						ELSDFISYLQ R	
CH60_HUMAN	60 kDa heat shock protein	30	103	61	5.7	ALMLQGVDLL ADAVAVTMGP K	Mitochondrial inner membrane.
						ALMLQGVDLL ADAVAVTMGP K	

ALMLQGVDLL ADAVAVTMGP K
ALMLQGVDLL ADAVAVTMGP K
ALMLQGVDLL ADAVAVTMGP K
ALMLQGVDLL ADAVAVTMGP K
ALMLQGVDLL ADAVAVTMGP K
ALMLQGVDLL ADAVAVTMGP K
ALMLQGVDLL ADAVAVTMGP K
ALMLQGVDLL ADAVAVTMGP K
ALMLQGVDLL ADAVAVTMGP K
ALMLQGVDLL ADAVAVTMGP K
ALMLQGVDLL ADAVAVTMGP K
ALMLQGVDLL ADAVAVTMGP K
ALMLQGVDLL ADAVAVTMGP K
ALMLQGVDLL ADAVAVTMGP K
ALMLQGVDLL ADAVAVTMGP K
ALMLQGVDLL ADAVAVTMGP K
ALMLQGVDLL ADAVAVTMGP K
ALMLQGVDLL ADAVAVTMGP K
ALMLQGVDLL ADAVAVTMGP K
ALMLQGVDLL ADAVAVTMGP K
ALMLQGVDLL ADAVAVTMGP K
ALMLQGVDLL ADAVAVTMGP K
ALMLQGVDLL ADAVAVTMGP K
ALMLQGVDLL ADAVAVTMGP K
TVIIEQSWGS PK
SIDLKDKYKN IGAK
SIDLKDKYKN IGAK
LVQDVANNTN EEAGDGTTTA TVLAR
LVQDVANNTN EEAGDGTTTA TVLAR

LVQDVANNTN EEAGDGTTTA TVLAR
LVQDVANNTN EEAGDGTTTA TVLAR
LVQDVANNTN EEAGDGTTTA TVLAR
GVMLAVDAVI AELKK
GVMLAVDAVI AELKK
GVMLAVDAVI AELKK
GVMLAVDAVI AELKK
GVMLAVDAVI AELKK
GVMLAVDAVI AELKK
GVMLAVDAVI AELK
QSKPVTTPEE IAQVATISAN GDKEIGNIIS DAMK
QSKPVTTPEE IAQVATISAN GDKEIGNIIS DAMK
TLNDELEIIE GMKFDR
TLNDELEIIE GMK
TLNDELEIIE GMK
TLNDELEIIE GMK
TLNDELEIIE GMK
TLNDELEIIE GMK
GQKCEFQDAY VLLSEKK
GQKCEFQDAY VLLSEKK
CEFQDAYVLL SEKK
CEFQDAYVLL SEKK
CEFQDAYVLL SEKK
CEFQDAYVLL SEK
CEFQDAYVLL SEK
CEFQDAYVLL SEK
KISSIQSIVP ALEIANAHR

ISSIQSIVPA LEIANAHR
ISSIQSIVPA LEIANAHR
ISSIQSIVPA LEIANAHR
ISSIQSIVPA LEIANAHR
ISSIQSIVPA LEIANAHR
KPLVIIAEDV DGEALSTLVL NR
KPLVIIAEDV DGEALSTLVL NR
KPLVIIAEDV DGEALSTLVL NR
KPLVIIAEDV DGEALSTLVL NR
KPLVIIAEDV DGEALSTLVL NR
VGEVIVTKDD AMLLK
IQEIIEQLDV TTSEYEKEKL NER
IQEIIEQLDV TTSEYEKEKL NER
LSDGVAVLK
VGGTSDVEVN EKKDRVTDAL NATR
VTDALNATR
AAVEEGIVLG GGCALLR
AAVEEGIVLG GGCALLR
AAVEEGIVLG GGCALLR
AAVEEGIVLG GGCALLR
AAVEEGIVLG GGCALLR
AAVEEGIVLG GGCALLR
AAVEEGIVLG GGCALLR
AAVEEGIVLG GGCALLR
CIPALDSLTP ANEDQKIGIE IIKR
CIPALDSLTP ANEDQKIGIE IIKR
CIPALDSLTP ANEDQKIGIE IIKR
CIPALDSLTP ANEDQKIGIE IIKR
CIPALDSLTP ANEDQKIGIE IIKR

						CIPALDSLTP ANEDQKIGIE IIKR	
						CIPALDSLTP ANEDQKIGIE IIKR	
						CIPALDSLTP ANEDQKIGIE IIKR	
						CIPALDSLTP ANEDQKIGIE IIK	
						CIPALDSLTP ANEDQKIGIE IIK	
						CIPALDSLTP ANEDQK	
						CIPALDSLTP ANEDQK	
						CIPALDSLTP ANEDQK	
						TLKIPAMTIA K	
						NAGVEGSLIV EK	
						NAGVEGSLIV EK	
						IMQSSSEVGY DAMAGDFVNM VEK	
						IMQSSSEVGY DAMAGDFVNM VEK	
						GIIDPTKVVR	
						TALLDAAGVA SLLTTAEVVV TEIPKEEKD	
						TALLDAAGVA SLLTTAEVVV TEIPKEEKD	
						TALLDAAGVA SLLTTAEVVV TEIPKEEK	
						TALLDAAGVA SLLTTAEVVV TEIPKEEK	
CO4A2_HUMAN	Collagen alpha-2(IV) chain precursor	3	3	167.4	8.89	GFPGDAGLPG PPGF	Basement membrane
						GDPGPPGPP	
						GPKGDPGFPG APGTVGA	
EPIPL_HUMAN	Epiplakin	2	2	552.8	5.43	TGSVAGVVLL PEGHK	Cytoplasm
						TGCIAGLLLP GSQER	

NFAC4_HUMAN	Nuclear factor of activated T-cells, cytoplasmic 4	2	2	95.4	5.29	RLGGPGGGAG GAGGGR	Nucleus
						GAVKAAPGGH P	
SFRS1_HUMAN	Splicing factor, arginine/serine-rich 1	3	5	27.7	10.37	GGGGGGGGA PRG	Nuleus
						GGGGGGGGA PR	
						GGGGGGGGAP R	
						GGGGGGGGAP R	
						GGGGGGGGAP R	
MDHM_HUMAN	Malate dehydrogenase	3	4	35.5	8.92	LTLYDIAHTP GVAADLSHIE TK	Mitochondrion inner membrane
						TIIPLISQCT PK	
						VDFPQDQLTA LTGR	
						VDFPQDQLTA LTGR	
G3P_HUMAN	Glyceraldehyde-3-phosphate dehydrogenase	2	2	36	8.57	LVINGNPITI FQER	Membrane
						VPTANVSVVD LTCR	
RAB2A_HUMAN	Ras-related protein Rab-2A	2	3	23.5	6.08	GAAGALLVYD ITR	Membrane
						TASNVEEAFI NTAK	
						TASNVEEAFI NTAK	
CPNS1_HUMAN	Calpain small subunit 1	2	7	28.3	5.05	GGGGGGGGGG GLGGGLGNVL GG	Plasma membrane
						GGGGGGGGGG GLGGGLGNVL GG	
						GGGGGGGGGG GLGGGLGNVL GG	
						GGGGGGGGGG GLGGGLGNVL GG	
						GGGGGGGGGG GLGG	
						GGGGGGGGGG GLGG	
						GGGGGGGGGG GLGG	

COGA1_HUMAN	Collagen alpha-1(XVI) chain	2	2	157.7	8.14	GDPGPAGPPG	Extracellular matrix
						GDPGAAGQKG QA	
TRI67_HUMAN	Tripartite motif-containing protein 67	2	2	80.9	6.92	GAGGSAAGGL GGGAGGGGDH ADK	Cytoplasm
						AKHEVKPLGA MWK	
ACTB_HUMAN	Actin, cytoplasmic 1	5	21	41.7	5.29	AVFPSIVGRP R	Cytoplasm
						AVFPSIVGRP R	
						AVFPSIVGRP R	
						AVFPSIVGRP R	
						AVFPSIVGRP R	
						AVFPSIVGR	
						AVFPSIVGR	
						AVFPSIVGR	
						AVFPSIVGR	
						VAPEEHPVLL TEAPLNPK	
						VAPEEHPVLL TEAPLNPK	
						VAPEEHPVLL TEAPLNPK	
						VAPEEHPVLL TEAPLNPK	
						VAPEEHPVLL TEAPLNPK	
						VAPEEHPVLL TEAPLNPK	
						VAPEEHPVLL TEAPLNPK	
						SYELPDGQVI TIGNER	
						SYELPDGQVI TIGNER	
						SYELPDGQVI TIGNER	
						SYELPDGQVI TIGNER	
						QEYDESGPSI VHR	
ADT2_HUMAN	ADP/ATP translocase 2	2	5	32.9	9.76	DFLAGGVAAA ISK	Integral to membrane

						DFLAGGVAAA ISK	
						DFLAGGVAAA ISK	
						YFPTQALNFA FK	
						YFPTQALNFA FK	
PDIA6_HUMAN	Protein disulfide-isomerase A6	2	4	48.1	4.95	LAAVDATVNQ VLASR	Endoplasmic reticulum lumen
						LAAVDATVNQ VLASR	
						LAAVDATVNQ VLASR	
						GSFSEQGINE FLR	
SERPH_HUMAN	Serpin H1	2	4	46.4	8.75	DEEVHAGLGE LLR	Endoplasmic reticulum lumen.
						HLAGLGLTEA IDK	
						HLAGLGLTEA IDK	
						HLAGLGLTEA IDK	
DHX57_HUMANI	Putative ATP-dependent RNA helicase DHX57	2	3	155.5	7.83	GGGGGGGGGG GGNRK	Endoplasmic reticulum lumen.
						GGGGGGGGNR K	
						GGGGGGGGNR K	
IRS2_HUMAN	Insulin receptor substrate 2	2	2	137.2	8.9	AAVPSAGPAG PAPTSAAGR	Plasma membrane
						SPKPGAPSGH PVGSLDG	
ITB4_HUMAN	Integrin beta-4	2	2	202	5.74	LLELQEVDSL LR	Plasma membrane
						HRMTTTSAAA YGTHLSPHVP HRVLSTSS	
PPRC1_HUMAN	Peroxisome proliferator-activated receptor gamma coactivator-related protein 1	5	5	177.4	6.11	SSSSSSSSSS SSSSSSSSSS R	Nucleus
						SSSSSSSSSS SSSSSSSSSR	

						SSSSSSSSSS SSSSSSSR	
						SSSSSSSSSS SSSSR	
						SSSSSSSSSS SSSR	
ATPB_HUMAN	ATP synthase subunit beta	4	13	56.5	5.26	IPVGPETLGR	Mitochondrial inner membrane
						IPVGPETLGR	
						IPVGPETLGR	
						VALTGLTVAE YFR	
						VALTGLTVAE YFR	
						VALTGLTVAE YFR	
						FTQAGSEVSA LLGR	
						FTQAGSEVSA LLGR	
						FTQAGSEVSA LLGR	
						FTQAGSEVSA LLGR	
						FTQAGSEVSA LLGR	
						FTQAGSEVSA LLGR	
						AIAELGIYPA VDPLDSTSR	
CH10_HUMAN	10 kDa heat shock protein	2	9	10.9	8.89	VLQATVVAVG SGSK	Mitochondrion
						VLQATVVAVG SGSK	
						VLQATVVAVG SGSK	
						VLQATVVAVG SGSK	
						VLQATVVAVG SGSK	
						VLQATVVAVG SGSK	
						VLQATVVAVG SGSK	
						VLQATVVAVG SGSK	
						VVLDDKDYFL FR	
ACTK_HUMAN	Beta-actin-like protein 3	3	14	42	5.91	QAVFPSIVGR PR	Cytoplasm
						QAVFPSIVGR PR	
						QAVFPSIVGR PR	

						QAVFPSIVGR PR	
						QAVFPSIVGR PR	
						QAVFPSIVGR PR	
						AVFPSIVGRP R	
						AVFPSIVGRP R	
						AVFPSIVGRP R	
						AVFPSIVGRP R	
						AVFPSIVGRP R	
						AVFPSIVGRP R	
						AVFPSIVGRP R	
						SYELPDGQVI TIGNER	
ATPA_HUMAN	ATP synthase subunit alpha	3	6	59.7	9.16	VLSIGDGIAR	Mitochondrial inner membrane.
						VLSIGDGIAR	
						TGAIVDVPVG EELLGR	
						TGAIVDVPVG EELLGR	
						TGAIVDVPVG EELLGR	
						AVDSLVPIGR	
CO4A4_HUMAN	Collagen alpha-4(IV) chain precursor	4	4	163.9	8.87	GSRGPPGPPG PQGP	Basement membrane
						GLCACEPGPM GPPGPPGLPG	
						LCACEPGPMG PPGPPGLP	
						GASGLHDVGP PGPV	
ECH1_HUMAN	Delta(3,5)-Delta(2,4)-dienoyl-CoA isomerase	2	6	35.8	8.16	GNQSLVNELA FTAR	Mitochondrion
						GNQSLVNELA FTAR	
						GNQSLVNELA FTAR	
						GNQSLVNELA FTAR	
						NQSLVNELAF TAR	

						NQSLVNELAF TAR	
AATM_HUMAN	Aspartate aminotransferase	2	7	47.4	9.14	EYLPIGGLAE FCK	Plasma membrane
						EYLPIGGLAE FCK<br>EYLPIGGLAE FCK<br>EYLPIGGLAE FCK<br>FVTVQTISGT GALR<br>FVTVQTISGT GALR<br>FVTVQTISGT GALR	
GLYM_HUMAN	Serine hydroxymethyltransferase	2	4	56	8.76	AALEALGSCL NNK	Mitochondrion
						AALEALGSCL NNK<br>AALEALGSCL NNK<br>TGLIDYNQLA LTAR	
HNRPM_HUMAN	Heterogeneous nuclear ribonucleoprotein M	2	2	77.5	8.84	GAPGVPSGNG APGPKGEGER PAQNEK	Integral to plasma membrane
						GAPGVPSGNG APGPKGEGER PAQNEK<br>AMQKVMATTG GMGMGPGGPG MITIPPS	
PO4F2_HUMAN	POU domain, class 4, transcription factor 2	2	2	43.1	9.27	PSSSSNAGGG GGGGGGGGGG GGGR	Nucleus
						GGGGGGGGGG GGGGGRSSSS<br>SSSGSSGGGG SEAMR	
M3K1_HUMAN	Mitogen-activated protein kinase kinase kinase 1	2	2	164.8	8.37	GALKASSAPA AAAGLLR	Membrane fraction
						EKMEAEEEEA LAIAM	
CO4A6_HUMAN	Collagen alpha-6(IV) chain precursor	2	2	163.7	9.31	GDEGIQGLRG PSGVPGLPAL SG	basement membrane
						GFPGFPGLHG LNGLP	

ZO3_HUMAN	Tight junction protein ZO-3	2	2	102.7	6.1	RSPGGGSEAN GLAL	Plasma membrane
						SSYDIYRVPS SQSMEDR	
ANXA2_HUMAN	Annexin A2	2	14	38.6	7.57	STVHEILCK	Basement membrane
						STVHEILCK	Plasma membrane
						STVHEILCK	
						STVHEILCK	
						GVDEVTIVNI LTNR	
						GVDEVTIVNI LTNR	
						GVDEVTIVNI LTNR	
						GVDEVTIVNI LTNR	
						GVDEVTIVNI LTNR	
						GVDEVTIVNI LTNR	
						GVDEVTIVNI LTNR	
						GVDEVTIVNI LTNR	
						GVDEVTIVNI LTNR	
						GVDEVTIVNI LTNR	
M2OM_HUMAN	Mitochondrial 2-oxoglutarate/malate carrier protein	2	2	34	9.92	AVIGMTAGAT GAFVGTPA	Integral to plasma membrane
						VIGMTAGATG AFVGTPAEVA L	
CALR_HUMAN	Calreticulin	9	24	48.1	4.29	EPAVYFKEQF LDGDGWTSR	Cytoplasm
						EPAVYFKEQF LDGDGWTSR	
						EPAVYFKEQF LDGDGWTSR	
						EPAVYFKEQF LDGDGWTSR	
						FYGDEEKDKG LQTSQDAR	
						FYALSASFEP FSNK	

						FYALSASFEP FSNK	
						LFPNSLDQTD MHGDSEYNIM FGPDICGPGT K	
						CKDDEFTHLY TLIVRPDNTY EVK	
						CKDDEFTHLY TLIVRPDNTY EVK	
						CKDDEFTHLY TLIVRPDNTY EVK	
						CKDDEFTHLY TLIVRPDNTY EVK	
						IDNSQVESGS LEDDWDFLPP KK	
						IDNSQVESGS LEDDWDFLPP KK	
						IDNSQVESGS LEDDWDFLPP KK	
						IDNSQVESGS LEDDWDFLPP KK	
						IDNSQVESGS LEDDWDFLPP KK	
						IDNSQVESGS LEDDWDFLPP K	
						IDNSQVESGS LEDDWDFLPP K	
						IKDPDASKPE DWDER	
						SGTIFDNFLI TNDEAYAEEF GNETWGVTK	
						SGTIFDNFLI TNDEAYAEEF GNETWGVTK	
						SGTIFDNFLI TNDEAYAEEF GNETWGVTK	
						SGTIFDNFLI TNDEAYAEEF GNETWGVTK	
DLDH_HUMAN	Dihydrolipoyl dehydrogenase	5	13	54.1	7.95	ADQPIDADVT VIGSGPGGYV AAIK	Mitochondrion
						ADQPIDADVT VIGSGPGGYV AAIK	
						ADQPIDADVT VIGSGPGGYV AAIK	
						ADQPIDADVT VIGSGPGGYV AAIK	
						TVCIEKNETL GGTCLNVGCI PSK	
						TVCIEKNETL GGTCLNVGCI PSK	

						NLGLEELGIE LDPR	
						NLGLEELGIE LDPR	
						IPNIYAIGDV VAGPMLAHK	
						IPNIYAIGDV VAGPMLAHK	
						IPNIYAIGDV VAGPMLAHK	
						IPNIYAIGDV VAGPMLAHK	
						VCHAHPTLSE AFR	
ODO2_HUMAN	Dihydrolipoyllysine-residue succinyltransferase component of 2-oxoglutarate dehydrogenase complex	3	6	48.6	9.01	TPAFAESVTE GDVRWEK	Plasma membrane
						TPAFAESVTE GDVRWEK	
						ASAFALQEQP VVNAVIDDTT KEVVYR	
						ASAFALQEQP VVNAVIDDTT KEVVYR	
						ASAFALQEQP VVNAVIDDTT K	
						ASAFALQEQP VVNAVIDDTT K	
HNRPK_HUMAN	Heterogeneous nuclear ribonucleoprotein K	4	8	50.9	5.39	LLIHQSLAGG IIGVK	Nucleus
						LLIHQSLAGG IIGVK	
						IILDLISESP IK	
						IILDLISESP IK	
						GSYGDLGGPI ITTQVTIPK	
						GSYGDLGGPI ITTQVTIPK	
						IITITGTQDQ IQNAQYLLQN SVK	
						IITITGTQDQ IQNAQYLLQN SVK	
QCR1_HUMAN	Cytochrome b-c1 complex subunit 1	3	5	52.6	5.94	DVVFNYLHAT AFQGTPLAQA VEGPSENVR	Mitochondrial inner membrane.
						DVVFNYLHAT AFQGTPLAQA VEGPSENVR	

						ADLTEYLSTH YKAPR	
						MVLAAAGGVE HQQLLDLAQK	
						MVLAAAGGVE HQQLLDLAQK	
ENOA_HUMAN	Alpha-enolase	3	5	47.1	7.01	LAMQEFMILP VGAANFR	Plasma membrane
						DATNVGDEGG FAPNILENKE GLELLK	
						DATNVGDEGG FAPNILENKE GLELLK	
						YISPDQLADL YK	
						YISPDQLADL YK	
TBB5_HUMAN	Tubulin beta chain	3	3	49.6	4.78	SGPFGQIFRP DNFVFGQSGA GNNWAK	Microtubule
						GHYTEGAELV DSVLDVVRK	
						ALTVPELTQQ VFDAK	
HNRH1_HUMAN	Heterogeneous nuclear ribonucleoprotein H	2	3	49.2	5.89	STGEAFVQFA SQEIAEK	Nucleus
						STGEAFVQFA SQEIAEK	
						ATENDIYNFF SPLNPVR	
TBA1A_HUMAN	Tubulin alpha-1A chain	2	6	50.1	4.94	AVFVDLEPTV IDEVR	Microtubule
						AVFVDLEPTV IDEVR	
						AVFVDLEPTV IDEVR	
						AVFVDLEPTV IDEVR	
						IHFPLATYAP VISAEK	
						IHFPLATYAP VISAEK	
EFTU_HUMAN	Elongation factor Tu	2	3	49.5	7.26	LLDAVDTYIP VPAR	Mitochondrion
						LLDAVDTYIP VPAR	
						DLEKPFLLPV EAVYSVPGR	
ERO1A_HUMAN	ERO1-like protein alpha precursor	2	3	54.4	5.48	HDDSSDNFCE ADDIQSPEAE YVDLLLNPER	Membrane
						HDDSSDNFCE ADDIQSPEAE YVDLLLNPER	

						IWNVIYEENC FKPQTIK	
DHE3_HUMAN	Glutamate dehydrogenase 1	2	2	61.4	7.66	GASIVEDKLV EDLR	Mitochondrion
						ISGASEKDIV HSGLAYTMER	
OAT_HUMAN	Ornithine aminotransferase	4	6	48.5	6.57	AFYNNVLGEY EEYITK	Mitochrion
						HQVLFIADEI QTGLAR	
						WLAVDYENVR PDIVLLGK	
						WLAVDYENVR PDIVLLGK	
						VAIAALEVLE EENLAENADK	
						VAIAALEVLE EENLAENADK	
APMAP_HUMAN	Adipocyte plasma membrane-associated protein	3	5	46.5	5.82	RPLRPQVVTD DDGQAPEAK	Integral to membrane
						LFENQLVGPE SIAHIGDVMF TGTADGR	
						LFENQLVGPE SIAHIGDVMF TGTADGR	
						LFENQLVGPE SIAHIGDVMF TGTADGR	
						TRDDEPVCGR PLGIR	
ACADM_HUMAN	Medium-chain specific acyl-CoA dehydrogenase	2	3	46.6	8.61	AFTGFIVEAD TPGIQIGRK	Mitochrion
						AFAGDIANQL ATDAVQILGG NGFNTEYPVE K	
						AFAGDIANQL ATDAVQILGG NGFNTEYPVE K	
TXND4_HUMAN	Thioredoxin domain-containing protein 4 precursor	2	3	46.9	5.09	QQKSDPIQEI RDLAEITTLD R	Endoplasmic reticulum lumen
						QQKSDPIQEI RDLAEITTLD R	
						VANILHDDCA FLSAFGDVSK PER	
THIM_HUMAN	3-ketoacyl-CoA thiolase	2	4	41.9	8.32	TPFGAYGGLL KDFTATDLSE FAAK	Mitochrial inner membrane
						TPFGAYGGLL KDFTATDLSE FAAK	
						VSPETVDSVI MGNVLQSSSD AIYLAR	

TF_HUMAN	Tissue factor	2	3	33	6.64	VSPETVDSVI MGNVLQSSSD AIYLAR<br>TILEWEPKPV NQVYTVQIST K<br>CFYTTDTECD LTDEIVKDVK<br>CFYTTDTECD LTDEIVKDVK	Membrane
PK1L1_HUMAN	Polycystic kidney disease protein 1-like 1	3	3	315.2	6.64	STVVIHHFPS IPSYNVSFIS QTQVGDSQAW HSMTVWYKM<br>STVVIHHFPS IPSYNVSFIS QTQVGDSQAW HSMTVWYKM<br>STVVIHHFPS IPSYNVSFIS QTQVGDSQAW HSMTVWYKM<br>HPCFDSSTAH QLDAAAPTVS FEAQWLSDSY DQFLVMLR<br>EQTVTIKPYS LSSGETYVLQ VSVASKHGLL GKAQLYLTVN PAPR	Integral to membrane
STML2_HUMAN	Stomatin-like protein 2	2	4	38.5	6.87	ILEPGLNILI PVLDR<br>ILEPGLNILI PVLDR<br>ILAAALTQHN GDAAASLTVA EQYVSAFSK<br>ILAAALTQHN GDAAASLTVA EQYVSAFSK	Membrane
L2HDH_HUMAN	L-2-hydroxyglutarate dehydrogenase	2	2	50.3	8.57	AQALDRDGNL VEDFVFDAGV GDIGNR<br>ILHVRNAPSP AATSSIAISG MIADEVQQR<br>ILHVRNAPSP AATSSIAISG MIADEVQQR	Integral to membrane
KCNQ3_HUMAN	Potassium voltage-gated channel subfamily KQT member 3	2	2	96.7	8.98	VGLAPGDVEQ VTLALGAGAD KDGTL	Integral to membrane

						STSEIEDQSM MGKFVKVERQ<br>VQDMGKKLDF LVDMHMQHME R	
						STSEIEDQSM MGKFVKVERQ<br>VQDMGKKLDF LVDMHMQHME R	
						STSEIEDQSM MGKFVKVERQ<br>VQDMGKKLDF LVDMHMQHME R	
						STSEIEDQSM MGKFVKVERQ<br>VQDMGKKLDF LVDMHMQHME R	
						STSEIEDQSM MGKFVKVERQ<br>VQDMGKKLDF LVDMHMQHME R	
						STSEIEDQSM MGKFVKVERQ<br>VQDMGKKLDF LVDMHMQHME R	
TXND5_HUMAN	Thioredoxin domain-containing protein 5 precursor	2	2	47.6	6.63	DLESLREYVE SQLQR	Endoplasmic reticulum lumen
						GYPTLLLFR	
QCR2_HUMAN	Cytochrome b-c1 complex subunit 2	2	2	48.4	8.74	KVKATAAPAG APPQ	Mitochondrion inner membrane
						AVAFQNPQTH VIENLHAAAY R	
HNRPC_HUMAN	Heterogeneous nuclear ribonucleoproteins C1/C2	3	9	33.6	4.95	VFIGNLNTLV VK	Ribonucleosomes.
						VFIGNLNTLV VK	
						MIAGQVLDIN LAAEPK	
						MIAGQVLDIN LAAEPK	
						MIAGQVLDIN LAAEPK	
						MIAGQVLDIN LAAEPK	
						MIAGQVLDIN LAAEPK	
						MIAGQVLDIN LAAEPK	
						VPPPPPIAR	

ETFA_HUMAN	Electron transfer flavoprotein subunit alpha	3	7	35.1	8.62	QSTLVIAEHA NDSLAPITLN TITAATR	Mitochondrion
						QSTLVIAEHA NDSLAPITLN TITAATR<br>QSTLVIAEHA NDSLAPITLN TITAATR<br>QSTLVIAEHA NDSLAPITLN TITAATR<br>LLYDLADQLH AAVGASR<br>TIVAINKDPE APIFQVADYG IVADLFK<br>TIVAINKDPE APIFQVADYG IVADLFK	
ROA2_HUMAN	Heterogeneous nuclear ribonucleoproteins A2/B1	3	4	37.4	8.97	LFIGGLSFET TEESLR	Nucleus
						LFIGGLSFET TEESLR<br>ALSRQEMQEV QSSR<br>GNFGGSRNMG GPYGGGNYGP<br>GGSGGSGGYG GRSRY	
ANXA1_HUMAN	Annexin A1	4	5	38.7	6.57	GGPGSAVSPY PTFNPSSDVA ALHK	Plasma membrane
						GVDEATIIDI LTKR<br>GVDEATIIDI LTK<br>GLGTDEDTLI EILASR<br>GLGTDEDTLI EILASR	
ROA1_HUMAN	Heterogeneous nuclear ribonucleoprotein A1	2	3	38.8	9.26	LFIGGLSFET TDESLR	Cytoplasm
						LFIGGLSFET TDESLR<br>SSGPYGGGGQ YFAKPR	
HMOX2_HUMAN	Heme oxygenase 2	2	4	36	5.31	IHYIGQNEPE LLVAHAYTR	Plasma membrane
						IHYIGQNEPE LLVAHAYTR<br>ALKLPSTGEG TQFYLFENVD NAQQFK<br>ALKLPSTGEG TQFYLFENVD NAQQFK	

CDKL2_HUMAN	Cyclin-dependent kinase-like 2	2	2	56	8.45	NPSVAIPPLT HNLSAVAPSI NSGMGTETIP IQGYRVDEK	Nucleus
						SGMGTETIPI QGYR	
K1542_HUMAN	PHD and RING finger domain-containing protein 1	2	2	178.5	9.15	LPAAVPEPDL EEEPVPDLLG SILSGQSLLM LGSS	
						QTLAPVPAAL TPASEPASQA TAASNSEEK	
GBB2_HUMAN	Guanine nucleotide-binding protein G(I)/G(S)/G(T) subunit beta-2	2	4	37.3	5.6	SELEQLRQEA EQLR	Cytoplasm
						SELEQLRQEA EQLR	
						KACGDSTLTQ ITAGLDPVGR	
						KACGDSTLTQ ITAGLDPVGR	
ECHM_HUMAN	Enoyl-CoA hydratase	3	12	31.4	8.34	ALNALCDGLI DELNQALK	Mitochondrion
						ALNALCDGLI DELNQALK	
						ALNALCDGLI DELNQALK	
						ALNALCDGLI DELNQALK	
						AQFAQPEILI GTIPGAGGTQ R	
						AQFAQPEILI GTIPGAGGTQ R	
						AQFAQPEILI GTIPGAGGTQ R	
						ICPVETLVEE AIQCAEK	
						ICPVETLVEE AIQCAEK	
						ICPVETLVEE AIQCAEK	
						ICPVETLVEE AIQCAEK	
						ICPVETLVEE AIQCAEK	
KAD2_HUMAN	Adenylate kinase 2	4	7	26.5	7.67	LVSDEMVVEL IEK	Membrane
						LVSDEMVVEL IEK	
						LQAYHTQTTP LIEYYR	
						GIHSAIDASQ TPDVVFASIL AAFSK	

						GIHSAIDASQ TPDVVFASIL AAFSK<br>GIHSAIDASQ TPDVVFASIL AAFSK<br>QTPDVVFASI LAAFSK	
C1QBP_HUMAN	Complement component 1 Q subcomponent-binding protein	2	3	31.3	4.74	VEEQEPELTS TPNFVVEVIK	Plasma membrane
FAM3C_HUMAN	Protein FAM3C	2	3	24.7	8.52	ALVLDCHYPE DEVGQEDEAE SDIFSIR<br>ALVLDCHYPE DEVGQEDEAE SDIFSIR<br>YFDMWGGDVA PFIEFLKAIQ DGTIVLMGTY DDGATKLNDE ARR<br>LIADLGSTSI TNLGFR<br>LIADLGSTSI TNLGFR	Intracecellular region
ALK_HUMAN	ALK tyrosine kinase receptor precursor	2	2	176.3	6.67	MGAIGLLWLL PLLLSTAAVG SGMGTGQRAG SPAAGPPLQP	Integral to plasma membrane
PCD16_HUMAN	Protocadherin-16 precursor	2	2	346	4.79	SHGVSVLGIF NLEKDDMLYI LVGQQGEDAC PS<br>LVLMATDRGS PALVGSATLT VMVIDTNDNR<br>VGAADAGNLS ASVTVSVLVT GEDEYDPVFL APAFHFQVPE GARR	Integral to membrane
PTPRZ_HUMAN	Receptor-type tyrosine-protein phosphatase zeta	2	2	254.4	4.67	VDKISSTMLH LIVSNSASSE NM	Integral to plasma membrane
CO4A5_HUMAN	Collagen alpha-5(IV) chain	3	3	160.9	7.71	ATSELSHSAK SDAGLV<br>GEKGEPGLPG IPGVSGPKGY QGLPGDPGQP GLSGQPGLPG PPGPKGNPG<br>GQKGDGGLPG IPGNPGLPGP KGEPGFHGFP GVQGPPGPPG SPGPALEGPK<br>GDGGLPGIPG N	Basement membrane

RAB1B_HUMAN	Ras-related protein Rab-1B	3	8	22.2	5.55	VVDNTTAKEF ADSLGIPFLE TSAK<br>VVDNTTAKEF ADSLGIPFLE TSAK<br>VVDNTTAKEF ADSLGIPFLE TSAK<br>EFADSLGIPF LETSAK<br>EFADSLGIPF LETSAK<br>EFADSLGIPF LETSAK<br>EFADSLGIPF LETSAK<br>NATNVEQAFM TMAAEIK	Membrane
ATP5H_HUMAN	ATP synthase subunit d	2	4	18.5	5.21	TIDWVAFAEI IPQNQK<br>TIDWVAFAEI IPQNQK<br>LAALPENPPA IDWAYYK<br>LAALPENPPA IDWAYYK	Mitochondrion inner membrane.
RAB7A_HUMAN	Ras-related protein Rab-7a	2	3	23.5	6.39	DPENFPFVVL GNK<br>EAINVEQAFQ TIAR<br>EAINVEQAFQ TIAR	Intracelluar membrane-bound organelle
CDC42_HUMAN	Cell division control protein 42 homolog precursor	2	3	21.3	5.76	TPFLLVGTQI DLRDDPSTIE K<br>TPFLLVGTQI DLRDDPSTIE K<br>TPFLLVGTQI DLR	Plasma membrane
COX2_HUMAN	Cytochrome c oxidase subunit 2	2	4	25.5	4.67	LLDVDNRVVL PIEAPIR<br>LLDVDNRVVL PIEAPIR<br>MMITSQDVLH SWAVPTLGLK<br>MMITSQDVLH SWAVPTLGLK	Membrane
TMEDA_HUMAN	Transmembrane emp24 domain-containing protein 10	2	3	25	6.98	ITDSAGHILY SKEDATK<br>RLEDLSESIV NDFAYMK	Integral to membrane

						RLEDLSESIV NDFAYMK	
SSB_HUMAN	Single-stranded DNA-binding protein	2	4	17.2	9.59	ESETTTSLVL ER	Mitochondion
						QATTIIADNI IFLSDQTK	
						QATTIIADNI IFLSDQTK	
						QATTIIADNI IFLSDQTK	
TIGD5_HUMAN	Tigger transposable element-derived protein 5	2	2	64.2	6.67	QKRHGISSQR FYGEAGPPAP SPAPGPPVKE EPALPSGAGP LPDR	Nucleus
						QKRHGISSQR FYGEAGPPAP SPAPGPPVKE EPALPSGAGP LPDR	
						LLPEQAAPPG AGDPGAGGCG RR	
F134B_HUMAN	Protein FAM134B	2	2	54.6	4.53	MASPAPPEHA EEGCPAPAAE EQAPPSPPPP QASPAERQQQ EEEAQEAGA	Membrane
						MASPAPPEHA EEGCPAPAAE EQAPPSPPPP QASPAERQQQ EEEAQ	
SRBP2_HUMAN	Sterol regulatory element-binding protein 2	2	2	123.6	8.72	TDGSPVMAAV QNPALTALTT PIQT	Membrane
						NPADPIAQVH QA	
SSRD_HUMAN	Translocon-associated protein subunit delta precursor	2	3	19	5.76	ETVFIVEISL TCKNRVQNMA LYADVGGKQF PVTRGQ	Integral to membrane
						NNEDISIIPP LFTVSVDHR	
						NNEDISIIPP LFTVSVDHR	
S10AB_HUMAN	Protein S100-A11	2	8	11.7	6.56	CIESLIAVFQ K	Cytoplasm
						CIESLIAVFQ K	
						CIESLIAVFQ K	
						TEFLSFMNTE LAAFTK	
						TEFLSFMNTE LAAFTK	
						TEFLSFMNTE LAAFTK	

						TEFLSFMNTE LAAFTK<br>TEFLSFMNTE LAAFTK	
NMES1_HUMAN	Normal mucosa of esophagus-specific gene 1 protein	2	5	9.6	9.45	KKNPEPWETV DPTVPQK	Nucleus
						LITINQQWKP IEELQNVQR<br>LITINQQWKP IEELQNVQR<br>LITINQQWKP IEELQNVQR<br>LITINQQWKP IEELQNVQR<br>LITINQQWKP IEELQNVQR	
CMC2_HUMAN	Calcium-binding mitochondrial carrier protein Aralar2	2	4	74.1	7.14	IAPLEEGTLP FNLAEAQR	Integral to plasam membrane
						IAPLEEGTLP FNLAEAQR<br>FGLGSVAGAV GATAVYPIDL VK<br>FGLGSVAGAV GATAVYPIDL VK	
PPCKM_HUMAN	Phosphoenolpyruvate carboxykinase [GTP]	2	3	70.6	7.56	LGTPVLQALG DGDFVK	Mitochondrion
						LGTPVLQALG DGDFVK<br>GVPLVYEAFN WR	
ACSL4_HUMAN	Long-chain-fatty-acid--CoA ligase 4	2	3	79.1	8.66	KVKALLGGNV RMMLSGGAPL SPQTHRFMNV CF	Integral to membrane
						KVKALLGGNV RMMLSGGAPL SPQTHRFMNV CF<br>LSPEPWTPET GLVTDAFK<br>LSPEPWTPET GLVTDAFK	
TFR1_HUMAN	Transferrin receptor protein 1	2	2	84.8	6.18	SAFSNLFGGE PLSYTR	Integral to membrane
						SSGLPNIPVQ TISR	
HS90A_HUMAN	Heat shock protein HSP 90-alpha	2	2	84.6	4.94	VILHLKEDQT EYLEER	Cytoplasm

						HLEINPDHSI IETLR	
HNRPU_HUMAN	Heterogeneous nuclear ribonucleoprotein U	2	3	90.5	5.76	SSGPTSLFAV TVAPPGAR	Cell surface
						SSGPTSLFAV TVAPPGAR	
						EKPYFPIPEE YTFIQNVPLE DR	
ITAV_HUMAN	Integrin alpha-V	2	2	116	5.45	STGLNAVPSQ ILEGQWAAR	Integral to plasma membrane
						IYIGDDNPLT LIVK	
HYOU1_HUMAN	Hypoxia up-regulated protein 1	5	10	111.3	5.16	LAGLFNEQR	Endoplasmic reticulum
						VEFEELCADL FER	
						VEFEELCADL FER	
						DAVVYPILVE FTR	
						DAVVYPILVE FTR	
						DAVVYPILVE FTR	
						DAVVYPILVE FTR	
						DAVVYPILVE FTR	
						LQDLTLRDLE KQEREK	
						LQDLTLRDLE KQER	
CLCN1_HUMAN	Chloride channel protein	2	2	108.7	5.68	AVIGAAALTG AVSH	Integral to plasma membrane
						ARLAGEGPPG APPG	
CO5A3_HUMAN	Collagen alpha-3(V) chain	2	2	171.9	6.37	DGIPGPLGPL GPPG	Extracellular matrix
						PGADGAQGR	
CODA1_HUMAN	Collagen alpha-1(XIII) chain	2	2	69.9	9.27	HGAKGAPGIA VAGM	Integral to membrane
						GSKGDPGMTG	
ZN503_HUMAN	Zinc finger protein 503	3	3	62.5	8.94	KEPGGGGGGG GGGGGGGGGV SS	Nucleus

						KEPGGGGGGG GGGGGGGG	
						EPGGGGGGGG GGGGGGGGV	
EWS_HUMAN	RNA-binding protein EWS	2	2	68.4	9.37	PLRGGPGGPG GPGGPMGR	Plasma membrane
						GGPGGPGGPG GPM	
FEZF2_HUMAN	Fez family zinc finger protein 2	2	2	48.8	9.52	SSLRAGGGGG GGGGGGGGGG GAPV	Nucleus
						SLRAGGGGGG GGGGGGGGGG APV	
LAMP2_HUMAN	Lysosome-associated membrane glycoprotein 2 precursor	2	3	44.9	5.34	GILTVDELLA IR	Plasma membrane
						GILTVDELLA IR	
						IPLNDLFR	
HXB3_HUMAN	Homeobox protein Hox-B3	3	3	44.3	9.27	EGCGGGGGGG GGGGSGGSGG GGGGGGGGDK	Nucleus
						GGGGGGSGGS GGGGGGGGGG DK	
						GGGSGGSGGG GGGGGGGDK	
CO2A1_HUMAN	Collagen alpha-1(II) chain	2	2	141.7	6.58	GPPGPAGAPG PQGFQ	Basement membrane
						PGVMGFPGPK GANGEPGK	
SODM_HUMAN	Superoxide dismutase [Mn]	2	5	24.7	8.35	GDVTAQIALQ PALK	Mitochondrion
						AIWNVINWEN VTER	
						AIWNVINWEN VTER	
						AIWNVINWEN VTER	
						AIWNVINWEN VTER	
CEAM1_HUMAN	Carcinoembryonic antigen-related cell adhesion molecule 1	2	4	57.5	5.65	TLTLLSVTR	Integral to plasma membrane
						TLTLLSVTR	
						TLTLLSVTR	

						NDTGPYECEI QNPVSANRSD PVTLNVTYGP DTP	
CABL1_HUMAN	CDK5 and ABL1 enzyme substrate 1	2	2	67.6	9.32	GGCIALAAPG TPAAGLAAGS<br>QSTGAVSLKE IIGLE	Cytoplasm
IRX3_HUMAN	Iroquois-class homeodomain protein IRX-3	2	2	52.1	4.88	AAELPIFPQL GAQYELK<br>RSPPGAGGSP PGAAVAPS	Nucleus
CTNA2_HUMAN	Catenin alpha-2	2	2	105.2	5.52	LLEPLVTQVT TLVNTSNK<br>MAATQIDSLC PQV	Plasma membrane
MUC16_HUMAN	Mucin-16	2	2	2351.9		MISAIPTLAV SPTVQGLVTS LVTS<br>SSVPTTSTGV VSEE	Integral to membrane
ATS14_HUMAN	ADAMTS-14 precursor	2	2	133.8	6.81	ASGPNPGPDP GPTSLPPFST PGSPLPGPQD PA<br>ASGPNPGPDP GPTSLPP	Extracellular matrix
BSN_HUMAN	Protein bassoon	2	2	416.2	7.28	KPLGSGPGPG PAPGAKTEPG AR<br>VPSAGADGPL AL	Cellskeletion
COBA1_HUMAN	Collagen alpha-1(XI) chain precursor	2	2	181	5.08	RGPQGPPGPP GDDGMR<br>AGPAGSPGED GDKGEIGEPG QK	Extracellular matrix
PCDAC_HUMAN	Protocadherin alpha-12	2	2	101.6	5.13	IYGELDFEEN NA<br>DAGVPPLGSN VTL	Cytoplasm.
SGOL1_HUMAN	Shugoshin-like 1	2	2	64.1	9.27	SSGMDPNSDD SSR<br>SLKDITNVSL YPVVK	Nucleus
LBXCO_HUMAN	Ladybird homeobox corepressor 1	2	2	99.8	6.11	GSGGQGKGGA GGGGGGGPGC GAEMAPGPPP HK<br>ASGPAGPGGP GGGAGVR	Nucleus

PAPD5_HUMAN	PAP-associated domain-containing protein 5	2	2	63.2	9.07	AEQRDFLPLE TTNNNNNHHQ PGAWAR	Cytoplasm
						AGSSASSPPS ASSSPHPSAA VPAADPADS	
RSMN_HUMAN	Small nuclear ribonucleoprotein-associated protein N;	2	2	24.6	11.2	AAGRGVPAGV PIPQ	Nucleus
						GTPIGMPPPG MRPP	
COBA2_HUMAN	Collagen alpha-2(XI) chain precursor	2	2	171.7		ERGLPGTAGG PGLK	Extracellular matrix
						GEMGIPGASG PIGP	
ACTG_HUMAN	Actin, cytoplasmic 2	8	23	41.8	5.31	EEEIAALVID NGSGMCK	Cytoplasm
						EEEIAALVID NGSGMCK	
						EEEIAALVID NGSGMCK	
						EEEIAALVID NGSGMCK	
						AVFPSIVGRP R	
						IWHHTFYNEL R	
						VAPEEHPVLL TEAPLNPK	
						VAPEEHPVLL TEAPLNPK	
						VAPEEHPVLL TEAPLNPK	
						LCYVALDFEQ EMATAASSSS LEK	
						LCYVALDFEQ EMATAASSSS LEK	
						LCYVALDFEQ EMATAASSSS LEK	
						LCYVALDFEQ EMATAASSSS LEK	
						LCYVALDFEQ EMATAASSSS LEK	
						LCYVALDFEQ EMATAASSSS LEK	
						LCYVALDFEQ EMATAASSSS LEK	
						LCYVALDFEQ EMATAASSSS LEK	
						FEQEMATAAS SSSLEK	
						SYELPDGQVI TIGNER	

						SYELPDGQVI TIGNER<br>SYELPDGQVI TIGNER<br>SYELPDGQVI TIGNER	
						CDVDIRKDLY ANTVLSGGTT MYPGIADR	
HNRPF_HUMAN	Heterogeneous nuclear ribonucleoprotein F	2	4	45.6	5.38	ITGEAFVQFA SQELAEK	Nucleus
						ITGEAFVQFA SQELAEK<br>ATENDIYNFF SPLNPVR<br>ATENDIYNFF SPLNPVR	
TOM40_HUMAN	Mitochondrial import receptor subunit TOM40 homolog	3	3	37.9	6.79	CKELFPIQME GVK	Integral to membrane
						QLSPTEAFPV LVGDMDNSGS LNAQVIHQLG PGLR<br>QLSPTEAFPV LVGDMDNSGS LNAQVIHQLG PGLR<br>SKMAIQTQQS KFVNWQVDGE YR	
OST48_HUMAN	Dolichyl-diphosphooligosaccharide--protein glycosyltransferase 48 kDa subunit	2	2	48.8	5.43	TLVLLDNLNV R	Integral to membrane
						NTLLIAGLQA R	
ZFHX4_HUMAN	Zinc finger homeobox protein 4	2	2	393.5	5.94	PGSATFGMPG MTGMAGSLLE DLK	Nucleus
						FLFSLTSPSI HFNDKDGDHD QSFYITDDPD DNADRSETSS IADPS	
SRRM2_HUMAN	Serine/arginine repetitive matrix protein 2	2	2	299.4	12.05	EQNSALPTSS QDEELM	Nucleus
						ASSWGGPHFS PEHK	
NPM_HUMAN	Nucleophosmin	2	4	32.6	4.64	MSVQPTVSLG GFEITPPVVL R	Nucleus

						MSVQPTVSLG GFEITPPVVL R	
						MSVQPTVSLG GFEITPPVVL R	
						SLGGFEITPP VVLR	
GP124_HUMAN	Probable G-protein coupled receptor 124	2	2	141.9	8.75	VPLGGGAPGT R	Integral to membrane
						REGGVPGTRP GSPGQNP	
PHB_HUMAN	Prohibitin	2	4	29.8	5.57	AAELIANSLA TAGDGLIELR	Integral to plasma membrane
						AAELIANSLA TAGDGLIELR	
						NITYLPAGQS VLLQLPQ	
						NITYLPAGQS VLLQLPQ	
PSPC1_HUMAN	Paraspeckle component 1	2	2	58.7	6.26	ATIPGPPMGP GPAMGPEGAA NMGTPMMPDN GAVHNDRFPQ GPPSQMG	Nucleus
						MGSRTGSETP QAPMSGVGPV SGGPGGFGRG SQGGNFEGPN KRR	
CO4A3_HUMAN	Collagen alpha-3(IV) chain	2	2	161.7	9.28	EEDIELDAKG DPGLPGAPGP QGLP	Basement membrane
						PGFFGFPGAM GPRGPKGHMG ERVIGHKGER GVKGLTG	
NELFA_HUMAN	Negative elongation factor A	2	2	59.4	9.06	PTPAAPTSPL TPTTPPAVAP TTQTPPVAMV APQTQAPAQQ QPK	Nucleus
						ADGQGSTTML VDTVFEMNYA T	
CO3A1_HUMAN	Collagen alpha-1(III) chain	2	2	138.5	6.21	GEPGPQGHAG	Extracellular matrix
						GLPGPPGSNG NPGPPGPSGS PGKDGPPGPA GNTGAP	
ODO1_HUMAN	2-oxoglutarate dehydrogenase E1 component	4	4	115.9	6.39	LAVQSLIRAY QIRGHHVAQL DPLGILDADL DSSVPADIIS STDKLGF	Mitochondrial membrane

						SLLRHPEARS SFDEMLPGTH FQRVIPEDGP AAQNPEN	
						LLDTAFDLDV FKNFS	
						LLDTAFDLDV FK	
COX5B_HUMAN	Cytochrome c oxidase subunit 5B	2	2	13.7	9.07	GLDPYNVLAP K	Membrane
						CGAHYKLVPQ QLAH	
PLEC1_HUMAN|	Plectin-1	3	5	531.4	5.73	CRPDQLTGLS LLPLSEK	Plasam membrane
						CRPDQLTGLS LLPLSEK	
						CRPDQLTGLS LLPLSEK	
						APVPASELLA SGVLSR	
						CITDPQTGLC LLPLKEK	
ITA2_HUMAN	Integrin alpha-2 precursor	3	6	129.2	5.16	YNVGLPEAK	Plasam membrane
						IFSGPSSEQF GYAVQQFINP K	
						IFSGPSSEQF GYAVQQFINP K	
						IFSGPSSEQF GYAVQQFINP K	
						FGIAVLGYLN R	
						FGIAVLGYLN R	
ITB1_HUMAN	Integrin beta-1	2	2	88.4	5.3	IGFGSFVEK	Plasma membrane
						NVLSLTNKGE VFNELVGK	
HS90B_HUMAN	Heat shock protein HSP 90-beta	4	6	83.2	4.97	ADLINNLGTI AK	Cytoplasm
						VILHLKEDQT EYLEER	
						SIYYITGESK EQVANSAFVE R	
						SIYYITGESK EQVANSAFVE R	
						HLEINPDHPI VETLR	

						HLEINPDHPI VETLR	
EFG1_HUMAN	Elongation factor G 1	3	4	83.4	6.58	AIYFDGDFGQ IVR	Mitochondrion
						AIYFDGDFGQ IVR	
						YGEIPAELR	
						VIGVLEPLDP EDYTKLEFSD ETFGSNIPK	
BC11B_HUMAN	B-cell lymphoma/leukemia 11B	2	2	95.5	6.1	RGNPMHRLLN PFQPSPKSPF LSTPPLPPMP PGGTPPPQPP AK	Nucleus
						ENGGGGVPGV PGAGGGAAKA	
GLU2B_HUMAN	Glucosidase 2 subunit beta	2	5	59.4	4.33	SLEDQVEMLR	Endoplasmic reticulum
						SLEDQVEMLR	
						SLEDQVEMLR	
						SLEDQVEMLR	
						LWEEQLAAAK	
CKAP4_HUMAN	Cytoskeleton-associated protein 4	4	5	66	5.63	VQSLQATFGT FESILR	Integral to membrane
						VQSLQATFGT FESILR	
						LALQALTEK	
						VQEQVHTLLS QDQAQAAR	
						TAVDSLVAYS VKIETNENNL ESAK	
						TAVDSLVAYS VKIETNENNL ESAK	
CNDD3_HUMAN	Condensin-2 complex subunit D3	2	2	168.8	7.38	PASQPPPQVR GSVMPSVIR	Nucleus
						DLLMEEDDMA LANVVMQEAQ KK	
TBB5_HUMAN	Tubulin beta chain	2	3	49.6	4.78	ALTVPELTQQ VFDAK	Microtubule
						ALTVPELTQQ VFDAK	
						YLTVAAVFR	

AL7A1_HUMAN	Alpha-aminoadipic semialdehyde dehydrogenase	2	2	55.3	6.44	IQVLGSLVSL EMGK	Mitochondrion
						QAVSMFLGAV EEAKK	
ECHB_HUMAN	Trifunctional enzyme subunit beta, mitochondrial	2	4	51.3	9.45	EVVDYIIFGT VIQEVK	Mitochondrion envelope
						EVVDYIIFGT VIQEVK	
						LAAAFAVSR	
						LAAAFAVSR	
SCOT_HUMAN	Succinyl-CoA:3-ketoacid-coenzyme A transferase 1	2	5	56.1	7.13	GLTAVSNNAG VDNFGLGLLL R	Mitochondrion
						GLTAVSNNAG VDNFGLGLLL R	
						GLTAVSNNAG VDNFGLGLLL R	
						GLTAVSNNAG VDNFGLGLLL R	
						AGGAGVPAFY TPTGYGTLVQ EGGSPIKYNK	
TBA1B_HUMAN	Tubulin alpha-1B chain	3	7	50.1	4.94	AVFVDLEPTV IDEVR	Microtubule
						AVFVDLEPTV IDEVR	
						AVFVDLEPTV IDEVR	
						QLFHPEQLIT GKEDAANNYA R	
						QLFHPEQLIT GKEDAANNYA R	
						LISQIVSSIT ASLR	
						LISQIVSSIT ASLR	
ITPR1_HUMAN	Inositol 1,4,5-trisphosphate receptor type 1	2	3	313.7	5.71	QDLDQLRSIV EKSELWVYKG QGPDETMDGA SGENEH 1162kkte	Membrane
						GAHAVVLELL QIPYEK	
						GAHAVVLELL QIPYEK	
EAA1_HUMAN	Excitatory amino acid transporter 1	2	2	59.5	8.52	MRMLQMLVLP LIISSLVTGM AALDSK	Integral to membrane
						TMVIVLTSVG LPTD	

LGI2_HUMAN	Leucine-rich repeat LGI family member 2	2	2	62.3	6.47	GGCGA	Extracellular region
						CTTTDFVVHQ TLPYQSVSVD TFNSK	
BIRC6_HUMAN	Baculoviral IAP repeat-containing protein 6	2	3	527.3	5.66	RLLDYVATVE DEAAAAK	Membrane fraction
						RLLDYVATVE DEAAAAK	
						AFHDMGGVQL ICNNMVTSTR	
						AIVNTARSMV	
RTN4_HUMAN	Reticulon-4	3	3	129.9	4.43	KPAAGLSAAP VPTAPAAGAP<br>LMDFGNDFVP PAPR	Plasam membrane
						GPLPAAPPVA PER	
						HQAQIDHYLG LANK	
ADCL1_HUMAN	Neutral cholesterol ester hydrolase 1	2	4	45.8	6.76	IVQELPQLLD AR	Membrane
						SAPLIADQAV LQLLPK	
						SAPLIADQAV LQLLPK	
						SAPLIADQAV LQLLPK	
CISY_HUMAN	Citrate synthase	3	5	51.7	8.45	ASASSTNLKD ILADLIPKEQ AR	Mitochondrion
						ASASSTNLKD ILADLIPKEQ AR	
						ASASSTNLKD ILADLIPK	
						ASASSTNLKD ILADLIPK	
						GLVYETSVLD PDEGIRFR	
SQRD_HUMAN	Sulfide:quinone oxidoreductase	6	9	49.9	9.18	QLSSSGRPTA SVIPSGVEWI K	Membrane
						QLSSSGRPTA SVIPSGVEWI K	
						QLSSSGRPTA SVIPSGVEWI K	
						ALQDFKEGNA IFTFPNTPVK	
						TSPVADAAGW VDVDKETLQH R	
						RYPNVFGIGD CTNLPTSK	
						TAAAVAQSG ILDR	

						TAAAVAAQSG ILDR	
						VILAEFDYKA EPLETFPFDQ SK	
ACTC_HUMAN	Actin, alpha cardiac muscle 1	2	2	42	5.23	AGFAGDDAPR	Cytoplasm
						AGFAGDDAPR	
						AGFAGDDAPR	
						AGFAGDDAPR	
						AGFAGDDAPR	
						AGFAGDDAPR	
						AGFAGDDAPR	
						AGFAGDDAPR	
						AGFAGDDAPR	
						AGFAGDDAPR	
						AGFAGDDAPR	
						AGFAGDDAPR	
						AGFAGDDAPR	
						AGFAGDDAPR	
						AGFAGDDAPR	
						AVFPSIVGRP R	
						AVFPSIVGRP R	
						AVFPSIVGRP R	
						IWHHTFYNEL R	
						IWHHTFYNEL R	
						IWHHTFYNEL R	
						LDLAGRDLTD YLMK	
						EKLCYVALDF ENEMATAASS SSLEK	
						LCYVALDFEN EMATAASSSS LEK	
						SYELPDGQVI TIGNER	
						SYELPDGQVI TIGNER	
						MQKEITALAP STMK	

						EITALAPSTM K	
						EITALAPSTM K	
PHB2_HUMAN	Prohibitin-2	4	5	33.3	9.83	IGGVQQDTIL AEGLHFR	Membrane
						VLSRPNAQEL PSMYQR	
						QKIVQAEGEA EAAK	
						IYLTADNLVL NLQDESFTR	
						IYLTADNLVL NLQDESFTR	
ATPG_HUMAN	ATP synthase subunit gamma	2	3	33	9.23	QAVITKELIE IISGAAALD	Mitochondrial inner membrane.
						ELIEIISGAA ALD	
						ELIEIISGAA ALD	
ANK3_HUMAN	Ankyrin-3	3	3	480.1	6.12	AAPILSKTKN GLSPLHMATQ GDHLNCVQ	Membrane
						AAPILSKTKN GLSPLHMATQ GDHLNCVQ	
						ELESNGSGKD NEFGLGLDSP QNEIAQNGNN DQSITECSIA	
						FMLLKKWVTR DGKNATTDAL TSVLTKINR	
CATD_HUMAN	Cathepsin D	2	3	44.5	6.1	ISVNNVLPVF DNLMQQK	Mitochondrion
						ISVNNVLPVF DNLMQQK	
						LVDQNIFSFY LSR	
ETFB_HUMAN	Electron transfer flavoprotein subunit beta	2	3	27.8	8.25	LGPLQVAR	Mitochondrion
						KIEVIKPGDL GVDLTSK	
						KIEVIKPGDL GVDLTSK	
MTHR_HUMAN	Methylenetetrahydrofolate reductase	2	2	74.5	5.22	VNRQGILTIN SQPNINGKPS SDPIVGWGPS GGYVFQKAYL E	Cytosol

						TIIQYIHDNY FLVNLVDNDF PLDNCLWQVV EDTLE	
PGRC1_HUMAN	Membrane-associated progesterone receptor component 1	2	2	21.7	4.56	GRKFYGPEGP YGVFAGR	Integral to membrane
						FYGPEGPYGV FAGR	
RAB14_HUMAN	Ras-related protein Rab-14	2	2	23.9	5.85	TGENVEDAFL EAAKK	Plasam membrane
						TGENVEDAFL EAAK	
ATPO_HUMAN	ATP synthase subunit O	2	3	23.3	9.97	LVRPPVQVYG IEGR	Mitochondrion inner membrane
						LVRPPVQVYG IEGR FSPLTTNLIN LLAENGR	
PRDX3_HUMAN	Thioredoxin-dependent peroxide reductase	3	5	27.7	7.68	DYGVLLEGSG LALR	Mitochondrion
						DYGVLLEGSG LALR PNGVIKHLSV NDLPVGR HLSVNDLPVG R HLSVNDLPVG R	
